# Long-term neurodevelopmental outcomes after vacuum-assisted delivery: A population-based cohort study

**DOI:** 10.1371/journal.pmed.1004825

**Published:** 2026-07-17

**Authors:** Ida Björk, Jenny Bolk, Gunilla Ajne, Ängla Mantel

**Affiliations:** 1 Department of CLINTEC, Division of Obstetrics and Gynecology, Karolinska Institute, Stockholm, Sweden; 2 Theme Women’s Health, Department of Obstetrics, Karolinska University Hospital, Stockholm, Sweden; 3 Department of Medicine Solna, Division of Clinical Epidemiology, Karolinska Institutet, Stockholm, Sweden; 4 Department of Clinical Science and Education Södersjukhuset, Karolinska Institutet, Stockholm, Sweden; 5 Sachs’ Children and Youth Hospital, Södersjukhuset, Stockholm, Sweden; University of Cambridge School of Clinical Medicine, UNITED KINGDOM OF GREAT BRITAIN AND NORTHERN IRELAND

## Abstract

**Background:**

Vacuum-assisted delivery (VAD) is widely used in obstetric care, yet concerns remain regarding potential long-term neurodevelopmental sequelae in offspring, particularly after procedures performed at higher fetal head stations. Evidence on long-term outcomes after VAD remains limited. Given the safety concerns surrounding VAD, we investigated neonatal and long-term neurodevelopmental outcomes after VAD stratified by fetal head station, using emergency cesarean delivery (ECD) and spontaneous vaginal delivery as reference groups.

**Methods and findings:**

In this population-based cohort study, we obtained data from nationwide health registers and included all singleton births to primiparous women in Sweden between 1997 and 2014, with follow-up through December 31, 2021. Differences in neonatal outcomes across delivery modes were estimated using multivariable logistic regression models to obtain odds ratios (ORs). All children were followed longitudinally for adverse long-term neurodevelopmental outcomes—a diagnosis of attention deficit/hyperactivity disorder [ADHD], autism spectrum disorder [ASD], cerebral palsy [CP], epilepsy [EP], or intellectual disability [ID]. Rates of long-term outcomes were compared using multivariable Cox regression models to obtain hazard ratios (HRs). All analyses were stratified by child sex. Analyses of neonatal outcomes were adjusted for maternal age, body mass index (BMI), gestational age, pre-eclampsia, gestational diabetes, and diabetes mellitus type I/II. Long-term analyses were additionally adjusted for smoking status, child’s birth year, chorioamnionitis, maternal education level, and maternal psychiatric comorbidity. Among 630,985 term singleton births, median follow-up was 13–14 years. Compared with ECD, mid/low VAD was associated with increased odds of neonatal intracranial hemorrhage (traumatic aOR 7.20, 95% CI [2.97, 17.45]; non-traumatic aOR 3.65, 95% CI [2.41, 5.52]; subgaleal hematoma aOR 3.35, 95% CI [2.68, 4.20]; and neonatal seizures aOR 1.85, 95% CI [1.52, 2.26]), but with lower odds of meconium aspiration (aOR 0.53, 95% CI [0.42, 0.67]). In long-term follow-up, mid/low VAD showed no increased risks for ADHD, ASD, CP, or epilepsy, and a reduced risk of intellectual disability (aHR 0.80, 95% CI [0.67, 0.96]) as compared with ECD. Outlet VAD—similar to spontaneous vaginal delivery—was associated with lower risks of adverse neonatal and long-term neurodevelopmental outcomes compared with ECD. The main limitations of this study were the lack of information on the indication for intervention and whether emergency cesarean deliveries were performed in the first or second stage of labor.

**Conclusions:**

In this nationwide cohort, mid/low VAD was associated with higher neonatal morbidity but no increased risk of long-term neurodevelopmental disorders. Outlet VAD showed no signal of adverse long-term neurodevelopmental outcomes. These findings provide reassuring evidence regarding the long-term safety of VAD when performed appropriately and help address an important knowledge gap regarding long-term outcomes after VAD. However, comparisons between delivery modes should be interpreted cautiously, as the clinical circumstances leading to VAD and ECD differ and are incompletely captured in register data. The findings should therefore not be interpreted as evidence to guide acute choice of delivery mode.

## Author summary

**Why was this study done?:**

- Despite widespread use of vacuum-assisted delivery (VAD), concerns remain regarding possible long-term neurodevelopmental consequences for children, particularly after procedures performed at higher fetal head stations.
- Existing evidence on long-term outcomes after VAD is limited, and few studies have distinguished between outlet and mid/low procedures.
- We therefore investigated neonatal and long-term neurodevelopmental outcomes after VAD, stratified by fetal head stations, in a large nationwide cohort.

**What did the researchers do and find?:**

- We used nationwide Swedish registry data including more than 600,000 births and followed the children for up to 24 years.
- We studied both short-term neonatal outcomes and long-term neurodevelopmental disorders after VAD, distinguishing between outlet and mid/low VAD.
- Children born after outlet VAD had lower risks of attention-deficit/hyperactivity disorder, autism spectrum disorder, intellectual disability, and epilepsy when compared to children born after emergency cesarean delivery.
- Mid/low VAD was not linked to increased long-term risks, although some neonatal complications were more frequent.

**What do these findings mean?:**

- These findings provide reassuring evidence regarding the long-term safety of VAD when performed appropriately.
- Although some neonatal complications were more frequent after mid/low VAD, these risks did not appear to translate into increased long-term neurodevelopmental morbidity.
- Because the clinical circumstances leading to VAD and emergency cesarean delivery differ and are incompletely captured in register data, these findings should not be interpreted as evidence to guide acute choice of delivery mode. Rather, they help address an important knowledge gap regarding the long-term safety of VAD.
- A limitation of this study is that information on why an intervention was performed and whether emergency cesarean deliveries occurred before or after full cervical dilatation was not available, making some comparisons between delivery modes difficult to interpret.

## Introduction

Ensuring a safe delivery is crucial for minimizing neonatal complications, reducing infant morbidity, and preventing long-term adverse health outcomes. The second stage of labor represents a particularly vulnerable period for the fetus, during which complications are most likely to arise [1,2]. Operative delivery, including vacuum-assisted delivery (VAD), may be required in cases of fetal distress or dystocia.

In Sweden, VAD accounts for 6%–9% of all deliveries, whereas forceps are rarely used [3]. VAD performed with the fetal head at outlet station is typically a brief procedure with minimal risk of injury in term-born infants and represents approximately half of all VADs [4]. However, several studies indicate a risk of severe neonatal complications, including intracranial hemorrhage, seizures and death, associated with VAD performed at mid/low station [5–10]. These differences may reflect a more complex procedure, for example shown as higher traction forces in mid/low VAD than at the outlet station [11,12].

Despite observations of unfavorable neonatal outcomes in mid/low VAD, research on long-term neurodevelopmental outcomes remains limited and largely based on outdated cohorts [13–15]. Some recent studies report no significant associations between VAD and adverse long-term neurodevelopmental outcomes, although most have not distinguished between outlet and mid/low station procedures [16–19]. Consequently, important knowledge gaps remain regarding the long-term safety of VAD, particularly according to fetal head station.

From a global maternal health perspective, maintaining access to safe instrumental vaginal delivery remains important, particularly in settings with limited surgical capacity and high maternal morbidity [20]. Clarifying the long-term safety of VAD is therefore essential to support safe obstetric practice and sustain competence in instrumental vaginal delivery worldwide.

Given these knowledge gaps, this study aimed to investigate neonatal and long-term neurodevelopmental outcomes after VAD, stratified by fetal station, in a nationwide population-based cohort. Emergency cesarean delivery (ECD) and spontaneous vaginal delivery were included as reference groups to contextualize outcomes after VAD.

## Methods

### Study design, setting and data sources

This nationwide cohort study was conducted in Sweden, where the personal identification number (PIN) assigned to each resident enables complete linkage across population-based health and demographic registers [21]. The country’s tax-funded healthcare system ensures universal access to standardized, high-quality maternal and neonatal care, with nearly all deliveries occurring in hospital settings. These features provide an ideal infrastructure for unbiased, long-term follow-up in perinatal epidemiology [22].

The study base was identified through the *Swedish Medical Birth Register*, which has prospectively collected data on pregnancies, deliveries, and neonatal outcomes since 1973, covering more than 98% of all births in Sweden. The register has been validated and demonstrates high completeness and accuracy for key perinatal variables [23].

Using the PIN, we linked data from the Medical Birth Register with several nationwide health and demographic registers to define exposures, outcomes, and covariates of interest. These included the *National Patient Register,* which contains International Classification of Diseases (ICD)-coded diagnoses from inpatient (full coverage since 1987) and specialized outpatient (since 2001) care; the *Prescribed Drug Register,* capturing all dispensed prescriptions since 2005 [24]; the *Cause of Death Register*, recording dates underlying causes of death [25]; the *Total Population Register*, providing demographic information [22]; the *Longitudinal Integration Database for Health Insurance and Labor Market Studies (LISA)***,** which includes socioeconomic information, such as income and educational level of all residents [26]. These registers have been extensively validated, and are characterized by near-complete national coverage and high diagnostic accuracy, providing a uniquely robust platform for population-based research using real-world data.

### Study participants

The study base included all singleton term births (≥37 + 0 gestational weeks) to primiparous women registered in the Medical Birth Register between 1997 and 2014. The study period ensured that all children reached at least 7 years of age, allowing sufficient time for reliable ascertainment of outcomes. Children born preterm, in breech presentation, delivered by forceps, or with congenital malformations were excluded.

### Exposure

The exposed cohort comprised children born via VAD, further classified as outlet VAD or mid/low VAD according to surgical procedure code in the birth register (see Table A in S1 Appendix). In the Swedish classification, VAD is defined by fetal head station: above the ischial spine as high station, from the ischial spine to just above the pelvic floor as mid/low, and at the pelvic floor as outlet. This differs slightly from international classification, where mid and low stations are reported as separate categories.

### Comparison cohorts

Children born via VAD were compared with two reference groups: (i) those delivered by ECD, and (ii) those delivered by spontaneous vaginal delivery. Mode of delivery was identified through standardized registration in the birth register. This dual-comparator design enabled evaluation of VAD outcomes in relation to both surgical intervention and unassisted vaginal birth.

### Outcomes and follow-up

#### Neonatal outcomes.

Severe neonatal outcomes were identified using ICD-10 diagnosis codes recorded in the Medical Birth Register or the National Patient Register. Outcomes included asphyxia, intracerebral hemorrhage (traumatic and non-traumatic), cephalohematoma, subgaleal hematoma, meconium aspiration, respiratory distress, neonatal seizures, and ischemic stroke. These diagnoses represent clinically significant neonatal complications with potential implications for both acute and long-term morbidity. Please refer to Table A in S1 Appendix for the ICD codes used.

### Long-term neurodevelopmental outcomes

All children were followed for the occurrence of attention deficit/hyperactivity disorder (ADHD), autism spectrum disorder (ASD), cerebral palsy (CP), epilepsy (EP), and intellectual disability (ID). Each outcome was defined as the presence of at least two registered diagnoses in the National Patient Register. For ADHD, dispensed prescriptions were also considered: ADHD was defined as either (i) two registered diagnoses or (ii) one registered diagnosis in combination with a dispensed prescription for ADHD medication. For ICD codes and Anatomical Therapeutic Chemical (ATC) classifications, see Table A in S1 Appendix. Follow-up began at disorder-specific ages to avoid misclassification from early or unreliable diagnoses: from 3 months of age for CP, EP, and ID; from 1 year for ASD; and from 3 years for ADHD. For EP, this approach ensured the exclusion of neonatal seizures. The follow-up period extended from the start of follow-up until the respective endpoint, death, migration, or 31 December 2021, whichever occurred first. Overall follow-up ranged from 7 to 24 years.

### Statistical analysis

This study is reported as per the Strengthening the Reporting of Observational Studies in Epidemiology (STROBE) guideline (S1 Checklist). The study design and statistical analyses were specified prospectively in an analysis plan developed before data analysis (S1 Protocol). Additional analyses and reporting modifications introduced during manuscript development and peer review, including comparison of births with known and unspecified fetal head station, cumulative incidence functions, assessment of the proportional hazards assumption, and presentation of absolute risks, were undertaken to assess the robustness and interpretation of the findings.

Baseline characteristics were summarized as means (SD) for continuous variables and proportions for categorical variables. Logistic regression models were used to estimate odds ratios (ORs) with 95% confidence intervals (CIs) for associations between mode of delivery and adverse neonatal outcomes. For long-term neurodevelopmental outcomes, incidence rates were calculated per 1,000 person-years, and Cox proportional hazards regression models were applied to estimate hazard ratios (HRs) with 95% CIs. Cumulative incidence functions (CIFs) were estimated to illustrate how risk accumulated over time while accounting for competing events and are presented in Fig A in the S1 Appendix. Visual inspection of the CIFs showed broadly parallel trajectories, with only small absolute differences in risk over time. The proportional hazards assumption was evaluated by including time-dependent interaction terms between the exposure and log(follow-up time). These tests provided no evidence of departures from proportionality (*p*-values [0.06, 0.95]) (Table B in the S1 Appendix).

For neonatal outcomes, multivariable models were adjusted for maternal age, body mass index (BMI), gestational age, preeclampsia, gestational diabetes, and diabetes mellitus type I/II.

All multivariable models for long-term outcomes were adjusted for pre-specified covariates identified a priori from established literature. Confounder adjustment was performed sequentially in three steps. Model 1 included maternal age, smoking status, BMI, diabetes mellitus type I/II, and child’s birth year. Model 2 additionally included preeclampsia, gestational diabetes, and chorioamnionitis. Model 3 further included maternal education level and maternal psychiatric comorbidity (ADHD, ASD, depression, and anxiety). Analyses were conducted in the overall cohort and stratified by child sex. All analyses were performed using complete case methodology; individuals with missing information on any covariate included in the multivariable models were excluded from the respective analyses.

Several pre-defined sensitivity analyses were undertaken to assess the robustness of findings: (i) exclusion of children diagnosed with perinatal asphyxia (definition in Table A in the S1 Appendix); (ii) stratification by gestational age (early term [37 + 0–38 + 6], full term [39 + 0–40 + 6], late term [41 + 0–41 + 6], and post-term [≥42 + 0]); (iii) stratification by birth period (1997–2006 and ≥2007); and (iv) stratification by birthweight category (small, appropriate, and large for gestational age).

All analyses were performed using SAS statistical software, version 9.4 (SAS Institute, Cary, NC, USA).

### Patient and public involvement

Patients and/or the public were not involved in the design, conduct, reporting, or dissemination plans of this research.

### Ethics

Ethical approval for the study was obtained from the Swedish Ethical Review Authority (Ref. no. 2022-02513-01). The requirement for informed consent was waived by the Swedish Ethical Review Authority because this was a nationwide register-based study using de-identified data.

### Use of artificial intelligence tools

Artificial intelligence tools (ChatGPT, OpenAI) were used to assist with language editing and improving readability of the manuscript. AI tools were not used for data analysis, interpretation of results, or generation of scientific conclusions.

## Results

### Study participants and baseline characteristics

**Fig 1:**
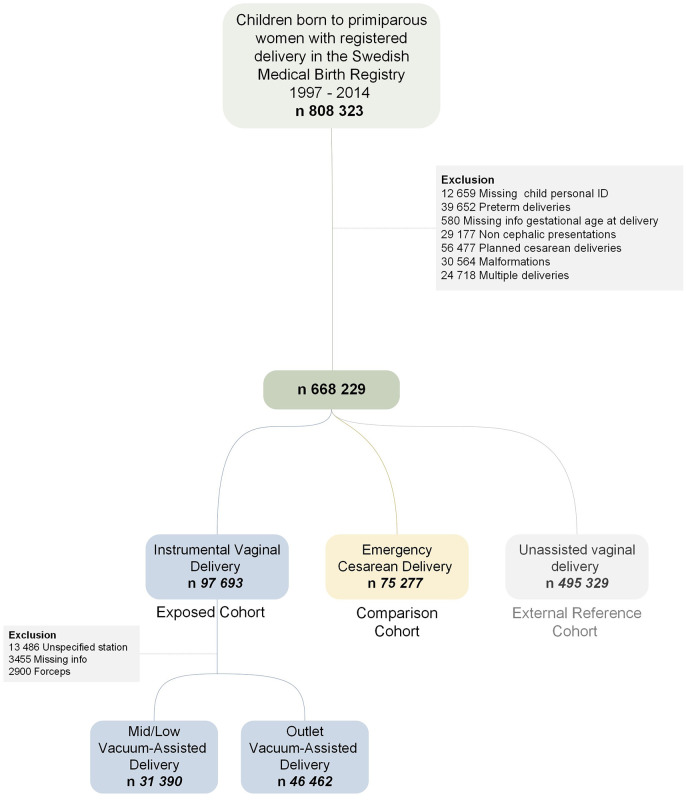
Flowchart of study population. Flowchart illustrating the study population generation. Study base consist of all term-born children to primiparous women in Sweden between 1997 and 2014. After applying exclusion criteria, the final study population consist of one exposed cohort including children born via vacuum-assisted delivery, one unexposed comparator cohort including children born via emergency cesarean delivery, and one external reference cohort consisting of children born via spontaneous vaginal delivery.

Of 630,985 singleton term births with cephalic presentation to primiparous women between 1997 and 2014, 73,215 (11.6%) were delivered by vacuum-assisted delivery (VAD), 73,739 (11.7%) by ECD, and 484,031 (76.7%) by spontaneous vaginal delivery (Fig 1). Among VAD births, 31,014 (42.4%) were classified as mid/low station and 42,201 (57.6%) as outlet VAD.

Baseline maternal and neonatal characteristics by mode of delivery are shown in Table 1. Mothers who delivered by VAD or ECD were generally older than those who delivered by spontaneous vaginal delivery. The prevalence of maternal overweight and obesity was highest in the ECD group, whereas mean BMI was similar between VAD and spontaneous vaginal deliveries. Late-term gestational age (41 + 0 to 41 + 6 weeks) occurred more often among infants born by VAD (25.7%) and ECD (26.7%) than by spontaneous vaginal delivery (20.0%), while post-term births (≥42 + 0 weeks) were most frequent in the ECD group (22%), followed by VAD (13%) and spontaneous vaginal delivery (8%).

**Table 1:** Baseline maternal characteristics by mode of delivery among primiparous women, Sweden 1997-2014.

		Vacuum-Assisted Delivery				
	Emergency cesarean Delivery *n* = 73,739	All *n* = 73,215		Outlet *n* = 42,201	Mid/Low *n* = 31,014	Spontaneous Vaginal Delivery *n* = 484,031
Maternal age years						
Mean ± SD	29.8 ± 5.1	29.1 ± 4.9		28.7 ± 4.9	29.6 ± 4.9	27.6 ± 4.9
*n* (%)						
<18	237 (0.3)	281 (0.4)		196 (0.5)	85 (0.3)	4,360 (0.9)
18–24	11,187 (15.2)	12,913 (17.6)		8,258 (19.6)	4,655 (15.0)	129,318 (26.7)
25–29	18,158 (24.6)	19,950 (27.3)		11,955 (28.3)	7,995 (25.8)	145,343 (30.0)
30–34	26,886 (36.5)	26,876 (36.7)		15,005 (35.6)	11,871 (38.3)	150,773 (31.2)
35–39	13,381 (18.2)	10,834 (14.8)		5,604 (13.3)	5,230 (16.9)	46 080 (9.5)
≥40	3,890 (5.3)	2,361 (3.2)		1,183 (2.8)	1,178 (3.8)	8,157 (1.7)
BMI early pregnancy^†^						
Mean ± SD	25.5 ± 4.9	23.8 ± 4.1		23.8 ± 4.0	24.1 ± 4.1	23.8 ± 4.1
*n* (%)						
Underweight	1,009 (1.4)	1910 (2.6)		1,261 (3.0)	649 (2.1)	13 526 (2.8)
Normal weight	35,450 (48.1)	43,573 (59.5)		25,570 (60.6)	18,003 (58.1)	292,714 (60.5)
Overweight	16,682 (22.3)	13,304 (18.1)		7,473 (17.7)	5,831 (18.8)	83,101 (17.2)
Obesity, class 1	8,701 (11.8)	5,094 (7.0)		2,779 (6.6)	2,315 (7.5)	33 456 (6.9)
Obesity, >class 2	4,206 (5.7)	1893 (2.6)		1,035 (2.7)	858 (2.8)	12 617 (2.6)
Missing	7,691 (10.4)	7,441 (10.2)		4,083 (9.7)	3,358 (10.8)	48,617 (10.0)
Smoking early pregnancy^‡^	5,255 (7.1)	4,677 (6.4)		2,869 (6.8)	1808 (5.8)	37 952 (7.8)
Missing	3,945 (5.4)	3,725 (5.1)		1943 (4.6)	1,782 (8.4)	23 712 (4.9)
Gestational week of delivery						
37 + 0–38 + 6	8,664 (11.8)	8,527 (11.7)	5,207 (12.3)		3,320 (10.7)	81,060 (16.8)
39 + 0–40 + 6	29,025 (39.4)	36,446 (49.8)	21,370 (50.6)		15,076 (48.6)	268,452 (55.5)
41 + 0–41 + 6	19,740 (26.8)	18,804 (25.7)	10,564 (25.0)		8,240 (26.6)	96,961 (20.0)
≥42 + 0	16,310 (22.1)	9,438 (12.9)	5,060 (12.0)		4,378 (14.1)	37,558 (7.8)
Adverse pregnancy outcomes						
Pregnancy-induced hypertensive disorder^§^	5,472 (7.4)	2,593 (3.5)	1,491 (3.5)		1,102 (3.6)	13 542 (2.8)
Gestational diabetes^§^	940 (1.3)	567 (0.8)	321 (0.8)		246 (0.8)	3,217 (0.7)
Pre-pregnancy comorbidities^¶^						
Diabetes mellitus type I/II^c^	762 (1.0)	284 (0.4)	151 (0.4)		133 (0.4)	838 (0.2)
ADHD	273 (0.4)	195 (0.3)	117 (0.3)		78 (0.3)	1988 (0.4)
Autism spectrum disorder^c^	49 (0.1)	53 (0.1)	31 (0.1)		22 (0.1)	429 (0.1)
Maternal depression^c^	2,321 (3.2)	1903 (2.6)	1,071 (2.5)		832 (2.7)	13 067 (2.7)
Maternal anxiety disorder^c^	2,134 (2.9)	1,773 (2.4)	1,004 (2.4)		769 (2.5)	11 448 (2.4)
Alcohol and/or illicit drug use^b^	1,586 (2.2)	1,346 (1.8)	762 (1.8)		584 (1.9)	10 519 (2.2)

† BMI categories classified according to WHO criteria: underweight <18.5 kg/m^2^; normal weight 18.5–24.9 kg/m^2^; overweight 25.0–29.9 kg/m^2^; obesity class I 30.0–34.9 kg/m^2^; and obesity class II or higher ≥35.0 kg/m^2^.

‡ Smoking during early pregnancy as reported by the midwife at the first antenatal care visit, recorded in the Swedish Medical Birth Register.

§ Based on registered diagnoses according to ICD codes in the Swedish Medical Birth Register.

¶ Based on registered diagnoses according to ICD codes in the National Patient Register before pregnancy.

Percentages are calculated within each delivery mode column.

ADHD, Attention-deficit/Hyperactivity disorder.

Exposure to maternal pre-eclampsia and diabetes (any type) was more common among children delivered by ECD than by VAD or spontaneous vaginal delivery. The prevalence of maternal psychiatric comorbidities—including depression, anxiety, ADHD, and autism spectrum disorder—was similar across delivery modes.

A subset of vacuum-assisted deliveries lacked information on fetal head station and were excluded, as station was required to define the exposure (outlet vs mid/low VAD). Baseline characteristics were largely similar between births with known and unspecified station, with differences within the range observed across VAD subgroups and no pattern suggesting systematic selection (Table C in S1 Appendix).

### Neonatal characteristics and odds of adverse neonatal outcomes by delivery mode

Neonatal characteristics are presented in Table 2, and ORs of adverse neonatal outcomes by mode of delivery are shown in Table 3. Overall, adverse neonatal outcomes were more frequent after vacuum-assisted delivery (VAD) than after ECD.

**Table 2:** Neonatal characteristics by mode of delivery among singleton term births to primiparous women, Sweden, 1997–2014.

		Vacuum-Assisted Delivery			
	Emergency cesarean Delivery *n* = 73,739	All *n* = 73,215	Outlet *n* = 42,201	Mid/Low *n* = 31,014	Spontaneous Vaginal Delivery *n* = 484,031
Sex *n* (%)					
Male	41,682 (56.52)	40,917 (55.91)	23,392 (55.43)	17,525 (56.51)	239,441 (49.48)
Female	32,057 (43.48)	32,298 (44.09)	18,809 (44.57)	13,489 (43.49)	244,590 (50.52)
Birthweight *n* (%)^†^					
SGA	3,150 (4.27)	2,221 (3.03)	1,404 (3.33)	817 (2.63)	14 050 (2.90)
AGA	66,521 (90.21)	69,342 (94.71)	39,980 (94.74)	29,362 (94.67)	462,499 (95.55)
LGA	3,895 (5.28)	1,517 (2.07)	751 (1.78)	766 (2.47)	6,723 (1.39)
Missing	173 (0.23)	135 (0.18)	66 (0.16)	69 (0.22)	759 (0.16)
Macrosomia	5,651 (7.66)	2,171 (2.97)	1,060 (2.51)	1,111 (3.58)	8,496 (1.76)
Missing	157 (0.21)	131 (0.14)	63 (0.15)	68 (0.22)	700 (0.14)

† Birthweight for gestational age classified according to Swedish national reference standards: small for gestational age (SGA) defined as <–2 SD from the mean, appropriate for gestational age (AGA) as within ±2 SD, and large for gestational age (LGA) as>+2 SD.

Percentages are calculated within each delivery mode column.

SGA, Small for gestational age; AGA, Appropriate for gestational age; LGA, Large for gestational age.

**Table 3:** Unadjusted and adjusted odds ratios (95% CI) for neonatal adverse outcomes by mode of delivery among term singleton births to primiparous women, Sweden, 1997–2014. Vacuum-assisted delivery, by fetal station, and spontaneous vaginal delivery compared with emergency cesarean delivery.

		Vacuum-Assisted Delivery			
	Emergency cesarean Delivery *n* = 73 739	All *n* = 73 215	Outlet *n* = 42 201	Mid/Low *n* = 31 014	Spontaneous Vaginal Delivery *n* = 484 031
Asphyxia *n* (%)	2,727 (3.70)	2,169 (2.96)	930 (2.20)	1,239 (3.99)	2,367 (0.49)
OR_cr_ [95% CI]	Ref. 1.0	0.80 [0.75,0.84]	0.59 [0.54,0.63]	1.08 [1.01,1.16]	0.13 [0.12,0.14]
OR_adj_ [95% CI]	Ref. 1.0	0.88 [0.83, 0.94]	0.65 [0.60, 0.71]	1.18 [1.10,1.27]	0.15 [0.14, 0.16]
Traumatic intracranial haemorrhage *n* (%)	7 (0.01)	34 (0.05)	13 (0.03)	21 (0.07)	18 (0.00)
OR_cr_ [95% CI]	Ref. 1.0	4.89 [2.17,11.03]	3.25 [1.30,8.14]	7.14 [3.03,16.79]	0.39 [0.16,0.94]
OR_adj_ [95% CI]	Ref. 1.0	4.99 [2.15, 11.58]	3.28 [1.25, 8.61]	7.20 [2.97, 17.45]	0.43 [0.17, 1.08]
Non-traumatic intracranial haemorrhage n (%)	46 (0.06)	92 (0.13)	31 (0.07)	61 (0.20)	151 (0.03)
OR_cr_ [95% CI]	Ref. 1.0	2.02 [1.42,2.87]	1.18 [0.75,1.86]	3.16 [2.15,4.63]	0.50 [0.36,0.70]
OR_adj_ [95% CI]	Ref. 1.0	2.35 [1.60, 3.45]	1.36 [0.83,2.23]	3.65 [2.41,5.52]	0.61 [0.42, 0.88]
Cephalohematoma *n* (%)	602 (0.82)	3,646 (4.98)	1997 (4.73)	1,647 (5.32)	5,154 (1.06)
OR_cr_ [95% CI]	Ref. 1.0	6.37 [5.84, 6.95]	6.04 [5.51,6.62]	6.82 [6.21,7.50]	1.31 [1.20, 1.42]
OR_adj_ [95% CI]	Ref. 1.0	6.52 [5.94, 7.15]	6.18 [5.60, 6.81]	6.97 [6.30, 7.70]	1.36 [1.24, 1.48]
Subgaleal hematoma, *n* (%)	142 (0.19)	300 (0.41)	84 (0.20)	216 (0.70)	41 (0.01)
OR_cr_ [95% CI]	Ref. 1.0	2.13 [1.75,2.60]	1.03 [0.79,1.35]	3.64 [2.94,4.49]	0.04 [0.03,0.06]
OR_adj_ [95% CI]	Ref. 1.0	1.98 [1.60, 2.45]	0.96 [0.72, 1.27]	3.35 [2.68, 4.20]	0.04 [0.03, 0.05]
Meconium aspiration, *n* (%)	479 (0.65)	199 (0.27)	112 (0.27)	87 (0.28)	628 (0.13)
OR_cr_ [95% CI]	Ref. 1.0	0.42 [0.35,0.49]	0.41 [0.33,0.50]	0.43 [0.34,0.54]	0.20 [0.18,0.22]
OR_adj_ [95% CI]	Ref. 1.0	0.53 [0.44, 0.63]	0.53 [0.42, 0.65]	0.53 [0.42, 0.67]	0.28 [0.25, 0.32]
Respiratory distress *n* (%)	3,762 (5.10)	2,745 (3.75)	1,304 (3.09)	1,441 (4.65)	8,078 (1.67)
OR_cr_ [95% CI]	Ref. 1.0	0.73 [0.69,0.76]	0.59 [0.56,0.63]	0.91 [0.85,0.97]	0.32 [0.30,0.33]
OR_adj_ [95% CI]	Ref. 1.0	0.79 [0.74, 0.83]	0.65 [0.61, 0.70]	0.96 [0.90, 1.03]	0.36 [0.34, 0.38]
Seizures, *n* (%)	275 (0.37)	306 (0.42)	117 (0.28)	189 (0.61)	417 (0.09)
OR_cr_ [95% CI]	Ref. 1.0	1.12 [0.95,1.32]	0.74 [0.60,0.92]	1.64 [1.36,1.97]	0.23 [0.20,0.27]
OR_adj_ [95% CI]	Ref. 1.0	1.30 [1.09, 1.55]	0.87 [0.69,1.10]	1.85 [1.52,2.26]	0.28 [0.23, 0.33]
Ischemic stroke, *n* (%)	21 (0.03)	13 (0.02)	7 (0.02)	6 (0.02)	26 (0.01)
OR_cr_ [95% CI]	Ref. 1.0	0.62 [0.31, 1.25]	0.58 [0.25,1.37]	0.68 [0.27,1.68]	0.18 [0.10, 0.32]
OR_adj_ [95% CI]	Ref. 1.0	0.65 [0.32,1.30]	0.61 [0.26,1.43]	0.70 [0.28,1.74]	0.19 [0.11,0.35]

Odds ratios (OR) estimated using logistic regression. Crude (OR_cr_) and adjusted (OR_adj_) models compare vacuum-assisted delivery (VAD) and spontaneous vaginal delivery (SVD) with emergency cesarean delivery (ECD) as the reference group. Adjusted models control for maternal age, body-mass index (BMI), gestational age, pre-eclampsia, gestational diabetes, and diabetes mellitus type I/II.

Percentages are calculated within each delivery mode column.

Mid/low VAD was associated with markedly increased odds of traumatic intracerebral hemorrhage (adjusted odds ratio [aOR] 7.20, 95% CI [2.97, 17.45]), non-traumatic intracerebral hemorrhage (aOR 3.65, 95% CI [2.41, 5.52]), subgaleal hematoma (aOR 3.35, 95% CI [2.68, 4.20]), cephalohematoma (aOR 6.97, 95% CI [6.30, 7.70]), and neonatal seizures (aOR 1.85, 95% CI [1.52, 2.26]) compared with ECD.

Outlet VAD was also associated with increased odds, though to a lesser extent, for traumatic intracerebral hemorrhage (aOR 3.28, 95% CI [1.25, 8.61]) and cephalohematoma (aOR 6.18, 95% CI [5.60, 6.81]), but not for subgaleal hematoma (aOR 0.96, 95% CI [0.72, 1.27]) or neonatal seizures (aOR 0.87, 95% CI [0.69, 1.10]).

In contrast, both mid/low and outlet VAD were associated with lower odds of meconium aspiration (aOR 0.53, 95% CI [0.42, 0.67] and aOR 0.53, 95% CI [0.42, 0.65], respectively) compared with ECD. Outlet VAD was additionally associated with reduced odds of asphyxia (aOR 0.65, 95% CI [0.60, 0.71]) and respiratory distress (aOR 0.65, 95% CI [0.61, 0.70]), whereas mid/low VAD showed a modestly higher odds of asphyxia (aOR 1.18, 95% CI [1.10, 1.27]) and no significant association with respiratory distress (aOR 0.96, 95% CI [0.90, 1.03]). No significant association was observed between either VAD group and ischemic stroke.

### Long-term neurodevelopmental outcomes

Long-term outcomes are summarized in Table 4 and detailed in Table D in S1 Appendix. During a median follow-up of 13–14 years, children born by outlet VAD had a significantly lower risk of ADHD compared with those delivered by ECD (adjusted hazard ratio [aHR] 0.91, 95% CI [0.86, 0.95]), whereas no significant association was observed for mid/low VAD.

**Table 4:** Long-term neurodevelopmental outcomes in children by mode of delivery, expressed as incidence rates (IR) and hazard ratios (HR) with 95% confidence intervals, comparing vacuum-assisted delivery and spontaneous vaginal delivery with emergency cesarean delivery as reference.

		Vacuum-Assisted Delivery			
	Emergency cesarean Delivery	All	Outlet	Mid/Low	Spontaneous Vaginal Delivery
**Attention Deficit/Hyperactivity Disorder**					
**All**					
Follow-up, years	12.91 ± 5.29	12.99 ± 5.10	13.13 ± 5.15	12.85 ± 5.02	13.27 ± 5.44
Event, *n*	5,351	4,851	2,742	2,109	32 096
IR [95% CI]	5.43 [5.28–5.57]	4.96 [4.82-5.10]	4.82 [4.64-5.00]	5.16 [4.94-5.38]	4.84 [4.79-4.89]
HR_Cr_ [95% CI]	Ref. 1.0	0.91 [0.87,0.94]	0.88 [0.84,0.92]	0.94 [0.90,0.99]	0.89 [0.87,0.92]
HR_Adj_ [95% CI]	Ref. 1.0	0.94 [0.90-0.98]	0.91 [0.86-0.95]	0.99 [0.94-1.05]	0.88 [0.86-0.91]
**Boys**					
Follow-up, years	12.70 ± 5.30	12.84 ± 5.14	13.00 ± 5.18	12.68 ± 5.07	13.01 ± 5.47
Event [*n*]	3,753	3,451	1929	1,522	20 709
IR [95% CI]	6.80 [6.59,7.02]	6.36 [6.15,6.58]	6.16 [5.88,6.43]	6.64 [6.31,6.98]	6.38 [6.29,6.47]
HR_Cr_ [95% CI]	Ref. 1.0	0.92 [0.88-0.97]	0.90 [0.85-0.95]	0.96 [0.91-1.02]	0.94 [0.91-0.98]
HR_Adj_ [95% CI]	Ref. 1.0	0.96 [0.91-1.01]	0.93 [0.88-0.99]	1.00 [0.94-1.07]	0.93 [0.90-0.97]
**Girls**					
Follow-up, years	13.22 ± 5.26	13.18 ± 5.04	13.25 ± 5.10	13.07 ± 4.95	13.50 ± 5.40
Event, *n*	1,598	1,400	813	587	11 387
IR [95% CI]	3.68 [3.50-3.86]	3.22 [3.05-3.39]	3.19 [2.97-3.41]	3.26 [3.00-3.53]	3.36 [3.30-3.42]
HR_Cr_ [95% CI]	Ref. 1.0	0.87 [0.81,0.93]	0.86 [0.79,0.93]	0.89 [0.81,0.97]	0.91 [0.86,0.95]
HR_Adj_ [95% CI]	Ref. 1.0	0.92 [0.85-1.00]	0.89 [0.81-0.97]	0.97 [0.88-1.08]	0.91 [0.86-0.97]
**Autism Spectrum Disorder**					
**All**					
Follow-up, years	13.26 ± 5.32	13.32 ± 5.12	13.45 ± 5.17	13.12 ± 5.05	13.62 ± 5.46
Event, *n*	2,139	1875	1,055	820	11 293
IR [95% CI]	2.13 [2.04,2.23]	1.89 [1.81,1.98]	1.83 [1.72,1.94]	1.98 [1.84,2.11]	1.68 [1.64,1.71]
HR_Cr_ [95% CI]	Ref. 1.0	0.88 [0.83,0.93]	0.85 [0.79,0.92]	0.92 [0.85,0.99]	0.79 [0.75,0.82]
HR_Adj_ [95% CI]	Ref. 1.0	0.94 [0.88,1.00]	0.93 [0.85,1.00]	0.96 [0.87,1.04]	0.86 [0.82,0.90]
**Boys**					
Follow-up, years	13.14 ± 5.34	13.30 ± 5.16	13.48 ± 5.20	13.05 ± 5.11	13.53 ± 5.49
Event, *n*	1,567	1,373	758	615	7,657
IR [95% CI]	2.78 [2.65,2.92]	2.48 [2.35,2.6]	2.37 [2.20,2.54]	2.63 [2.42,2.84]	2.31 [2.26,2.36]
HR_Cr_ [95% CI]	Ref. 1.0	0.88 [0.82,0.95]	0.85 [0.78,0.92]	0.93 [0.85,1.03]	0.84 [0.79,0.88]
HR_Adj_ [95% CI]	Ref. 1.0	0.95 [0.88,1.02]	0.93 [0.85,1.02]	0.97 [0.87,1.07]	0.91 [0.85,0.96]
**Girls**					
Follow-up, years	13.43 ± 5.30	13.34 ± 5.06	13.42 ± 5.13	13.22 ± 4.97	13.71 ± 5.43
Event, *n*	572	502	297	205	3,636
IR [95% CI]	1.30 [1.20,1.41]	1.14 [1.04,1.24]	1.15 [1.02,1.29]	1.13 [0.98,1.28]	1.06 [1.03,1.10]
HR_Cr_ [95% CI]	Ref. 1.0	0.88 [0.78,0.99]	0.88 [0.77,1.01]	0.87 [0.74,1.02]	0.81 [0.74,0.89]
HR_Adj_ [95% CI]	Ref. 1.0	0.93 [0.82,1.06]	0.94 [0.81,1.10]	0.92 [0.77,1.10]	0.91 [0.83,1.00]
**Cerebral Palsy**					
**All**					
Follow-up, years	13.46 ± 5.30	13.48 ± 5.10	13.61 ± 5.15	13.29 ± 5.04	13.80 ± 5.44
Event, *n*	207	159	79	80	576
IR [95% CI]	0.20 [0.18,0.23]	0.16 [0.13,0.18]	0.14 [0.11,0.17]	0.19 [0,15,0.23]	0.08 [0.08,0.09]
HR_Cr_ [95% CI]	Ref. 1.0	0.77 [0.60,0.99]	0.70 [0.52,0.95]	0.86 [0.63,1.18]	0.50 [0.42,0.60]
HR_Adj_ [95% CI]	Ref. 1.0	0.83 [0.64,1.08]	0.78 [0.57,1.06]	0.91 [0.65,1.27]	0.52 [0.43,0.64]
**Boys**					
Follow-up, years	13.41 ± 5.29	13.52 ± 5.12	13.69 ± 5.15	13.29 ± 5.07	13.80 ± 5.45
Event, *n*	113	98	46	52	322
IR [95% CI]	0.20 [0.16,0.23]	0.17 [0.14,0.21]	0.14 [0.10,0.18]	0.22 [0.16,0.28]	0.10 [0.09,0.11]
HR_Cr_ [95% CI]	Ref. 1.0	0.90 [0.66,1.24]	0.77 [0.52,1.14]	1.09 [0.74,1.61]	0.54 [0.42,0.70]
HR_Adj_ [95% CI]	Ref. 1.0	0.98 [0.71,1.37]	0.85 [0.57,1.29]	1.16 [0.77,1.73]	0.56 [0.43,0.73]
**Girls**					
Follow-up, years	13.52 ± 5.32	13.41 ± 5.08	13.50 ± 5.14	13.30 ± 4.99	13.80 ± 5.43
Event, *n*	94	61	33	28	254
IR [95% CI]	0.21 [0.17,0.26]	0.14 [0.10,0.17]	0.13 [0.08,0.17]	0.15 [0.10,0.21]	0.07 [0.06,0.08]
HR_Cr_ [95% CI]	Ref. 1.0	0.60 [0.40,0.89]	0.62 [0.39,1.00]	0.57 [0.32,0.99]	0.46 [0.35,0.61]
HR_Adj_ [95% CI]	Ref. 1.0	0.64 [0.42, 0.97]	0.68 [0.41, 1.12]	0.58 [0.32, 1.06]	0.49 [0.36, 0.67]
**Epilepsy**					
**All**					
Follow-up, years	13.40 ± 5.33	13.43 ± 5.12	13.55 ± 5.17	13.24 ± 5.05	13.75 ± 5.47
Event, *n*	729	619	335	284	3,639
IR [95% CI]	0.72 [0.67,0.78]	0.62 [0.57,0.67]	0.58 [0.52,0.64]	0.68 [0.60,0.76]	0.54 [0.52,0.55]
HR_Cr_ [95% CI]	Ref. 1.0	0.86 [0.77, 0.96	0.80 [0.69, 0.92]	0.95 [0.82, 1.09]	0.78 [0.71, 0.84]
HR_Adj_ [95% CI]	Ref. 1.0	0.88 [0.78,1.00]	0.82 [0.71,0.95]	0.97 [0.83,1.14]	0.81 [0.74,0.89]
**Boys**					
Follow-up, years	13.34 ± 5.31	13.47 ± 5.14	13.64 ± 5.18	13.24 ± 5.09	13.74 ± 5.47
Event, *n*	406	390	201	189	1892
IR [95% CI]	0.71 [0.64,0.78]	0.70 [0.63,0.77]	0.62 [0.54,0.71]	0.80 [0.69,0.92]	0.56 [0.54,0.59]
HR_Cr_ [95% CI]	Ref. 1.0	0.99 [0.85,1.14]	0.87 [0.73,1.05]	1.13 [0.94,1.36]	0.83 [0.74,0.93]
HR_Adj_ [95% CI]	Ref. 1.0	1.03 [0.88,1.21]	0.93 [0.77,1.13]	1.17 [0.96,1.42]	0.87 [0.77,0.99]
**Girls**					
Follow-up, years	13.46 ± 5.34	13.38 ± 5.09	13.46 ± 5.15	13.26 ± 5.00	13.76 ± 5.46
Event, *n*	323	229	134	95	1,747
IR [95% CI]	0.74 [0.66,0.82]	0.52 [0.45,0.59]	0.52 [0.43,0.61]	0.52 [0.42,0.63]	0.51 [0.49,0.53]
HR_Cr_ [95% CI]	Ref. 1.0	0.71 [0.59,0.85]	0.70 [0.57,0.87]	0.71 [0.56,0.91]	0.72 [0.64,0.82]
HR_Adj_ [95% CI]	Ref. 1.0	0.70 [0.58,0.85]	0.68 [0.54,0.86]	0.73 [0.56,0.95]	0.74 [0.65,0.85]
**Intellectual Disability**					
**All**					
Follow-up, years	14.43 ± 5.26	14.46 ± 5.07	14.59 ± 5.13	14.29 ± 5.00	14.78 ± 5.43
Event, *n*	599	446	266	180	2,699
IR [95% CI]	0.55 [0.51,0.60	0.42 [0.38,0.45]	0.43 [0.38,0.48	0.40 [0.34,0.46]	0.37 [0.36,0.39]
HR_Cr_ [95% CI]	Ref. 1.0	0.75 [0.66, 0.84]	0.77 [0.67,0.89	0.72 [0.61,0.85]	0.68 [0.62, 0.74]
HR_Adj_ [95% CI]	Ref. 1.0	0.83 [0.73,0.95]	0.86 [0.74,1.00]	0.80 [0.67,0.96]	0.72 [0.65,0.80]
**Boys**					
Follow-up, years	14.37 ± 5.26	14.51 ± 5.09	14.67 ± 5.13	14.29 ± 5.03	14.76 ± 5.44
Event, *n*	391	279	167	112	1,697
IR [95% CI]	0.64 [0.58,0.70]	0.46 [0.41,0.52]	0.48 [0.41,0.56]	0.44 [0.36,0.52]	0.47 [0.45,0.49]
HR_Cr_ [95% CI]	Ref. 1.0	0.72 [0.62,0.84]	0.75 [0.63,0.90]	0.68 [0.55,0.84]	0.75 [0.67,0.83]
HR_Adj_ [95% CI]	Ref. 1.0	0.83 [0.70,0.98]	0.86 [0.71,1.04]	0.79 [0.63,0.99]	0.82 [0.72,0.93]
**Girls**					
Follow-up, years	14.51 ± 5.27	14.40 ± 5.05	14.49 ± 5.12	14.29 ± 4.96	14.79 ± 5.42
Event, *n*	208	167	99	68	1,002
IR [95% CI]	0.44 [0.38,0.50]	0.35 [0.30,0.41]	0.36 [0.29,0.43]	0.35 [0.27,0.43]	0.27 [0.26,0.29]
HR_Cr_ [95% CI]	Ref. 1.0	0.80 [0.65,0.98]	0.81 [0.64,1.03]	0.78 [0.59,1.03]	0.62 [0.54,0.73]
HR_Adj_ [95% CI]	Ref. 1.0	0.85 [0.68,1.06]	0.87 [0.67,1.13]	0.82 [0.61,1.11]	0.63 [0.54,0.75]

Data are incidence rates (IR) and hazard ratios (HR) with 95% CIs. HRs were estimated with Cox proportional hazards models. Crude (HR_cr_) and adjusted (HR_Adj_) models compare vacuum-assisted delivery (stratified by fetal head station: outlet, mid/low) and spontaneous vaginal delivery with emergency cesarean delivery as the reference category. Fully adjusted models included maternal age, body-mass index in early pregnancy, maternal smoking, child’s birth year, preeclampsia, gestational diabetes, chorioamnionitis, maternal education level, and maternal comorbidities, including diabetes mellitus type I or II, attention deficit/hyperactivity disorder, autism spectrum disorder, depression, and anxiety.

IR, incidence rate (per 1 000 person-years); CI, confidence interval.

The risk of ASD was slightly lower among children born by outlet VAD (aHR 0.93, 95% CI [0.85, 1.00]), with no difference for mid/low VAD. For intellectual disability (ID), both outlet and mid/low VAD were associated with lower risks compared with ECD (outlet VAD aHR 0.86, 95% CI [0.74, 1.00]; mid/low VAD aHR 0.80, 95% CI [0.67, 0.96]). Children delivered by outlet VAD also had a reduced risk of epilepsy (EP; aHR 0.82, 95% CI [0.71, 0.95]), while no association was seen for mid/low VAD. No significant differences were observed between either VAD category or ECD for cerebral palsy (CP).

Sex-stratified analyses yielded largely consistent findings, with no major differences between boys and girls across outcomes, except for a slightly lower risk of epilepsy among girls born by VAD. In general, children delivered by spontaneous vaginal delivery had substantially lower risks for all assessed neurodevelopmental outcomes compared with those delivered by ECD.

### Sensitivity analyses

Findings from sensitivity analyses (Tables D-T in the S1 Appendix) were largely consistent with the main results. The main deviation was observed among children born large for gestational age (LGA) and those born post-term, where risks of adverse long-term outcomes were generally similar across delivery modes, including spontaneous vaginal delivery. Among children born LGA, outlet VAD was associated with a notably lower risk of ADHD compared with ECD, aHR 0.45, 95% CI [0.30, 0.68].

## Discussion

Using nationwide data with long-term follow-up, we found no evidence that VAD was associated with increased risk of long-term neurodevelopmental disorders in offspring. Although short-term neonatal morbidity varied by fetal head station, with higher risks of some neonatal complications after mid/low VAD, these short-term risks did not appear to translate into increased long-term neurodevelopmental morbidity at the population level.

When assessing short-term outcomes, including intracranial hemorrhage and neonatal seizures, we found higher risks among children born via mid/low VAD compared with ECD, consistent with previous research [4–7,11,12,27–34]. Although odds for certain neonatal complications were increased after mid/low VAD, the absolute risks were low. For example, traumatic intracranial hemorrhage occurred in only a few cases per 10,000 births, underscoring that these events were rare despite elevated relative estimates. While severe intracranial hemorrhage following VAD is rare, it can result in long-lasting sequelae, while milder cases may go undetected [35,36]. Prior studies have linked subgaleal hemorrhage, which is more common after VAD, to an increased risk of EP, CP, auditory impairment, and thrombosis [37], and have associated symptomatic intracranial hemorrhage after VAD with adverse long-term outcomes [38]. Neonatal brain injury has also been implicated as a potential risk factor for later neuropsychiatric disorders [39]. Furthermore, complicated mid/low VAD procedures involving high traction forces have been associated with adverse neonatal outcomes [12], supporting a possible mechanistic link between mid/low VAD and subsequent neurodevelopmental disorders. Our findings reinforce concerns that mid/low VAD carries short-term risks which, in rare cases, may contribute to adverse neurodevelopmental trajectories. However, despite these short-term risks, our long-term follow-up showed no increase in neurodevelopmental disorders among children born by mid/low VAD compared with ECD. These results suggest that although mid/low VAD entails greater neonatal morbidity, these complications do not necessarily translate into long-term neurodevelopmental impairment.

Previous research has generally reported no adverse long-term outcomes associated with VAD. Seidman and colleagues found no differences in cognitive performance at age 17 in children born via VAD or forceps compared to spontaneous vaginal delivery [13]. Ayala and colleagues reported similar academic outcomes in third grade [18], and Ngan and colleagues observed no differences in motor skills or IQ at 10 years when comparing VAD and spontaneous vaginal delivery [15]. However, these studies did not compare VAD with ECD or account for fetal head station. Ahlberg and colleagues found lower school performance at 16 years for both VAD and ECD compared to spontaneous vaginal delivery, but no difference between VAD and ECD [16], and Ulfsdottir and colleagues similarly found no difference in the risk of CP or EP when comparing VAD and ECD [17]. Neither study, however, distinguished between outlet and mid/low station VAD.

When the analyses were stratified by gestational age, a broadly consistent pattern was observed across most categories. In post-term pregnancies, rates of long-term neurodevelopmental outcomes were similar across all delivery modes. The number of events was limited in the post-term subgroup, and these exploratory findings should therefore be interpreted with caution. Nevertheless, the absence of a clear protective pattern for spontaneous vaginal delivery in post-term births may be viewed as broadly compatible with current clinical strategies aimed at avoiding prolonged pregnancies [40–42].

Prior research has proposed that abnormal fetal growth may influence later neurodevelopment through epigenetic mechanisms involving aberrant DNA methylation and altered gene expression [43]. Although our study did not investigate underlying biological mechanisms, these hypotheses could guide future research.

Sex-stratified analyses confirmed higher incidence rates of ADHD, ASD, and ID among boys compared with girls, consistent with prior studies. Relative risks across delivery modes were generally similar between sexes, except for a slightly lower risk of EP among girls born by VAD. Diagnostic delay in girls, who are more often diagnosed with ADHD at a later age [44], may explain part of the observed difference.

This study’s key strengths include its large, nationally representative cohort, long follow-up, with virtually no loss of follow-up, and comprehensive linkage of real-world maternal, neonatal, and child health data. Distinguishing between outlet and mid/low procedures enabled a detailed assessment of neonatal and long-term neurodevelopmental outcomes after VAD.

Nevertheless, some limitations warrant consideration. Reverse causation remains possible, as fetal compromise may both prompt operative delivery and contribute to subsequent outcomes. However, sensitivity analyses excluding neonates with asphyxia yielded consistent findings.

Missing information on fetal head station resulted in exclusion of a subset of VADs. However, births with known and unknown station were comparable with respect to measured characteristics, suggesting limited risk of major selection bias (Table C in the S1 Appendix).

Information on indication for intervention, duration of second stage, and whether ECD was performed in the first or second stage was not available in the registers. Because VAD is, by definition, performed in the second stage, whereas ECD may occur earlier, the ECD reference group likely comprised a heterogenous mix of clinical situations. Second-stage cesarean deliveries are often technically more challenging and may involve an impacted fetal head, factors associated with increased neonatal morbidity. This heterogeneity should be considered when interpreting comparisons between delivery modes. Importantly, this study was not designed to determine the optimal mode of delivery in acute intrapartum situations. The clinical circumstances leading to VAD and ECD differ and are incompletely captured in register data; therefore, comparisons with ECD should be interpreted as contextual rather than as direct evidence to guide acute delivery-mode decision-making. Within this framework, our findings provide reassuring population-based evidence regarding the long-term neurodevelopmental safety of VAD when clinically indicated and appropriately performed, while confirming that mid/low VAD is associated with increased short-term neonatal morbidity.

While Swedish registers have high validity, regional differences in diagnostic coding could introduce information bias. Temporal changes in obstetric and neonatal care might also have influenced outcomes, although stratified analyses by birth cohort (1997–2006 versus ≥ 2,007) produced consistent results. The limited number of EP cases, particularly among girls, may reduce precision. Finally, as in all observational research, residual confounding cannot be excluded. Given Sweden’s universal health system, standardized obstetric practices, and comprehensive national data coverage, these findings are likely generalizable to other high-income settings with similar models of maternity care.

In conclusion, this nationwide population-based cohort study provides reassuring evidence regarding the long-term neurodevelopmental safety of VAD. Children born after mid/low VAD had increased risks of some short-term neonatal complications, but these did not translate into increased risks of ADHD, ASD, CP, epilepsy, or intellectual disability during long-term follow-up. Outlet VAD showed no signal of adverse long-term neurodevelopmental outcomes. These findings should be interpreted as a safety assessment of VAD rather than as evidence to guide acute choice between VAD and ECD.

From a global perspective, these results align with international efforts to strengthen the capacity for safe instrumental vaginal delivery as a strategy to reduce unnecessary cesarean deliveries and improve maternal and neonatal health. In settings where surgical services, anesthesia, and postoperative care are limited, expanding access to VAD performed by trained providers could help reduce preventable morbidity and mortality. Our findings provide contemporary evidence on the long-term safety of VAD and support continued emphasis on training, supervision, and system-level readiness for safe instrumental delivery.

### Ethics statement

Ethical approval for the study was obtained from the Swedish Ethical Review Authority (Ref. no. 2022-02513-01). Informed consent was waived because the study used pseudonymized registry data.

## Supporting information

S1 Checklist Completed STROBE checklist. Strengthening the Reporting of OBservational studies in Epidemiology (STROBE) Statement – checklist of items that should be included in reports of observational studies, licensed under CC BY 4.0, von Elm E, Altman DG, Egger M, Pocock SJ, Gøtzsche PC, Vandenbroucke JP; STROBE Initiative. The Strengthening the Reporting of Observational Studies in Epidemiology (STROBE) Statement: guidelines for reporting observational studies. PLoS Med. 2007;4(10):e296. https://doi.org/10.1371/journal.pmed.0040296.
(PDF)

S1 Protocol Prospective analysis plan developed before data analysis, including predefined outcomes, covariates, and statistical analyses. (PDF)

S1 Appendix Supplementary methods, definitions of exposures and outcomes, additional tables and figures. (DOCX)

## Abbreviations

ADHD: attention deficit/hyperactivity disorder
aOR: adjusted odds ratio
ASD: autism spectrum disorder
ATC: Anatomical Therapeutic Chemical
BMI: body mass index
CI: confidence interval
CIF: cumulative incidence function
CP: cerebral palsy
ECD: emergency cesarean delivery
EP: epilepsy
HRs: hazard ratios
ID: intellectual disability
ORs: odds ratios
PIN: personal identification number
STROBE: Strengthening the Reporting of Observational Studies in epidemiology
VAD: vacuum-assisted delivery
