# Supplementary material for: Long-term neurodevelopmental outcomes after vacuum-assisted delivery: A population-based cohort study
Source: PLoS Med. 2026 Jul 17;23(7):e1004825. doi: 10.1371/journal.pmed.1004825 (PMC13395438; doi:10.1371/journal.pmed.1004825)
Supplement: S1 Checklist — Strengthening the Reporting of OBservational studies in Epidemiology (STROBE) Statement – checklist of items that should be included in reports of observational studies, licensed under CC BY 4.0, von Elm E, Altman DG, Egger M, Pocock SJ, Gøtzsche PC, Vandenbroucke JP; STROBE Initiative. The Strengthening the Reporting of Observational Studies in Epidemiology (STROBE) Statement: guidelines for reporting observational studies. PLoS Med. 2007;4(10):e296. https://doi.org/10.1371/journal.pmed.0040296. (PDF) [file pmed.1004825.s001.pdf]

S1 Checklist. The STROBE checklist for cohort studies is provided below, with corresponding section and paragraph references to the main manuscript. **The checklist is reproduced from the STROBE Statement (https://doi.org/10.1371/journal.pmed.0040296) and is licensed under the Creative Commons Attribution 4.0 International (CC BY 4.0) license.**

Item No	Recommendation	Section and paragraph(s) in manuscript
1a	Indicate the study's design in the title or abstract	Title
1b	Provide informative abstract	Abstract
2	Background/rationale	Introduction, paragraphs 1–5
3	Objectives, hypotheses	Introduction, final paragraph
4	Study design	Methods, Study Design, Setting and Data Sources, paragraph 1
5	Setting, data sources	Methods, Study Design, Setting and Data Sources, paragraphs 1–3
6a	Cohort study—eligibility criteria, methods of selection, follow-up	Methods, Study Participants; Outcomes and Follow-up; Figure 1
7	Variables (outcomes, exposures, confounders)	Methods, Exposure; Comparison Cohorts; Outcomes and Follow-up; Statistical Analysis
8	Data sources/measurement	Methods, Study Design, Setting and Data Sources; Outcomes and Follow-up
9	Bias, misclassification/confounding	Methods, Statistical Analysis; Discussion, Limitations
10	Study size	Methods, Study Participants; Results, paragraph 1; Figure 1
11	Quantitative variables handling	Methods, Statistical Analysis; Tables 1–4 footnotes
12a	Statistical methods, confounding control	Methods, Statistical Analysis
12b	Subgroup and interaction analyses	Methods, Statistical Analysis
12c	Missing data	Methods, Statistical Analysis; Table 1
12e	Sensitivity analyses	Methods, Statistical Analysis
13a	Participants, numbers at each stage	Methods, Study Participants; Results, paragraph 1; Figure 1
14a	Descriptive data (characteristics, exposures)	Results, Study Participants and Baseline Characteristics; Table 1
14b	Missing data	Methods, Statistical Analysis; Table 1
14c	Follow-up time	Methods, Long-term Neurodevelopmental Outcomes; Table 4

Item No	Recommendation	Section and paragraph(s) in manuscript
15	Outcome data	Results; Tables 1–4; Supplementary Tables S3–S20
16a	Main results, unadjusted and adjusted	Results; Tables 3–4
16b	Category boundaries for categorized variables	Methods, Statistical Analysis; Table 1 footnotes
17	Other analyses (subgroups, sensitivity)	Methods, Statistical Analysis; Results, Sensitivity analyses; Supplementary Tables S4–S20
18	Key results	Discussion, paragraph 1
19	Limitations	Discussion, paragraphs 7–10
20	Interpretation	Discussion
21	Generalizability	Discussion, final paragraph
22	Funding	Funding
