# Supplementary material for: Long-term neurodevelopmental outcomes after vacuum-assisted delivery: A population-based cohort study
Source: PLoS Med. 2026 Jul 17;23(7):e1004825. doi: 10.1371/journal.pmed.1004825 (PMC13395438; doi:10.1371/journal.pmed.1004825)
Supplement: S1 Protocol — (PDF) [file pmed.1004825.s002.pdf]

Ida Björk, PhD student, Clintec, KI

Analysis Plan

The association of vacuum assisted delivery and offspring neurodevelopmental outcomes; a nationwide population-based study

1. **Objective and hypotheses**

1.1 Objective

Ensuring a safe delivery is of utmost importance, always pursued to minimize both short-term and eventual long-term risks for the infant. Nonetheless, second stage of delivery, characterized by full cervical dilation and the descent of the foetus through the birth canal, can be particularly challenging for the child, given the potential constraints on its resources during this phase. In cases where spontaneous labour is unsuccessful at this stage, whether due to foetal distress or dystocia, the available options are vacuum assisted delivery (VAD) or emergency caesarean delivery (ECD), the utilization of forceps in contemporary obstetrics within Sweden is infrequent (1) and is currently not an option.

In clinical practice, selecting the optimal method of delivery presents a challenge, as both mid/low VAD and ECD include advantages and disadvantages. Previous research indicates that ECD more often leads to maternal infection and neonatal respiratory morbidity than VAD (2), but also important is to keep in mind the long-term risks of repeated caesarean sections in the same woman (3, 4). Often, it is only afterward that the obstetrician obtains a presumed answer key regarding whether the chosen delivery method was a suitable choice, and only short-term outcome is presented.

VAD performed at outlet station is usually a brief procedure, with minimal risk of injury in term born infants. (5). However, studies indicate a higher risk of severe neonatal complications (intracranial haemorrhage, seizures, and death) associated with VAD performed at mid/low station compared to ECD (6-8).

Even though VAD is frequently used, in Sweden it accounts for 6-9% of all deliveries (1), there is a vast lack of extensive research on the long-term outcomes for offspring. Existing studies are not only scarce but also predominantly dated (9-11). Two more recent performed studies show no difference in long term outcome when VAD was compared to ECD, but outcome that was studied could be extended for more relevance (12, 13). Moreover, none of these studies distinguish between VAD performed at outlet station and at mid/low station and only one study compare VAD to ECD (13). However, a recent study, within this research group, uncovered an increased likelihood of neurodevelopmental impairment in children delivered at term via VAD or ECD during a prolonged second stage (14).

Severe intracranial hemorrhage is a rare outcome after VAD which can lead to neurological sequalae for the child, while a modest hemorrhage might go undetected during the neonatal period.

An area of investigation concerns the possibility of minor intracranial hemorrhages leading to neurological impairment in childhood. Prior research indicates that children with severe traumatic brain injury are at a heightened risk for ADHD (15). Additionally, a previous study demonstrated more severe perinatal outcomes associated with use of higher traction forces during VAD procedure than use of lower traction forces (16) and it is alluring to investigate whether mid/low VAD is associated with a higher incidence of neurodevelopmental disorders, based on the hypothesis that it could parallel the effects of traumatic brain injury by the possibility of minor intracranial hemorrhage caused by high traction force.

Hence, this population-based study aims to meet the need of more research in this field by utilizing nationwide registers to investigate the enduring risks for the child following outlet VAD and mid/low VAD in comparison to ECD. Therefore, the objective of our study is to investigate the association between VAD, overall and by fetal station, and risk of adverse neurodevelopmental outcomes in offspring compared to ECD and spontaneous vaginal delivery.

2. Notation and abbreviations

ADHD	Attention Deficit Hyperactivity Disorder
ASD	Autism Spectrum Disorder
CP	Cerebral Palsy
CD	Cesarean Delivery
ECD	Emergency Cesarean Delivery
EP	Epilepsy
HIE	Hypoxic Ischemic Encephalopathy
ID	Intellectual Disability
VAD	Vacuum Assisted Delivery

3. Study setting and data sources

3.1 Study design

This is a population-based cohort study evaluating the association between delivery mode and long-term neurodevelopment disorder in childhood combining Swedish health registers.

The public Swedish healthcare system is tax funded; maternal healthcare is free of charge and virtually all women give birth at hospital-based delivery wards. All Swedish residents are accredited to a unique personal identification number (PIN), at birth or immigration (17) and this PIN number was used to link data from mother and child and to identify the study base, exposure, outcomes and other covariates of interest. In this study, we utilized the PIN to link information from several national administrative and health registers together.

The study cohort was identified in The Swedish Medical Birth Register (MBR) which was established in 1973 and data is prospectively collected during pregnancy. It contains information on demographic data, reproductive history, risk factors, comorbidities, and complications during pregnancy, delivery, and the neonatal period. It includes over 98% of the births in Sweden (18).

The National Patient Register (NPR) contains data on inpatient and outpatient care and hold information on main and secondary diagnoses coded according to the *International Statistical Classification of Diseases and Related Health Problems, Tenth Revision* [*ICD-10* version 10 since 1997] (19).

The Prescribed Drug Register was established in 2005 and contains information on dispensed drugs from all Swedish pharmacies (20).

The Total Population Register contains information on all Swedish residents containing data of demographic information, such as sex, age, civil status (21).

The Cause of Death Register contains information on all deaths in Sweden since 1952 including cause(s) of death coded according to the International Statistical Classification of Diseases (ICD; version 10 since 1996) (22).

The Longitudinal Integration Database for Health Insurance and Labor Market Studies (LISA) is administrated by The Statistical Central Bureau (SCB) and contains detailed information of health insurance, parental leave insurance and unemployment insurance on an individual level (23).

The Swedish Multi-Generation Register covers individuals registered in Sweden any time since 1961 and born 1932 or later. The register contains connections to biological parents, siblings and offsprings and consists of data more than 9 million individuals (24).

3.2 Study population

3.2.1 Source population

All singleton children born at term gestation (≥37+0 weeks) to primiparous women were identified in MBR from 1997 to 2014 and followed until December 31, 2021, or until the occurrence of an event, migration, or death. The inclusion period was selected to ensure that participants were at least 7 years old by the end of follow-up, providing sufficient time to identify ADHD, ASD, or ID. Follow-up began at different ages depending on the condition: at 3 months for CP, EP, and ID; at 1 year for ASD; and at 3 years for ADHD. These starting points were chosen to minimize the risk of misdiagnosis, as they align with the earliest possible diagnostic criteria. For EP, this also excluded seizures during the neonatal period.

3.2.2 Exclusion criterias

Children delivered by forceps-assisted birth, breech presentations or those with major congenital malformations.

3.2.3 Exposed cohort

Vacuum assisted delivery, further stratified into outlet and mid/low. The Swedish classification defines one fetal station between outlet and high, mid/low (medelhög). The international classification mid station is defined as when the leading point of the fetal head is between 0 and +2 cm, the ischial spines defined as 0, and low is defined as when the leading point of the head is at station +2 cm or more, but not touching the pelvic floor, this defined as +5 cm.

3.2.4 Unexposed cohort

Emergency cesarean delivery

3.2.5 Extern reference group

Spontaneus vaginal delivery

4. Measurements and variables

4.1 Outcome efficacy variables

Variable	Definition	ICD-10/ATC codes	Start of follow up	End of follow up

ADHD	≥2 registered diagnosis in the patient register and/or dispensed prescription of ADHD-specific pharmacotherapy in the drug register.	F90, ATC N06BA01, N06BA02, N06BA04, N06BA09, N06BA12	3 years of age (not possible to have outcome earlier, exclude incorrect given diagnosis)	211231/outcome registrated/death/ migration, whichever comes first
ASD	≥2 registered diagnosis in the patient register.	F84.0, F84.1, F84.3, F84.5, F848, F849	1 years of age (not possible to have outcome earlier, exclude incorrect given diagnosis)	211231/outcome registrated/death/ migration, whichever comes first
CP	≥2 registered diagnosis in the patient register.	G80	3 months of age (not possible to have outcome earlier, exclude incorrect given diagnosis)	211231/outcome registrated/death/ migration, whichever comes first
EP	≥2 registered diagnosis in the patient register.	G40	3 months of age (separate from seizures in the neonatal period)	211231/outcome registrated/death/ migration,

				whichever comes first
ID	≥2 registered diagnosis in the patient register.	F70-F79	3 months of age (not possible to have outcome earlier, exclude incorrect given diagnosis)	211231/outcome registrated/death/ migration, whichever comes first

4.2 Other caracteristics

Composite major neurologic perinatal outcome

Intracerebral bleedning, traumatic	P10, P100, P101, P102, P103, P108, P109, P104
Intracerebral bleedning, non-traumatic	P52, P520, P521, P522, P524, P525, P526, P528, P529
Extra cerebral bleeding, traumatic	P120, P122
Asphyxia and HIE	P21 P210, P211, P211A, P211B, P219, P910, P913, P918, P919 P916
Meconium aspiration	P240
Respiratory distress	P22, P220, P221, P228, P229, P25, P250, P251, P252, P253, P258, P26, P260, P261, P268, P269, P280, P281, P282, P284, P285, P288, P289

Seizures	P90, P909A, P909B, P909C, P911, P912, P914, P915
Ischemic stroke	I63, I64

4.3 Confounders of interest long-term outcome

Step 1 adjustment

- Maternal age
- Smoking
- BMI
- DM I/II
- Birthyear

Step 2 adjustment

- PE
- GDM
- Chorioamnionitis

Step 3 adjustment

- Maternal education

- Maternal comorbidity (ADHD, ASD, depression, anxiety disorder)

All analyses stratified on child sex. Analyses presented in table all, girls, boys in all steps.

4.4 Confounders of interest short-term outcome

- PE
- DM I/II
- GDM
- BMI
- Gest week
- Maternal age

5 Statistical analyses

Baseline characteristics will be calculated as means and standard deviations for continuous variables and as frequencies and percentages for categorical variables.

In order to assess the relative risk of adverse perinatal outcomes in exposed vs. unexposed and external reference cohorts, Logistic regression will be used to calculate odds ratios (ORs) with 95% CI.

Incidence rates of neurodevelopmental outcomes will be calc by dividing the number of cases by the total person-years of observation, expressed per 1,000 person-years.

Cox proportional Hazards regression models will be used to calculate Hazard Ratios (HRs) with 95% CI for all neurodevlepmental outcomes, any and the separate outcomes. Chidlren born by VAD, stratified into outlet vs- mid/low VAD, will be compraed with children born via Emergency cesarean delivery. For external comparison, exposed (all VAD) and unexposed (ECD) will be compared with a cohort of children deliverd by spontaneous vaginal birth. All main analyses will be stratified by child sex.

Multivariate Cox adjusted for pre-specified confounders will be used. All analyses will first be conducted with the entire cohort and then stratified by sex (boys and girls). Confounder adjustment will be performed in three sequential steps. In Step 1, adjustments will be made for maternal age, smoking status, body mass index (BMI), diabetes mellitus type I/II, and year of birth. Step 2 include all Step 1 confounders, with additional adjustments for preeclampsia, gestational diabetes mellitus, and chorioamnionitis. Step 3 further expand the adjustments to include all Step 1 and Step 2 confounders, along with maternal education level and maternal comorbidities (including ADHD, autism spectrum disorder (ASD), depression, and anxiety disorder).

5.1 Sensitivity analyses

To assure the robustness of our analyses, The following pre-defined sensitivity analyses will be performed:

i) Exclude asphyxia.

ii) Stratify by gestational length (early term (37+0 weeks to 38+6 weeks), full term (39+0 weeks to 40+6 weeks), late term (41+0 weeks to 41+6 weeks), post term ≥42+0 weeks))

iii) Stratify by small for gestational age (SGA), accurate for gestational age (AGA) and large for gestational age (LGA)

iv) Stratifiy by time period 1997-2007 och >2007

Data analyses will be performed in SAS, statistical software, version 9.4 (SAS Institue Inc). In this study we do not have information of indication for intervention, sufficient information on duration of second stage of labor or if ECD was carried out in first or second stage of labor, which all othervise had been interesting to include information on and potential effects of.

6 References

1. Graviditetsregistret. Graviditetsregistrets Årsrapport 2021 2023 [Available from: https://www.medscinet.com/GR/uploads/hemsida/dokumentarkiv/GR%20Årsrapport %202021_3.0.pdf

2. Halscott TL, Reddy UM, Landy HJ, Ramsey PS, Iqbal SN, Huang CC, et al. Maternal and Neonatal Outcomes by Attempted Mode of Operative Delivery From a Low Station in the Second Stage of Labor. Obstet Gynecol. 2015;126(6):1265-72.

3. Ali H, Chandraharan E. Etiopathogenesis and risk factors for placental accreta spectrum disorders. Best Pract Res Clin Obstet Gynaecol. 2021;72:4-12.

4. Klahr R, Cheung K, Markovic ES, Naert M, Rebarber A, Fox NS. Maternal Morbidity with Repeated Cesarean Deliveries. Am J Perinatol. 2023;40(13):1431-6.

5. Muraca GM, Sabr Y, Lisonkova S, Skoll A, Brant R, Cundiff GW, et al. Morbidity and Mortality Associated With Forceps and Vacuum Delivery at Outlet, Low, and Midpelvic Station. J Obstet Gynaecol Can. 2019;41(3):327-37.
6. Muraca GM, Boutin A, Razaz N, Lisonkova S, John S, Ting JY, et al. Maternal and neonatal trauma following operative vaginal delivery. CMAJ. 2022;194(1):E1-E12.
7. Muraca GM, Skoll A, Lisonkova S, Sabr Y, Brant R, Cundiff GW, et al. Perinatal and maternal morbidity and mortality among term singletons following midcavity operative vaginal delivery versus caesarean delivery. BJOG. 2018;125(6):693-702.
8. Muraca GM, Sabr Y, Lisonkova S, Skoll A, Brant R, Cundiff GW, et al. Perinatal and maternal morbidity and mortality after attempted operative vaginal delivery at midpelvic station. CMAJ. 2017;189(22):E764-E72.
9. Seidman DS, Laor A, Gale R, Stevenson DK, Mashiach S, Danon YL. Long-term effects of vacuum and forceps deliveries. Lancet. 1991;337(8757):1583-5.
10. Johanson RB, Heycock E, Carter J, Sultan AH, Walklate K, Jones PW. Maternal and child health after assisted vaginal delivery: five-year follow up of a randomised controlled study comparing forceps and ventouse. Br J Obstet Gynaecol. 1999;106(6):544-9.
11. Ngan HY, Miu P, Ko L, Ma HK. Long-term neurological sequelae following vacuum extractor delivery. Aust N Z J Obstet Gynaecol. 1990;30(2):111-4.
12. Ahlberg M, Ekeus C, Hjern A. Birth by vacuum extraction delivery and school performance at 16 years of age. Am J Obstet Gynecol. 2014;210(4):361 e1- e8.

13. Ulfsdottir H, Ekeus C, Tedroff K, Aberg K, Jarnbert-Pettersson H. Long-term neurological morbidity among children delivered by vacuum extraction - a national cohort study. Acta Obstet Gynecol Scand. 2023;102(7):843-53.

14. Romero S. Traction force and long-term outcome in children born after vacuum-assisted delivery. [
]: Karolinska Institute; 2022.

15. Asarnow RF, Newman N, Weiss RE, Su E. Association of Attention-Deficit/Hyperactivity Disorder Diagnoses With Pediatric Traumatic Brain Injury: A Meta-analysis. JAMA Pediatr. 2021;175(10):1009-16.

16. Romero S, Pettersson K, Yousaf K, Westgren M, Ajne G. Traction force profile in children with severe perinatal outcomes delivered with a digital vacuum extraction handle: A case-control study. Acta Obstet Gynecol Scand. 2022;101(11):1238-44.

17. Ludvigsson JF, Otterblad-Olausson P, Pettersson BU, Ekbom A. The Swedish personal identity number: possibilities and pitfalls in healthcare and medical research. Eur J Epidemiol. 2009;24(11):659-67.

18. Cnattingius S, Kallen K, Sandstrom A, Rydberg H, Mansson H, Stephansson O, et al. The Swedish medical birth register during five decades: documentation of the content and quality of the register. Eur J Epidemiol. 2023;38(1):109-20.

19. Ludvigsson JF, Andersson E, Ekbom A, Feychting M, Kim JL, Reuterwall C, et al. External review and validation of the Swedish national inpatient register. BMC Public Health. 2011;11:450.

20. Wettermark B, Hammar N, Fored CM, Leimanis A, Otterblad Olausson P, Bergman U, et al. The new Swedish Prescribed Drug Register--opportunities for pharmacoepidemiological research and experience from the first six months. Pharmacoepidemiol Drug Saf. 2007;16(7):726-35.

21. Ludvigsson JF, Almqvist C, Bonamy AK, Ljung R, Michaelsson K, Neovius M, et al. Registers of the Swedish total population and their use in medical research. Eur J Epidemiol. 2016;31(2):125-36.

22. Brooke HL, Talback M, Hornblad J, Johansson LA, Ludvigsson JF, Druid H, et al. The Swedish cause of death register. Eur J Epidemiol. 2017;32(9):765-73.

23. SCB S. Longitudinell integrationsdatabas för sjukförsäkrings- och arbetsmarknadsstudier (LISA) 2023 [Available from: https://www.scb.se/vara-tjanster/bestall-data-och-statistik/bestalla-mikrodata/vilka-mikrodata-finns/longitudinella-register/longitudinell-integrationsdatabas-for-sjukforsakrings--och-arbetsmarknadsstudier-lisa/.

24. Ekbom A. The Swedish Multi-generation Register. Methods Mol Biol. 2011;675:215-20.
