# Supplementary material for: Long-term neurodevelopmental outcomes after vacuum-assisted delivery: A population-based cohort study
Source: PLoS Med. 2026 Jul 17;23(7):e1004825. doi: 10.1371/journal.pmed.1004825 (PMC13395438; doi:10.1371/journal.pmed.1004825)

**Supplementary Appendix**

**Long-term Neurodevelopmental Outcomes after Vacuum-Assisted Delivery: a Population-based Cohort Study**

Ida Björk et al.

**Contents**

[**Table A. ICD-10 and ATC-codes Used to identify Exposure, Outcomes and covariates** 3](#_Toc222047957)

[**Table B. Tests of proportional hazards** using exposure–time interaction terms 4](#_Toc222047958)

[**Table C. Baseline maternal characteristics by mode of delivery** among primiparous women, Sweden 1997-2014 5](#_Toc222047959)

[**Table D. Long-term neurodevelopmental outcomes in children by mode of delivery,** expressed as incidence rates (IR) and hazard ratios (HR) with 95% confidence intervals, comparing vacuum-assisted delivery and spontaneous vaginal delivery with emergency caesarean delivery as reference. 8](#_Toc222047960)

[**Table E. Long-term neurodevelopmental outcomes in children by mode of delivery,** expressed as incidence rates (IR) and hazard ratios (HR) with 95% confidence intervals, comparing vacuum-assisted delivery and spontaneous vaginal delivery with emergency caesarean delivery as reference. **Neonates with asphyxia excluded from analysis.** 10](#_Toc222047961)

[**Table F. Incidence and hazard ratios of attention-deficit/hyperactivity-disorder** in children by mode of delivery, comparing vacuum-assisted delivery and spontaneous vaginal delivery with emergency caesarean delivery as reference, **stratified by gestational age at delivery categories.** 12](#_Toc222047962)

[**Table G. Incidence and hazard ratios of autism spectrum disorder** in children by mode of delivery, comparing vacuum-assisted delivery and spontaneous vaginal delivery with emergency caesarean delivery as reference, **stratified by gestational age at delivery categories.** 13](#_Toc222047963)

[**Table H. Incidence and hazard ratios of cerebral palsy** in children by mode of delivery, comparing vacuum-assisted delivery and spontaneous vaginal delivery with emergency caesarean delivery as reference, **stratified by gestational age at delivery categories.** 15](#_Toc222047964)

[**Table I. Incidence and hazard ratios of epilepsy** in children by mode of delivery, comparing vacuum-assisted delivery and spontaneous vaginal delivery with emergency caesarean delivery as reference, **stratified by gestational age at delivery categories.** 16](#_Toc222047965)

[**Table J. Incidence and hazard ratios of intellectual disability** in children by mode of delivery, comparing vacuum-assisted delivery and spontaneous vaginal delivery with emergency caesarean delivery as reference, **stratified by gestational age at delivery categories.** 18](#_Toc222047966)

[**Table K. Incidence and hazard ratios of attention-deficit/hyperactivity disorder** in children by mode of delivery, comparing vacuum-assisted delivery and spontaneous vaginal delivery with emergency caesarean delivery as reference, **stratified by birth cohort (children born between 1997 and 2006 and 2007 or later).** 20](#_Toc222047967)

[**Table L. Incidence and hazard ratios of autism spectrum disorder** in children by mode of delivery, comparing vacuum-assisted delivery and spontaneous vaginal delivery with emergency caesarean delivery as reference, **stratified by birth cohort (children born between 1997 and 2006 and 2007 or later).** 21](#_Toc222047968)

[**Table M.** **Incidence and hazard ratios of cerebral palsy** in children by mode of delivery, comparing vacuum-assisted delivery and spontaneous vaginal delivery with emergency caesarean delivery as reference, **stratified by birth cohort (children born between 1997 and 2006 and 2007 or later).** 22](#_Toc222047969)

[**Table N. Incidence and hazard ratios of epilepsy** in children by mode of delivery, comparing vacuum-assisted delivery and spontaneous vaginal delivery with emergency caesarean delivery as reference, **stratified by birth cohort (children born between 1997 and 2006 and 2007 or later).** 23](#_Toc222047970)

[**Table O. Incidence and hazard ratios of intellectual disability** in children by mode of delivery, comparing vacuum-assisted delivery and spontaneous vaginal delivery with emergency caesarean delivery as reference, **stratified by birth cohort (children born between 1997 and 2006 and 2007 or later).** 24](#_Toc222047971)

[**Table P Incidence and hazard ratios of attention-deficit/hyperactivity disorder** in children by mode of delivery, comparing vacuum-assisted delivery and spontaneous vaginal delivery with emergency caesarean delivery as reference, **stratified by estimated relative fetal size.** 26](#_Toc222047972)

[**Table Q. Incidence and hazard ratios of autism spectrum disorder** in children by mode of delivery, comparing vacuum-assisted delivery and spontaneous vaginal delivery with emergency caesarean delivery as reference, **stratified by estimated relative fetal size.** 27](#_Toc222047973)

[**Table R. Incidence and hazard ratios of cerebral palsy** in children by mode of delivery, comparing vacuum-assisted delivery and spontaneous vaginal delivery with emergency caesarean delivery as reference, **stratified by estimated relative fetal size.** 29](#_Toc222047974)

[**Table S. Incidence and hazard ratios of epilepsy** in children by mode of delivery, comparing vacuum-assisted delivery and spontaneous vaginal delivery with emergency caesarean delivery as reference, **stratified by estimated relative fetal size.** 30](#_Toc222047975)

[**Table T. Incidence and hazard ratios of intellectual disability** in children by mode of delivery, comparing vacuum-assisted delivery and spontaneous vaginal delivery with emergency caesarean delivery as reference, **stratified by estimated relative fetal size.** 32](#_Toc222047976)

[**Figure A. Cumulative incidence functions (CIFs)** 34](#_Toc222047977)

##

## **Table A. ICD-10 and ATC-codes Used to identify Exposure, Outcomes and covariates**

| Condition | ICD 10, ATC-codes |
| --- | --- |
| Preeclampsia | O14-15 |
| Gestational Diabetes Mellitus | O244 |
| Diabetes Mellitus type I/II | E10-14 |
| Depression | F32-34, F38-39 |
| Anxiety Disorder | F40-41 |
| Asphyxia | P21, P91 |
| Intracerebral bleeding, traumatic | P10 |
| Intracerebral bleeding, non-traumatic | P52 |
| Cephalohematoma | P120 |
| Subgaleal hematoma | P122 |
| Meconium Aspiration | P240 |
| Respiratory Distress | P22, P25, P26, P28 |
| Neonatal Seizures | P90-91 |
| Ischemic stroke | I63 |
| Chorioamnionitis | O411, P027 |
| Attention Deficit/Hyperactivity Disorder | ICD-10 F90  ATC-codes: N06BA01, N06BA02, N06BA04, N06BA09, N06BA12 |
| Autism Spectrum Disorder | F84 |
| Cerebral Palsy | G80 |
| Epilepsy | G40 |
| Intellectual Disability | F70-F79 |

## **Table B. Tests of proportional hazards** using exposure–time interaction terms

| **Outcome** | **Comparison** | **Time interaction** | **p-value** |
| --- | --- | --- | --- |
| ADHD | Outlet VAD vs ECD | Log(time) | 0.33 |
|  | Outlet VAD vs ECD | Time (linear) | 0.26 |
|  | Mid/low VAD vs ECD | Log(time) | 0.06 |
|  | Mid/low VAD vs ECD | Time (linear) | 0.07 |
| ASD | Outlet VAD vs ECD | Log(time) | 0.26 |
|  | Outlet VAD vs ECD | Time (linear) | 0.46 |
|  | Mid/low VAD vs ECD | Log(time) | 0.95 |
|  | Mid/low VAD vs ECD | Time (linear) | 0.66 |
| ID | Outlet VAD vs ECD | Log(time) | 0.50 |
|  | Outlet VAD vs ECD | Time (linear) | 0.67 |
|  | Mid/low VAD vs ECD | Log(time) | 0.88 |
|  | Mid/low VAD vs ECD | Time (linear) | 0.65 |
| CP | Outlet VAD vs ECD | Log(time) | 0.40 |
|  | Outlet VAD vs ECD | Time (linear) | 0.54 |
|  | Mid/low VAD vs ECD | Log(time) | 0.42 |
|  | Mid/low VAD vs ECD | Time (linear) | 0.62 |
| EP | Outlet VAD vs ECD | Log(time) | 0.38 |
|  | Outlet VAD vs ECD | Time (linear) | 0.58 |
|  | Mid/low VAD vs ECD | Log(time) | 0.65 |
|  | Mid/low VAD vs ECD | Time (linear) | 0.59 |
| P-values derived from time-dependent interaction terms between exposure and follow-up time. | | | |

##

## **Table C. Baseline maternal characteristics by mode of delivery** among primiparous women, Sweden 1997-2014

|  |  | **Vacuum-Assisted Delivery** | | | |  |
| --- | --- | --- | --- | --- | --- | --- |
|  | **Emergency Cesarean Delivery**  **n 73 739** | **All**  **n 73 215** | **Outlet**  **n**  **42 201** | **Mid/Low**  **n 31 014** | **Unspecified station**  **n 13 486** | **Spontaneous Vaginal Delivery**  **n 484 031** |
| Maternal age years |  |  |  |  |  |  |
| Mean ± SD | 29.8 ± 5.1 | 29.1 ± 4.9 | 28.7 ± 4.9 | 29.6 ± 4.9 | 28.7±  4.8 | 27.6 ± 4.9 |
| n (%) |  |  |  |  |  |  |
| <18 | 237  (0.3) | 281  (0.4) | 196  (0.5) | 85  (0.3) | 61  (0.5) | 4360  (0.9) |
| 18-24 | 11 187  (15.2) | 12 913 (17.6) | 8258  (19.6) | 4655  (15.0) | 2542  (18.9) | 129 318  (26.7) |
| 25-29 | 18 158  (24.6) | 19 950 (27.3) | 11 955 (28.3) | 7995  (25.8) | 4046  (30.0) | 145 343  (30.0) |
| 30-34 | 26 886  (36.5) | 26 876 (36.7) | 15 005 (35.6) | 11 871  (38.3) | 4693  (34.8) | 150 773  (31.2) |
| 35-39 | 13 381  (18.2) | 10 834 (14.8) | 5604  (13.3) | 5230  (16.9) | 1776  (13.2) | 46 080  (9.5) |
| ≥40 | 3890  (5.3) | 2361  (3.2) | 1183  (2.8) | 1178  (3.8) | 368  (2.7) | 8157  (1.7) |
| BMI early pregnancy^†^ |  |  |  |  |  |  |
| Mean ± SD | 25.5 ±  4.9 | 23.8 ± 4.1 | 23.8 ± 4.0 | 24.1 ± 4.1 | 24.1±  4.1 | 23.8 ±  4.1 |
| n (%) |  |  |  |  |  |  |
| Underweight | 1009  (1.4) | 1910  (2.6) | 1261  (3.0) | 649  (2.1) | 299  (2.2) | 13 526  (2.8) |
| Normal weight | 35 450  (48.1) | 43 573 (59.5) | 25 570 (60.6) | 18 003  (58.1) | 7789  (57.8) | 292 714  (60.5) |
| Overweight | 16 682  (22.3) | 13 304 (18.1) | 7473  (17.7) | 5831  (18.8) | 2526  (18.7) | 83 101  (17.2) |
| Obesity, class 1 | 8701  (11.8) | 5094  (7.0) | 2779  (6.6) | 2315  (7.5) | 1009  (7.5) | 33 456  (6.9) |
| Obesity, >class 2 | 4206  (5.7) | 1893  (2.6) | 1035  (2.7) | 858  (2.8) | 342  (2,5) | 12 617  (2.6) |
| Missing | 7691  (10.4) | 7441  (10.2) | 4083  (9.7) | 3358  (10.8) | 1521  (11.3) | 48 617  (10.0) |
| Smoking early pregnancy^‡^ | 5255  (7.1) | 4677  (6.4) | 2869  (6.8) | 1808  (5.8) | 1082  (8.0) | 37 952  (7.8) |
| Missing | 3945  (5.4) | 3725  (5.1) | 1943  (4.6) | 1782  (8.4) | 665  (5.0) | 23 712  (4.9) |
|  |  |  |  |  |  |  |

| Gestational week of delivery |  |  |  |  |  |  |
| --- | --- | --- | --- | --- | --- | --- |
| 37+0-38+6 | 8664  (11.8) | 8527  (11.7) | 5207  (12.3) | 3320  (10.7) | 1503  (11.1) | 81 060  (16.8) |
| 39+0-40+6 | 29 025  (39.4) | 36 446  (49.8) | 21 370  (50.6) | 15 076  (48.6) | 6479  (48.0) | 268 452  (55.5) |
| 41+0-41+6 | 19 740  (26.8) | 18 804  (25.7) | 10 564  (25.0) | 8240  (26.6) | 3366  (25.0) | 96 961  (20.0) |
| ≥42+0 | 16 310  (22.1) | 9438  (12.9) | 5060  (12.0) | 4378  (14.1) | 1906  (14.1) | 37 558  (7.8) |
| Missing | 0 | 0 | 0 | 0 | 232  (1.7) | 0 |
| Adverse pregnancy outcomes |  |  |  |  |  |  |
| Pregnancy-induced hypertensive disorder^§^ | 5472 (7.4) | 2593  (3.5) | 1491  (3.5) | 1102  (3.6) | 569  (4.2) | 13 542  (2.8) |
| Gestational diabetes^§^ | 940  (1.3) | 567  (0.8) | 321  (0.8) | 246  (0.8) | 121  (0.9) | 3217  (0.7) |
| Pre-pregnancy comorbidities^¶^ |  |  |  |  |  |  |
| Diabetes mellitus type I/II^c^ | 762  (1.0) | 284  (0.4) | 151  (0.4) | 133  (0.4) | 51  (0.4) | 838  (0.2) |
| ADHD | 273  (0.4) | 195  (0.3) | 117  (0.3) | 78  (0.3) | 26  (0.2) | 1988  (0.4) |
| Autism spectrum disorder^c^ | 49  (0.1) | 53  (0.1) | 31  (0.1) | 22  (0.1) | 4  (0.0) | 429  (0.1) |
| Maternal depression^c^ | 2321  (3.2) | 1903  (2.6) | 1071  (2.5) | 832  (2.7) | 211  (1.6) | 13 067  (2.7) |
| Maternal anxiety disorder^c^ | 2134  (2.9) | 1773  (2.4) | 1004  (2.4) | 769  (2.5) | 188  (1.4) | 11 448  (2.4) |
| Alcohol and/or drug abuse^b^ | 1586  (2.2) | 1346  (1.8) | 762  (1.8) | 584  (1.9) | 206  (1.5) | 10 519  (2.2) |
| ^†^BMI categories classified according to WHO criteria: underweight <18.5 kg/m²; normal weight 18.5–24.9 kg/m²; overweight 25.0–29.9 kg/m²; obesity class I 30.0–34.9 kg/m²; and obesity class II or higher ≥35.0 kg/m²  ^‡^Smoking during early pregnancy as reported by the midwife at the first antenatal care visit, recorded in the Swedish Medical Birth Register  §Based on registered diagnoses according to ICD codes in the Swedish Medical Birth Register  ^¶^Based on registered diagnoses according to ICD codes in the National Patient Register before pregnancy  Percentages are calculated within each delivery mode column  ADHD, Attention-deficit/Hyperactivity disorder | | | | | | |

##

## **Table D. Long-term neurodevelopmental outcomes in children by mode of delivery,** expressed as incidence rates (IR) and hazard ratios (HR) with 95% confidence intervals, comparing vacuum-assisted delivery and spontaneous vaginal delivery with emergency caesarean delivery as reference.

|  | **Attention Deficit/Hyperactivity Disorder** | | | | | **Autism Spectrum Disorder** | | | | | **Cerebral Palsy** | | | | | **Epilepsy** | | | | | **Intellectual Disability** | | | | |
| --- | --- | --- | --- | --- | --- | --- | --- | --- | --- | --- | --- | --- | --- | --- | --- | --- | --- | --- | --- | --- | --- | --- | --- | --- | --- |
|  |  | Vacuum-Assisted delivery | | |  |  | Vacuum-Assisted delivery | | |  |  | Vacuum-Assisted delivery | | |  |  | Vacuum-Assisted delivery | | |  |  | Vacuum-Assisted delivery | | |  |
|  | ECD | All | Outlet | M/L | SVD | ECD | All | Outlet | M/L | SVD | ECD | All | Outlet | M/L | SVD | ECD | All | Outlet | M/L | SVD | ECD | All | Outlet | M/L | SVD |
| **All** | | | | | | | | | | | | | | | | | | | | | | | | | |
| Event, n | 5351 | 4851 | 2742 | 2109 | 32 096 | 2139 | 1875 | 1055 | 820 | 11 293 | 207 | 159 | 79 | 80 | 576 | 729 | 619 | 335 | 284 | 3639 | 599 | 446 | 266 | 180 | 2699 |
| IR  (95% CI) | 5.43 (5.28-5.57) | 4.96 (4.82-5.10) | 4.82 (4.64-5.00) | 5.16 (4.94-5.38) | 4.84 (4.79-4.89) | 2.13 (2.04-2.23) | 1.89 (1.81-1.98) | 1.83 (1.72-1.94) | 1.98 (1.84-2.11) | 1.68 (1.64-1.71) | 0.20 (0.18-0.23) | 0.16 (0.13-0.18) | 0.14 (0.11-0.17) | 0.19 (0-15-0.23) | 0.08 (0.08-0.09) | 0.72 (0.67-0.78) | 0.62 (0.57-0.67) | 0.58 (0.52-0.64) | 0.68 (0.60-0.76) | 0.54 (0.52-0.55) | 0.55 (0.51-0.60 | 0.42 (0.38-0.45) | 0.43 (0.38-0.48 | 0.40 (0.34-0.46) | 0.37 (0.36-0.39) |
| HR_Cr_ (95% CI) | Ref.  1.0 | 0.91 (0.87- 0.94) | 0.88 (0.84- 0.92) | 0.94 (0.90- 0.99) | 0.89 (0.87- 0.92) | Ref.  1.0 | 0.88 (0.83- 0.93) | 0.85 (0.79- 0.92) | 0.92 (0.85- 0.99) | 0.79 (0.75- 0.82) | Ref.  1.0 | 0.77 (0.60- 0.99) | 0.70 (0.52- 0.95) | 0.86 (0.63- 1.18) | 0.50 (0.42- 0.60) | Ref.  1.0 | 0.86 (0.77- 0.96 | 0.80 (0.69- 0.92) | 0.95 (0.82- 1.09) | 0.78 (0.71- 0.84) | Ref.  1.0 | 0.75 (0.66- 0.84) | 0.77 (0.67-0.89 | 0.72 (0.61-0.85) | 0.68 (0.62- 0.74) |
| HR_Adj1_ (95% CI) | Ref.  1.0 | 0.93 (0.89-0.97) | 0.89 (0.85- 0.94) | 0.98 (0.93- 1.03) | 0.87 (0.84- 0.90) | Ref.  1.0 | 0.92 (0.86- 0.99) | 0.91 (0.84- 0.99) | 0.94 (0.86- 1.03) | 0.84 (0.80- 0.89) | Ref.  1.0 | 0.80 (0.62-1.04) | 0.75 (0.54-1.02) | 0.88 (0.63-1.22) | 0.50 (0.41-0.61) | Ref.  1.0 | 0.88 (0.78-0.99) | 0.81 (0.70-0.94) | 0.97 (0.83-1.13) | 0.80 (0.73-0.88) | Ref.  1.0 | 0.80 (0.70-0.91) | 0.82 (0.70-0.96) | 0.76 (0.64-0.91) | 0.69 (0.62-0.76) |
| HR_Adj2_ (95% CI) | Ref.  1.0 | 0.93 (0.89-0.97) | 0.90 (0.85- 0.94) | 0.98 (0.93- 1.04) | 0.87 (0.84-0.90) | Ref.  1.0 | 0.93 (0.87- 0.99) | 0.92 (0.85- 0.99) | 0.95 (0.87- 1.03) | 0.85 (0.81- 0.89) | Ref.  1.0 | 0.82 (0.63-1.07) | 0.77 (0.56-1.05) | 0.90 (0.64-1.26) | 0.52 (0.42-0.64) | Ref.  1.0 | 0.88 (0.78-0.99) | 0.82 (0.70-0.95) | 0.97 (0.83-1.13) | 0.80 (0.73-0.88) | Ref.  1.0 | 0.81 (0.71-0.92) | 0.83 (0.71-0.97) | 0.77 (0.65-0.93) | 0.70 (0.63-0.77) |
| HR_Adj3_ (95% CI) | Ref.  1.0 | 0.94 (0.90-0.98) | 0.91 (0.86-0.95) | 0.99 (0.94-1.05) | 0.88 (0.86-0.91) | Ref.  1.0 | 0.94 (0.88- 1.00) | 0.93 (0.85- 1.00) | 0.96 (0.87- 1.04) | 0.86 (0.82- 0.90) | Ref.  1.0 | 0.83 (0.64-1.08) | 0.78 (0.57-1.06) | 0.91 (0.65-1.27) | 0.52 (0.43-0.64) | Ref.  1.0 | 0.88 (0.78-1.00) | 0.82 (0.71-0.95) | 0.97 (0.83-1.14) | 0.81 (0.74-0.89) | Ref.  1.0 | 0.83 (0.73-0.95) | 0.86 (0.74-1.00) | 0.80 (0.67-0.96) | 0.72 (0.65-0.80) |
| **Boys** | | | | | | | | | | | | | | | | | | | | | | | | | |
| Event, n | 3753 | 3451 | 1929 | 1522 | 20 709 | 1567 | 1373 | 758 | 615 | 7657 | 113 | 98 | 46 | 52 | 322 | 406 | 390 | 201 | 189 | 1892 | 391 | 279 | 167 | 112 | 1697 |
| IR  (95% CI) | 6.80 (6.59- 7.02) | 6.36 (6.15- 6.58) | 6.16 (5.88- 6.43) | 6.64 (6.31- 6.98) | 6.38 (6.29- 6.47) | 2.78 (2.65-2.92) | 2.48 (2.35-2.6) | 2.37 (2.20-2.54) | 2.63 (2.42-2.84) | 2.31 (2.26-2.36) | 0.20 (0.16-0.23) | 0.17 (0.14-0.21) | 0.14 (0.10-0.18) | 0.22 (0.16-0.28) | 0.10 (0.09-0.11) | 0.71 (0.64-0.78) | 0.70 (0.63-0.77) | 0.62 (0.54-0.71) | 0.80 (0.69-0.92) | 0.56 (0.54-0.59) | 0.64 (0.58-0.70) | 0.46 (0.41-0.52) | 0.48 (0.41-0.56) | 0.44 (0.36-0.52) | 0.47 (0.45-0.49) |
| HR_Cr_ (95% CI) | Ref.  1.0 | 0.92 (0.88-0.97) | 0.90 (0.85-0.95) | 0.96 (0.91-1.02) | 0.94 (0.91-0.98) | Ref.  1.0 | 0.88 (0.82- 0.95) | 0.85 (0.78- 0.92) | 0.93 (0.85- 1.03) | 0.84 (0.79- 0.88) | Ref.  1.0 | 0.90 (0.66- 1.24) | 0.77 (0.52-1.14) | 1.09 (0.74-1.61) | 0.54 (0.42-0.70) | Ref.  1.0 | 0.99 (0.85-1.14) | 0.87 (0.73-1.05) | 1.13 (0.94-1.36) | 0.83 (0.74-0.93) | Ref.  1.0 | 0.72 (0.62-0.84) | 0.75 (0.63-0.90) | 0.68 (0.55-0.84) | 0.75 (0.67-0.83) |
| HR_Adj1_ (95% CI) | Ref.  1.0 | 0.94 (0.90-0.99) | 0.91 (0.86-0.96) | 0.98 (0.92-1.05) | 0.91 (0.88-0.95) | Ref.  1.0 | 0.93 (0.86- 1.01) | 0.92 (0.84- 1.01) | 0.95 (0.86- 1.06) | 0.89 (0.84- 0.94) | Ref.  1.0 | 0.97 (0.70-1.35) | 0.82 (0.56-1.26) | 1.14 (0.76-1.71) | 0.55 (0.42-0.72) | Ref.  1.0 | 1.03 (0.88-1.20) | 0.93 (0.77-1.12) | 1.16 (0.95-1.41) | 0.86 (0.76-0.98) | Ref.  1.0 | 0.78 (0.66-0.92) | 0.81 (0.67-0.98) | 0.74 (0.59-0.93) | 0.77 (0.68-0.87) |
| HR_Adj2_ (95% CI) | Ref.  1.0 | 0.94 (0.90-0.99) | 0.91 (0.86-0.97) | 0.99 (0.93-1.05) | 0.92 (0.88-0.95) | Ref.  1.0 | 0.94 (0.87- 1.01) | 0.92 (0.84- 1.01) | 0.96 (0.87- 1.06) | 0.89 (0.84- 0.95) | Ref.  1.0 | 0.97 (0.70-1.35) | 0.84 (0.56-1.27) | 1.15 (0.76-1.72) | 0.55 (0.42-0.73) | Ref.  1.0 | 1.03 (0.88-1.20) | 0.93 (0.77-1.12) | 1.16 (0.95-1.41) | 0.86 (0.76-0.98) | Ref.  1.0 | 0.80 (0.68-0.94) | 0.83 (0.68-1.00) | 0.76 (0.60-0.95) | 0.78 (0.70-0.89) |
| HR_Adj3_ (95% CI) | Ref.  1.0 | 0.96 (0.91-1.01) | 0.93 (0.88-0.99) | 1.00 (0.94-1.07) | 0.93 (0.90-0.97) | Ref.  1.0 | 0.95 (0.88- 1.02) | 0.93 (0.85- 1.02) | 0.97 (0.87- 1.07) | 0.91 (0.85- 0.96) | Ref.  1.0 | 0.98 (0.71-1.37) | 0.85 (0.57-1.29) | 1.16 (0.77-1.73) | 0.56 (0.43-0.73) | Ref.  1.0 | 1.03 (0.88-1.21) | 0.93 (0.77-1.13) | 1.17 (0.96-1.42) | 0.87 (0.77-0.99) | Ref.  1.0 | 0.83 (0.70-0.98) | 0.86 (0.71-1.04) | 0.79 (0.63-0.99) | 0.82 (0.72-0.93) |
| **Girls** | | | | | | | | | | | | | | | | | | | | | | | | | |
| Event, n | 1598 | 1400 | 813 | 587 | 11 387 | 572 | 502 | 297 | 205 | 3636 | 94 | 61 | 33 | 28 | 254 | 323 | 229 | 134 | 95 | 1747 | 208 | 167 | 99 | 68 | 1002 |
| IR  (95% CI) | 3.68 (3.50-3.86) | 3.22 (3.05-3.39) | 3.19 (2.97-3.41) | 3.26 (3.00-3.53) | 3.36 (3.30-3.42) | 1.30 (1.20-1.41) | 1.14 (1.04-1.24) | 1.15 (1.02-1.29) | 1.13 (0.98-1.28) | 1.06 (1.03-1.10) | 0.21 (0.17-0.26) | 0.14 (0.10-0.17) | 0.13 (0.08-0.17) | 0.15 (0.10-0.21) | 0.07 (0.06-0.08) | 0.74 (0.66-0.82) | 0.52 (0.45-0.59) | 0.52 (0.43-0.61) | 0.52 (0.42-0.63) | 0.51 (0.49-0.53) | 0.44 (0.38-0.50) | 0.35 (0.30-0.41) | 0.36 (0.29-0.43) | 0.35 (0.27-0.43) | 0.27 (0.26-0.29) |
| HR_Cr_ (95% CI) | Ref.  1.0 | 0.87 (0.81- 0.93) | 0.86 (0.79- 0.93) | 0.89 (0.81- 0.97) | 0.91 (0.86- 0.95) | Ref.  1.0 | 0.88 (0.78-0.99) | 0.88 (0.77-1.01) | 0.87 (0.74-1.02) | 0.81 (0.74-0.89) | Ref.  1.0 | 0.60 (0.40-0.89) | 0.62 (0.39-1.00) | 0.57 (0.32-0.99) | 0.46 (0.35-0.61) | Ref.  1.0 | 0.71 (0.59-0.85) | 0.70 (0.57-0.87) | 0.71 (0.56-0.91) | 0.72 (0.64-0.82) | Ref.  1.0 | 0.80 (0.65-0.98) | 0.81 (0.64-1.03) | 0.78 (0.59-1.03) | 0.62 (0.54-0.73) |
| HR_Adj1_ (95% CI) | Ref.  1.0 | 0.91 (0.84- 0.99) | 0.88 (0.80-0.96) | 0.96 (0.87-1.07) | 0.90 (0.85- 0.96) | Ref.  1.0 | 0.91 (0.80-1.04) | 0.92 (0.79-1.07) | 0.90 (0.76-1.07) | 0.89 (0.81-0.98) | Ref.  1.0 | 0.60 (0.39-0.91) | 0.63 (0.39- 1.04) | 0.55 (0.30- 1.00) | 0.46 (0.34-0.62) | Ref.  1.0 | 0.70 (0.57-0.85) | 0.68 (0.54-0.86) | 0.72 (0.56-0.94) | 0.74 (0.64-0.85) | Ref.  1.0 | 0.83 (0.66-1.03) | 0.84 (0.65-1.09) | 0.80 (0.59-1.08) | 0.61 (0.52-0.72) |
| HR_Adj2_ (95% CI) | Ref.  1.0 | 0.91 (0.84-0.99) | 0.88 (0.80-0.96) | 0.96 (0.87-1.07) | 0.90 (0.85-0.96) | Ref.  1.0 | 0.93 (0.81-1.06) | 0.93 (0.80-1.09) | 0.92 (0.77-1.09) | 0.91 (0.82-1.00) | Ref.  1.0 | 0.63 (0.41- 0.97) | 0.67 (0.41- 1.11) | 0.58 (0.32- 1.06) | 0.49 (0.36- 0.66) | Ref.  1.0 | 0.70 (0.57-0.85) | 0.68 (0.54-0.86) | 0.73 (0.56-0.94) | 0.74 (0.65-0.85) | Ref.  1.0 | 0.83 (0.67-1.04) | 0.85 (0.66-1.10) | 0.81 (0.60-1.09) | 0.62 (0.53-0.73) |
| HR_Adj3_ (95% CI) | Ref.  1.0 | 0.92 (0.85-1.00) | 0.89 (0.81-0.97) | 0.97 (0.88-1.08) | 0.91 (0.86-0.97) | Ref.  1.0 | 0.93 (0.82-1.06) | 0.94 (0.81-1.10) | 0.92 (0.77-1.10) | 0.91 (0.83-1.00) | Ref.  1.0 | 0.64 (0.42- 0.97) | 0.68 (0.41- 1.12) | 0.58 (0.32- 1.06) | 0.49 (0.36- 0.67) | Ref.  1.0 | 0.70 (0.58-0.85) | 0.68 (0.54-0.86) | 0.73 (0.56-0.95) | 0.74 (0.65-0.85) | Ref.  1.0 | 0.85 (0.68-1.06) | 0.87 (0.67-1.13) | 0.82 (0.61-1.11) | 0.63 (0.54-0.75) |
| HRs were estimated with Cox proportional hazards models. Crude (HR_cr_) and adjusted (HR_Adj_) models compare vacuum-assisted delivery (stratified by fetal head station: outlet, mid/low) and spontaneous vaginal delivery with emergency caesarean delivery as the reference category. HR_Adj1_: Adjusted for maternal age, smoking, maternal BMI, diabetes mellitus type 1 or 2, birthyear. HR_Adj2_: Adjusted for variables in model one and gestational diabetes, preeclampsia and chorioamnionitis. HR_Adj3_: Adjusted for variables of model 1 and 2 and additionally for maternal educational level and maternal pre-pregnancy comorbidities (ADHD, ASD, depression, anxiety disorder).  CI = confidence interval; ECD, Emergency cesarean delivery; IR = incidence rate (per 1 000 person-years); M/L, Mid/Low; SVD, Spontaneous vaginal delivery; VAD, Vacuum-assisted delivery | | | | | | | | | | | | | | | | | | | | | | | | | |

## **Table E. Long-term neurodevelopmental outcomes in children by mode of delivery,** expressed as incidence rates (IR) and hazard ratios (HR) with 95% confidence intervals, comparing vacuum-assisted delivery and spontaneous vaginal delivery with emergency caesarean delivery as reference. **Neonates with asphyxia excluded from analysis.**

|  | **Attention Deficit/Hyperactivity Disorder** | | | | | **Autism Spectrum Disorder** | | | | | **Cerebral Palsy** | | | | | **Epilepsy (EP)** | | | | | **Intellectual Disability** | | | | |
| --- | --- | --- | --- | --- | --- | --- | --- | --- | --- | --- | --- | --- | --- | --- | --- | --- | --- | --- | --- | --- | --- | --- | --- | --- | --- |
|  |  | Vacuum-Assisted delivery | | |  |  | Vacuum-Assisted delivery | | |  |  | Vacuum-Assisted delivery | | |  |  | Vacuum-Assisted delivery | | |  |  | Vacuum-Assisted delivery | | |  |
|  | **ECD** | **All** | **Outlet** | **M/L** | **SVD** | **ECD** | **All** | **Outlet** | **M/L** | **SVD** | **ECD** | **All** | **Outlet** | **M/L** | **SVD** | **ECD** | **All** | **Outlet** | **M/L** | **SVD** | **ECD** | **All** | **Outlet** | **M/L** | **SVD** |
| **All** | | | | | | | | | | | | | | | | | | | | | | | | | |
| Event, n | 5145 | 4662 | 2671 | 1991 | 31 925 | 2036 | 1800 | 1024 | 776 | 11 204 | 132 | 110 | 63 | 47 | 506 | 640 | 566 | 312 | 254 | 3569 | 536 | 415 | 249 | 166 | 2633 |
| IR  (95% CI) | 5.42 (5.27-5.57) | 4.92 (4.78-5.06) | 4.81 (4.63-4.99) | 5.08 (4.85-5.30) | 4.84 (4.79-4.89) | 2.11 (2.02-2.20) | 1.87 (1.79-1.96) | 1.82 (1.71-1.93) | 1.95 (1.81-2.09) | 1.67 (1.64-1.70) | 0.14 (0.11-0.16) | 0.11 (0.09-0.13) | 0.11 (0.08-0.14) | 0.12 (0.08-0.15) | 0.07 (0.07-0.08) | 0.66 (0.61-0.71) | 0.59 (0.54-0.63) | 0.55 (0.49-0.61) | 0.63 (0.56-0.71) | 0.53 (0.51-0.55) | 0.51 (0.47-0.56) | 0.40 (0.36-0.44) | 0.41 (0.36-0.46) | 0.39 (0.33-0.44) | 0.36 (0.35-0.38) |
| HR_Cr_ (95% CI) | Ref.  1.0 | 0.90 (0.86- 0.93) | 0.88 (0.84- 0.92) | 0.93 (0.88- 0.98) | 0.89 (0.87- 0.92) | Ref.  1.0 | 0.88 (0.83-0.94) | 0.85 (0.79-0.92) | 0.92 (0.84-0.99) | 0.79 (0.76-0.83) | Ref.  1.0 | 0.77 (0.58-1.03) | 0.80 (0.57-1.13) | 0.73 (0.49-1.08) | 0.60 (0.48-0.74) | Ref.  1.0 | 0.88 (0.78-1.00) | 0.83 (0.72-0.96) | 0.96 (0.83-1.12) | 0.83 (0.76-0.91) | Ref.  1.0 | 0.77 (0.68-0.88) | 0.79 (0.68-0.92) | 0.74 (0.62-0.88) | 0.72 (0.65-0.79) |
| HR_Adj1_ (95% CI) | Ref.  1.0 | 0.92 (0.88-0.96) | 0.89 (0.85- 0.94) | 0.97 (0.91- 1.02) | 0.87 (0.84-0.90) | Ref.  1.0 | 0.92 (0.86-0.99) | 0.91 (0.84-0.99) | 0.94 (0.86-1.03) | 0.85 (0.80-0.89) | Ref.  1.0 | 0.78 (0.58-1.06) | 0.84 (0.59-1.19) | 0.71 (0.46-1.07) | 0.58 (0.47-0.73) | Ref.  1.0 | 0.90 (0.79-1.02) | 0.84 (0.72-0.98) | 0.98 (0.83-1.16) | 0.86 (0.78-0.95) | Ref.  1.0 | 0.83 (0.72-0.95) | 0.85 (0.72-0.99) | 0.80 (0.67-0.97) | 0.73 (0.66-0.80) |
| HR_Adj2_ (95% CI) | Ref.  1.0 | 0.92 (0.89-0.96) | 0.89 (0.85- 0.94) | 0.97 (0.92- 1.03) | 0.87 (0.84-0.90) | Ref.  1.0 | 0.93 (0.87-1.00) | 0.92 (0.85-0.99) | 0.95 (0.86-1.04) | 0.85 (0.81-0.90) | Ref.  1.0 | 0.80 (0.59-1.09) | 0.86 (0.60-1.22) | 0.72 (0.47-1.10) | 0.60 (0.48-0.75) | Ref.  1.0 | 0.90 (0.79-1.02) | 0.84 (0.72-0.98) | 0.98 (0.83-1.16) | 0.86 (0.78-0.95) | Ref.  1.0 | 0.84 (0.73-0.96) | 0.86 (0.73-1.01) | 0.81 (0.67-0.98) | 0.74 (0.67-0.82) |
| HR_Adj3_ (95% CI) | Ref.  1.0 | 0.94 (0.90-0.98) | 0.91 (0.86-0.95) | 0.98 (0.93-1.04) | 0.88 (0.86-0.91) | Ref.  1.0 | 0.94 (0.87-1.00) | 0.93 (0.85-1.00) | 0.95 (0.87-1.04) | 0.86 (0.82-0.91) | Ref.  1.0 | 0.81 (0.60-1.10) | 0.87 (0.61-1.23) | 0.73 (0.48-1.11) | 0.61 (0.48-0.76) | Ref.  1.0 | 0.90 (0.79-1.03) | 0.85 (0.72-0.99) | 0.99 (0.84-1.16) | 0.86 (0.78-0.95) | Ref.  1.0 | 0.87 (0.75-1.00) | 0.89 (0.75-1.04) | 0.84 (0.70-1.01) | 0.76 (0.70-0.85) |
| **Boys** | | | | | | | | | | | | | | | | | | | | | | | | | |
| Event, n | 3614 | 3313 | 1880 | 1433 | 20 596 | 1502 | 1316 | 736 | 580 | 7599 | 70 | 68 | 36 | 32 | 281 | 360 | 354 | 185 | 169 | 1849 | 361 | 258 | 155 | 103 | 1659 |
| IR  (95% CI) | 6.82 (6.60-7.04) | 6.31 (6.10-6.53) | 6.15 (5.87-6.43) | 6.54 (6.20-6.88) | 6.38 (6.30-6.47) | 2.78 (2.64-2.92) | 2.46 (2.32-2.59) | 2.36 (2.19-2.53) | 2.59 (2.39-2.81) | 2.30 (2.25-2.36) | 0.13 (0.10-0.16) | 0.13 (0.10-0.16) | 0.11 (0.08-0.15) | 0.14 (0.09-0.19) | 0.08 (0.07-0.09) | 0.66 (0.59-0.73) | 0.66 (0.59-0.72) | 0.59 (0.50-0.67) | 0.75 (0.63-0.86) | 0.56 (0.53-0.58) | 0.61 (0.55-0.68) | 0.44 (0.39-0.50) | 0.46 (0.39-0.53) | 0.42 (0.34-0.51) | 0.46 (0.44-0.49) |
| HR_Cr_ (95% CI) | Ref.  1.0 | 0.92 (0.87-0.96) | 0.89 (0.84-0.94) | 0.95 (0.89-1.00) | 0.94 (0.91-0.97) | Ref.  1.0 | 0.88 (0.82-0.95) | 0.84 (0.77-0.92) | 0.92 (0.84-1.02) | 0.84 (0.79-0.88) | Ref.  1.0 | 0.90 (0.62-1.29) | 0.86 (0.56-1.33) | 0.94 (0.59-1.51) | 0.62 (0.47-0.82) | Ref.  1.0 | 0.99 (0.85-1.16) | 0.88 (0.73-1.06) | 1.14 (0.94-1.38) | 0.88 (0.78-0.99) | Ref.  1.0 | 0.72 (0.61-0.84) | 0.75 (0.62-0.90) | 0.68 (0.55-0.85) | 0.77 (0.68-0.86) |
| HR_Adj1_ (95% CI) | Ref.  1.0 | 0.93 (0.89-0.98) | 0.91 (0.85-0.96) | 0.97 (0.91-1.04) | 0.91 (0.88-0.95) | Ref.  1.0 | 0.93 (0.86-1.00) | 0.91 (0.83-1.00) | 0.95 (0.85-1.05) | 0.89 (0.84-0.94) | Ref.  1.0 | 0.94 (0.64-1.37) | 0.92 (0.58-1.44) | 0.97 (0.59-1.59) | 0.62 (0.46-0.84) | Ref.  1.0 | 1.03 (0.87-1.22) | 0.94 (0.77-1.15) | 1.16 (0.94-1.43) | 0.91 (0.80-1.04) | Ref.  1.0 | 0.79 (0.66-0.94) | 0.81 (0.66-0.99) | 0.76 (0.60-0.96) | 0.79 (0.70-0.90) |
| HR_Adj2_ (95% CI) | Ref.  1.0 | 0.94 (0.89-0.98) | 0.91 (0.86-0.97) | 0.97 (0.91-1.04) | 0.92 (0.88-0.95) | Ref.  1.0 | 0.93 (0.86-1.01) | 0.92 (0.83-1.01) | 0.95 (0.86-1.06) | 0.89 (0.84-0.95) | Ref.  1.0 | 0.95 (0.65-1.40) | 0.93 (0.59-1.46) | 0.98 (0.60-1.61) | 0.63 (0.46-0.85) | Ref.  1.0 | 1.03 (0.87-1.22) | 0.94 (0.77-1.14) | 1.16 (0.94-1.43) | 0.91 (0.80-1.04) | Ref.  1.0 | 0.80 (0.68-0.95) | 0.82 (0.67-1.01) | 0.77 (0.61-0.98) | 0.81 (0.71-0.92) |
| HR_Adj3_ (95% CI) | Ref.  1.0 | 0.95 (0.90-1.00) | 0.93 (0.87-0.98) | 0.99 (0.92-1.06) | 0.93 (0.90-0.97) | Ref.  1.0 | 0.94 (0.87-1.02) | 0.93 (0.84-1.02) | 0.96 (0.87-1.07) | 0.90 (0.85-0.96) | Ref.  1.0 | 0.97 (0.66-1.42) | 0.95 (0.60-1.49) | 1.00 (0.61-1.63) | 0.64 (0.47-0.87) | Ref.  1.0 | 1.04 (0.88-1.23) | 0.94 (0.77-1.15) | 1.17 (0.95-1.44) | 0.92 (0.81-1.05) | Ref.  1.0 | 0.83 (0.70-0.99) | 0.85 (0.70-1.05) | 0.80 (0.64-1.02) | 0.84 (0.74-0.96) |
| **Girls** | | | | | | | | | | | | | | | | | | | | | | | | | |
| Event, n | 1531 | 1349 | 791 | 558 | 11 329 | 534 | 484 | 288 | 196 | 3605 | 62 | 42 | 27 | 15 | 225 | 280 | 212 | 127 | 85 | 1720 | 175 | 157 | 94 | 63 | 974 |
| IR  (95% CI) | 3.65 (3.47-3.84) | 3.19 (3.02-3.36) | 3.17 (2.94-3.39) | 3.23 (2.96-3.49) | 3.36 (3.30-3.42) | 1.26 (1.15-1.37) | 1.14 (1.03-1.24) | 1.14 (1.01-1.28) | 1.12 (0.97-1.28) | 1.06 (1.02-1.09) | 0.15 (0.11-0.18) | 0.10 (0.07-0.13) | 0.11 (0.07-0.15) | 0.09 (0.04-0.13) | 0.07 (0.06 (0.07) | 0.66 (0.58-0.74) | 0.50 (0.43-0.56) | 0.50 (0.42-0.59) | 0.49 (0.38-0.59) | 0.50 (0.48-0.53) | 0.38 (0.33-0.44) | 0.34 (0.29-0.40) | 0.35 (0.28-0.42) | 0.34 (0.25-0.42) | 0.27 (0.25-0.28) |
| HR_Cr_ (95% CI) | Ref.  1.0 | 0.87 (0.81-0.93) | 0.86 (0.79-0.94) | 0.88 (0.80-0.97) | 0.91 (0.86-0.96) | Ref.  1.0 | 0.90 (0.79-1.01) | 0.90 (0.78-1.04) | 0.89 (0.76-1.05) | 0.83 (0.76-0.91) | Ref.  1.0 | 0.60 (0.37-0.97) | 0.72 (0.42-1.24) | 0.43 (0.20-0.92) | 0.58 (0.42-0.80) | Ref.  1.0 | 0.75 (0.62-0.91) | 0.76 (0.61-0.95) | 0.74 (0.57-0.96) | 0.79 (0.69-0.90) | Ref.  1.0 | 0.88 (0.71-1.09) | 0.90 (0.70-1.15) | 0.86 (0.65-1.15) | 0.70 (0.60-0.82) |
| HR_Adj1_ (95% CI) | Ref.  1.0 | 0.91 (0.84-0.99) | 0.88 (0.80-0.97) | 0.96 (0.87-1.07) | 0.91 (0.86-0.96) | Ref.  1.0 | 0.93 (0.81-1.07) | 0.94 (0.80-1.10) | 0.92 (0.77-1.10) | 0.91 (0.82-1.01) | Ref.  1.0 | 0.58 (0.35-0.96) | 0.74 (0.42-1.28 | 0.35 (0.15-0.83) | 0.55 (0.39-0.78) | Ref.  1.0 | 0.73 (0.60-0.90) | 0.72 (0.56-0.92) | 0.75 (0.57-0.99) | 0.81 (0.70-0.93) | Ref.  1.0 | 0.92 (0.73-1.16) | 0.93 (0.71-1.22) | 0.89 (0.65-1.22) | 0.70 (0.58-0.82) |
| HR_Adj2_ (95% CI) | Ref.  1.0 | 0.91 (0.84-0.99) | 0.88 (0.80-0.97) | 0.96 (0.87-1.07) | 0.91 (0.86-0.96) | Ref.  1.0 | 0.95 (0.83-1.08) | 0.95 (0.81-1.11) | 0.93 (0.78-1.12) | 0.93 (0.84-1.02) | Ref.  1.0 | 0.60 (0.36-1.00) | 0.76 (0.44-1.33) | 0.37 (0.16-0.86) | 0.58 (0.41-0.82) | Ref.  1.0 | 0.73 (0.60-0.90) | 0.72 (0.57-0.92) | 0.75 (0.57-0.99) | 0.81 (0.70-0.94) | Ref.  1.0 | 0.92 (0.73-1.16) | 0.94 (0.72-1.23) | 0.90 (0.66-1.23) | 0.69 (0.58-0.83) |
| HR_Adj3_ (95% CI) | Ref.  1.0 | 0.92 (0.85-1.00) | 0.89 (0.81-0.98) | 0.97 (0.87-1.08) | 0.92 (0.87-0.98) | Ref.  1.0 | 0.95 (0.83-1.09) | 0.96 (0.82-1.12) | 0.94 (0.79-1.13) | 0.93 (0.84-1.03) | Ref.  1.0 | 0.61 (0.37-1.00) | 0.77 (0.44-1.34) | 0.37 (0.16-0.87) | 0.58 (0.41-0.82) | Ref.  1.0 | 0.74 (0.60-0.90) | 0.72 (0.57-0.93) | 0.75 (0.57-0.99) | 0.81 (0.70-0.94) | Ref.  1.0 | 0.94 (0.75-1.19) | 0.96 (0.73-1.26) | 0.91 (0.67-1.25) | 0.71 (0.59-0.85) |
| HRs were estimated with Cox proportional hazards models. Crude (HR_cr_) and adjusted (HR_Adj_) models compare vacuum-assisted delivery (stratified by fetal head station: outlet, mid/low) and spontaneous vaginal delivery with emergency caesarean delivery as the reference category. HR_Adj1_: Adjusted for maternal age, smoking, maternal BMI, diabetes mellitus type 1 or 2, birthyear. HR_Adj2_: Adjusted for variables in model one and gestational diabetes, preeclampsia and chorioamnionitis. HR_Adj3_: Adjusted for variables of model 1 and 2 and additionally for maternal educational level and maternal pre-pregnancy comorbidities (ADHD, ASD, depression, anxiety disorder).  CI = confidence interval; ECD, Emergency cesarean delivery; IR = incidence rate (per 1 000 person-years); M/L, Mid/Low; SVD, Spontaneous vaginal delivery; VAD, Vacuum-assisted delivery | | | | | | | | | | | | | | | | | | | | | | | | | |

## **Table F. Incidence and hazard ratios of attention-deficit/hyperactivity-disorder** in children by mode of delivery, comparing vacuum-assisted delivery and spontaneous vaginal delivery with emergency caesarean delivery as reference, **stratified by gestational age at delivery categories.**

|  | **Early term (37+0-38+6)** | | | | | **Full term (39+0-40+6)** | | | | | **Late term (41+0-41+6)** | | | | | **Post term (≥42+0)** | | | | |
| --- | --- | --- | --- | --- | --- | --- | --- | --- | --- | --- | --- | --- | --- | --- | --- | --- | --- | --- | --- | --- |
|  |  | **Vacuum-assisted delivery** | | |  |  | **Vacuum-assisted delivery** | | |  |  | **Vacuum-assisted delivery** | | |  |  | **Vacuum-assisted delivery** | | |  |
|  | **ECD** | **Total** | **Outlet** | **Mid/Low** | **SVD** | **ECD** | **Total** | **Outlet** | **Mid/Low** | **SVD** | **ECD** | **Total** | **Outlet** | **Mid/Low** | **SVD** | **ECD** | **Total** | **Outlet** | **Mid/low** | **SVD** |
| **All** | | | | | | | | | | | | | | | | | | | | |
| Event, n | 703 | 602 | 360 | 242 | 5628 | 2149 | 2403 | 1393 | 1010 | 17 484 | 1372 | 1207 | 642 | 565 | 6397 | 1127 | 639 | 347 | 292 | 2587 |
| IR (95% CI) | 6.07 (5.62-6.52) | 5.32 (4.90-5.75) | 5.18 (4.64-5.71) | 5.55 (4.85-6.24) | 5.04 (4.91-5.17) | 5.56 (5.33- 5.80) | 4.95 (4.75- 5.14) | 4.84 (4.59- 5.10) | 5.10 (4.78- 5.41) | 4.77 (4.70- 4.84) | 5.22 (4.95-5.50) | 4.80 (4.53-5.08) | 4.50 (4.15-4.85) | 5.20 (4.77-5.63) | 4.84 (4.72-4.96) | 5.09 (4.79- 5.39) | 5.02 (4.63- 5.41) | 5.05 (4.52- 5.58) | 4.98 (4.41- 5.55) | 4.92 (4.73- 5.11) |
| HR_Cr_ (95% CI) | Ref  1.0 | 0.87 (0.78- 0.96) | 0.84 (0.74- 0.96) | 0.90 (0.78- 1.04) | 0.83 (0.76- 0.89) | Ref  1.0 | 0.88 (0.83- 0.93) | 0.86 (0.81- 0.92) | 0.91 (0.84- 0.98) | 0.86 (0.82- 0.89) | Ref  1.0 | 0.91 (0.84- 0.98) | 0.85 (0.77- 0.93) | 0.98 (0.89- 1.08) | 0.92 (0.87- 0.98) | Ref  1.0 | 0.98 (0.89- 1.08) | 0.98 (0.87- 1.11) | 0.97 (0.86- 1.11) | 0.96 (0.90- 1.03) |
| HR_Adj1_ (95% CI) | Ref  1.0 | 0.93 (0.83-1.04) | 0.89 (0.78-1.02) | 0.99 (0.85-1.16) | 0.84 (0.77-0.91) | Ref  1.0 | 0.88 (0.83- 0.94) | 0.85 (0.79- 0.92) | 0.93 (0.85- 1.00) | 0.82 (0.78- 0.86) | Ref  1.0 | 0.93 (0.85-1.01) | 0.87 (0.79- 0.97) | 1.00 (0.90- 1.11) | 0.90 (0.85- 0.96) | Ref  1.0 | 1.05 (0.95- 1.16) | 1.03 (0.91- 1.18) | 1.07 (0.93- 1.23) | 0.96 (0.89- 1.04) |
| HR_Adj2_ (95% CI) | Ref  1.0 | 0.94 (0.84-1.06) | 0.90 (0.79-1.03) | 1.01 (0.86-1.18) | 0.85 (0.78-0.93) | Ref  1.0 | 0.88 (0.83- 0.94) | 0.86 (0.80- 0.92) | 0.93 (0.86- 1.01) | 0.83 (0.79- 0.87) | Ref  1.0 | 0.93 (0.85-1.01) | 0.87 (0.79-0.97) | 1.00 (0.90- 1.11) | 0.90 (0.85- 0.96) | Ref  1.0 | 1.05 (0.94- 1.16) | 1.03 (0.91- 1.17) | 1.07 (0.93- 1.22) | 0.96 (0.89- 1.03) |
| HR_Adj3_ (95% CI) | Ref  1.0 | 0.96 (0.85-1.08) | 0.92 (0.80-1.05) | 1.02 (0.88-1.20) | 0.87 (0.80-0.95) | Ref  1.0 | 0.90 (0.84- 0.96) | 0.87 (0.81- 0.93) | 0.94 (0.87- 1.02) | 0.84 (0.80- 0.89) | Ref  1.0 | 0.94 (0.87-1.03) | 0.89 (0.81-0.99) | 1.02 (0.91-1.13) | 0.91 (0.86-0.98) | Ref  1.0 | 1.06 (0.95- 1.17) | 1.04 (0.92- 1.18) | 1.07 (0.94- 1.23) | 0.96 (0.89- 1.04) |
| **Boys** | | | | | | | | | | | | | | | | | | | | |
| Event,  n | 482 | 422 | 247 | 175 | 3648 | 1493 | 1689 | 975 | 714 | 11 089 | 961 | 862 | 459 | 403 | 4238 | 817 | 478 | 248 | 230 | 1734 |
| IR (95% CI) | 7.99 (7.28-8.71) | 6.81 (6.16-7.45) | 6.46 (5.66-7.27) | 7.36 (6.27-8.45) | 6.63 (6.42-6.85) | 7.13 (6.77-7.49) | 6.43 (6.13-6.74) | 6.31 (5.91-6.70) | 6.62 (6.13-7.10) | 6.33 (6.21-6.45) | 6.51 (6.10-6.92) | 6.07 (5.66-6.47) | 5.73 (5.21-6.26) | 6.50 (5.86-7.13) | 6.41 (6.21-6.60) | 6.08 (5.66- 6.49) | 6.31 (5.75- 6.88) | 6.15 (5.38- 6.92) | 6.50 (5.66- 7.34) | 6.16 (5.87- 6.45) |
| HR_Cr_ (95% CI) | Ref  1.0 | 0.84 (0.74-0.96) | 0.80 (0.68-0.93) | 0.91 (0.76-1.08) | 0.83 (0.76-0.91) | Ref  1.0 | 0.89 (0.83- 0.95) | 0.87 (0.81- 0.95) | 0.91 (0.84-1.00) | 0.89 (0.84- 0.94) | Ref  1.0 | 0.92 (0.84-1.01) | 0.87 (0.78-0.97) | 0.99 (0.88-1.11) | 0.99 (0.92-1.06) | Ref  1.0 | 1.03 (0.92- 1.16) | 1.01 (0.87- 1.16) | 1.06 (0.92- 1.23) | 1.02 (0.94- 1.11) |
| HR_Adj1_ (95% CI) | Ref  1.0 | 0.91 (0.79-1.05) | 0.86 (0.73-1.01) | 1.01 (0.84-1.22) | 0.84 (0.76-0.93) | Ref  1.0 | 0.89 (0.82- 0.96) | 0.87 (0.80-0.95) | 0.92 (0.83-1.01) | 0.86 (0.81- 0.91) | Ref  1.0 | 0.93 (0.84-1.03) | 0.89 (0.79-1.00) | 0.98 (0.86-1.11) | 0.95 (0.88-1.02) | Ref  1.0 | 1.08 (0.96- 1.22) | 1.05 (0.90- 1.22) | 1.13 (0.96- 1.32) | 1.01 (0.92- 1.10) |
| HR_Adj2_ (95% CI) | Ref  1.0 | 0.93 (0.81-1.07) | 0.87 (0.74-1.03) | 1.03 (0.85-1.24) | 0.86 (0.77-0.96) | Ref  1.0 | 0.89 (0.83- 0.96) | 0.87 (0.80-0.95) | 0.92 (0.84-1.01) | 0.87 (0.82- 0.92) | Ref  1.0 | 0.93 (0.84-1.03) | 0.89 (0.79-1.01) | 0.98 (0.86-1.11) | 0.95 (0.88-1.03) | Ref  1.0 | 1.08 (0.96- 1.22) | 1.04 (0.90- 1.21) | 1.12 (0.96- 1.31) | 1.00 (0.91- 1.10) |
| HR_Adj3_ (95% CI) | Ref  1.0 | 0.95 (0.82-1.09) | 0.89 (0.76-1.05) | 1.04 (0.87-1.25) | 0.88 (0.79-0.98) | Ref  1.0 | 0.91 (0.84- 0.98) | 0.89 (0.81-0.97) | 0.94 (0.85-1.03) | 0.89 (0.84- 0.94) | Ref  1.0 | 0.95 (0.86-1.05) | 0.91 (0.81-1.03) | 1.00 (0.88-1.14) | 0.97 (0.89-1.04) | Ref  1.0 | 1.09 (0.97- 1.23) | 1.06 (0.91- 1.23) | 1.13 (0.97- 1.33) | 1.01 (0.92- 1.11) |
| **Girls** | | | | | | | | | | | | | | | | | | | | |
| Event,  n | 221 | 180 | 113 | 67 | 1980 | 656 | 714 | 418 | 296 | 6395 | 411 | 345 | 183 | 162 | 2159 | 310 | 161 | 99 | 62 | 853 |
| IR (95% CI) | 3.99 (3.46-4.51) | 3.52 (3.01-4.04) | 3.61 (2.95-4.28) | 3.38 (2.57-4.18) | 3.50 (3.34-3.65) | 3.70 (3.42-3.99) | 3.20 (2.96-3.43) | 3.14 (2.84-3.44) | 3.28 (2.91-3.65) | 3.34 (3.26-3.42) | 3.57 (3.23-3.92) | 3.16 (2.83-3.49) | 2.93 (2.50-3.35) | 3.47 (2.94-4.01) | 3.27 (3.13-3.41) | 3.56 (3.17- 3.96) | 3.12 (2.64-3.60) | 3.49 (2.80- 4.17) | 2.67 (2.01-3.34) | 3.49 (3.26-3.72) |
| HR_Cr_ (95% CI) | Ref  1.0 | 0.88 (0.73-1.08) | 0.90 (0.72-1.13) | 0.85 (0.65-1.12) | 0.87 (0.76-1.00) | Ref  1.0 | 0.86 (0.77-0.95) | 0.84 (0.74-0.95) | 0.88 (0.77-1.01) | 0.89 (0.82-0.97) | Ref  1.0 | 0.87 (0.76-1.01) | 0.81 (0.68-0.96) | 0.96 (0.80-1.15) | 0.90 (0.81-1.00) | Ref  1.0 | 0.87 (0.72- 1.05) | 0.96 (0.76- 1.20) | 0.76 (0.58-1.00) | 0.96 (0.84- 1.09) |
| HR_Adj1_ (95% CI) | Ref  1.0 | 0.93 (0.75-1.15) | 0.92 (0.72-1.18) | 0.93 (0.69-1.24) | 0.90 (0.77-1.05) | Ref  1.0 | 0.88 (0.79-0.99) | 0.85 (0.74-0.97) | 0.94 (0.81-1.09) | 0.87 (0.79-0.95) | Ref  1.0 | 0.92 (0.79-1.07) | 0.83 (0.69-1.01) | 1.04 (0.85-1.26) | 0.92 (0.82-1.03) | Ref  1.0 | 0.98 (0.80- 1.21) | 1.03 (0.81- 1.32) | 0.92 (0.69- 1.23) | 0.98 (0.85- 1.13) |
| HR_Adj2_ (95% CI) | Ref  1.0 | 0.93 (0.75-1.16) | 0.94 (0.73-1.20) | 0.93 (0.69-1.25) | 0.91 (0.78-1.07) | Ref  1.0 | 0.88 (0.78-0.99) | 0.84 (0.74-0.96) | 0.94 (0.81-1.09) | 0.86 (0.79-0.94) | Ref  1.0 | 0.92 (0.78-1.07) | 0.83 (0.69-1.01) | 1.03 (0.85-1.26) | 0.91 (0.81-1.03) | Ref  1.0 | 0.99 (0.80- 1.21) | 1.03 (0.81- 1.32) | 0.93 (0.70- 1.23) | 0.98 (0.85- 1.13) |
| HR_Adj3_ (95% CI) | Ref  1.0 | 0.95 (0.77-1.18) | 0.95 (0.74-1.22) | 0.95 (0.71-1.28) | 0.94 (0.80-1.09) | Ref  1.0 | 0.89 (0.79-0.99) | 0.85 (0.74-0.97) | 0.95 (0.81-1.10) | 0.88 (0.80-0.96) | Ref  1.0 | 0.93 (0.79-1.09) | 0.85 (0.70-1.02) | 1.04 (0.85-1.27) | 0.92 (0.82-1.04) | Ref  1.0 | 0.99 (0.80- 1.21) | 1.03 (0.80- 1.32) | 0.93 (0.70- 1.24) | 0.98 (0.85- 1.14) |
| HRs were estimated with Cox proportional hazards models. Crude (HR_cr_) and adjusted (HR_Adj_) models compare vacuum-assisted delivery (stratified by fetal head station: outlet, mid/low) and spontaneous vaginal delivery with emergency caesarean delivery as the reference category. HR_Adj1_: Adjusted for maternal age, smoking, maternal BMI, diabetes mellitus type 1 or 2, birthyear. HR_Adj2_: Adjusted for variables in model one and gestational diabetes, preeclampsia and chorioamnionitis. HR_Adj3_: Adjusted for variables of model 1 and 2 and additionally for maternal educational level and maternal pre-pregnancy comorbidities (ADHD, ASD, depression, anxiety disorder).  CI = confidence interval; ECD, Emergency cesarean delivery; IR = incidence rate (per 1 000 person-years); SVD, Spontaneous vaginal delivery | | | | | | | | | | | | | | | | | | | | |

## **Table G. Incidence and hazard ratios of autism spectrum disorder** in children by mode of delivery, comparing vacuum-assisted delivery and spontaneous vaginal delivery with emergency caesarean delivery as reference, **stratified by gestational age at delivery categories.**

|  | **Early term (37+0-38+6)** | | | | | **Full term (39+0-40+6)** | | | | | **Late term (41+0-41+6)** | | | | | **Post term (≥42+0)** | | | | |
| --- | --- | --- | --- | --- | --- | --- | --- | --- | --- | --- | --- | --- | --- | --- | --- | --- | --- | --- | --- | --- |
|  |  | **Vacuum-assisted delivery** | | |  |  | **Vacuum-assisted delivery** | | |  |  | **Vacuum-assisted delivery** | | |  |  | **Vacuum-assisted delivery** | | |  |
|  | **ECD** | **All** | **Outlet** | **Mid/Low** | **SVD** | **ECD** | **All** | **Outlet** | **Mid/Low** | **SVD** | **ECD** | **All** | **Outlet** | **Mid/Low** | **SVD** | **ECD** | **All** | **Outlet** | **Mid/low** | **SVD** |
| **All** | | | | | | | | | | | | | | | | | | | | |
| Event, n | 287 | 223 | 135 | 88 | 2035 | 830 | 905 | 527 | 378 | 6044 | 539 | 490 | 273 | 217 | 2212 | 483 | 257 | 120 | 137 | 1002 |
| IR (95% CI) | 2.44 (2.15- 2.72) | 1.94 (1.69- 2.20) | 1.91 (1.59- 2.24) | 1.99 (1.57- 2.40) | 1.79 (1.72- 1.87) | 2.11 (1.97- 2.25) | 1.84 (1.72- 1.96) | 1.81 (1.65- 1.96) | 1.88 (1.69- 2.07) | 1.62 (1.58- 1.66) | 2.02 (1.85- 2.19) | 1.92 (1.75- 2.09) | 1.89 (1.67- 2.12) | 1.97 (1.71- 2.23) | 1.65 (1.58- 1.72) | 2.15 (1.96- 2.34) | 1.99 (1.75- 2.23) | 1.72 (1.41- 2.03) | 2.31 (1.92- 2.69) | 1.87 (1.76- 1.99) |
| HR_Cr_ (95% CI) | Ref  1.0 | 0.79 (0.66- 0.94) | 0.78 (0.63- 0.95) | 0.81 (0.63- 1.02) | 0.74 (0.65- 0.83) | Ref  1.0 | 0.86 (0.79- 0.95) | 0.85 (0.76- 0.95) | 0.88 (0.78- 1.00) | 0.77 (0.72- 0.83) | Ref  1.0 | 0.94 (0.83- 1.06) | 0.93 (0.80- 1.07) | 0.96 (0.82- 1.13) | 0.82 (0.74- 0.90) | Ref  1.0 | 0.92 (0.79- 1.07) | 0.79 (0.65- 0.97) | 1.06 (0.88- 1.29) | 0.87 (0.78- 0.97) |
| HR_Adj1_ (95% CI) | Ref  1.0 | 0.87 (0.72- 1.05) | 0.86 (0.69- 1.07) | 0.89 (0.69- 1.15) | 0.82 (0.72- 0.95) | Ref  1.0 | 0.90 (0.81- 0.99) | 0.91 (0.81- 1.02) | 0.88 (0.77- 1.01) | 0.82 (0.76- 0.88) | Ref  1.0 | 0.96 (0.84- 1.10) | 0.96 (0.82- 1.12) | 0.97 (0.82- 1.15) | 0.85 (0.76- 0.94) | Ref  1.0 | 0.98 (0.83- 1.16) | 0.86 (0.69- 1.07) | 1.12 (0.91- 1.38) | 0.96 (0.85- 1.08) |
| HR_Adj2_ (95% CI) | Ref  1.0 | 0.88 (0.73- 1.07) | 0.87 (0.70- 1.09) | 0.90 (0.70- 1.17) | 0.84 (0.73- 0.97) | Ref  1.0 | 0.91 (0.82- 1.01) | 0.92 (0.82- 1.03) | 0.89 (0.78- 1.02) | 0.83 (0.77- 0.90) | Ref  1.0 | 0.97 (0.85- 1.10) | 0.96 (0.82- 1.13) | 0.97 (0.82- 1.16) | 0.85 (0.77- 0.94) | Ref  1.0 | 0.98 (0.83- 1.15) | 0.86 (0.69- 1.06) | 1.12 (0.91- 1.38) | 0.95 (0.84- 1.07) |
| HR_Adj3_ (95% CI) | Ref  1.0 | 0.90 (0.74- 1.08) | 0.89 (0.71- 1.11) | 0.91 (0.70- 1.18) | 0.86 (0.74- 0.99) | Ref  1.0 | 0.92 (0.83- 1.02) | 0.93 (0.83- 1.04) | 0.90 (0.79- 1.03) | 0.84 (0.78- 0.91) | Ref  1.0 | 0.97 (0.85- 1.11) | 0.97 (0.83- 1.13) | 0.98 (0.82- 1.16) | 0.86 (0.77- 0.95) | Ref  1.0 | 0.99 (0.84- 1.16) | 0.87 (0.70- 1.07) | 1.13 (0.92- 1.39) | 0.96 (0.85- 1.08) |

| **Boys** | | | | | | | | | | | | | | | | | | | | |
| --- | --- | --- | --- | --- | --- | --- | --- | --- | --- | --- | --- | --- | --- | --- | --- | --- | --- | --- | --- | --- |
| Event, n | 208 | 164 | 102 | 62 | 1374 | 603 | 676 | 378 | 298 | 4040 | 392 | 353 | 200 | 153 | 1557 | 364 | 180 | 78 | 102 | 686 |
| IR (95% CI) | 3.36 (2.91- 3.82) | 2.59 (2.20- 2.99) | 2.62 (2.11- 3.12) | 2.55 (1.92- 3.19) | 2.44 (2.31- 2.57) | 2.82 (2.59- 3.04) | 2.53 (2.33- 2.72) | 2.40 (2.16- 2.64) | 2.71 (2.40- 3.02) | 2.26 (2.19- 2.33) | 2.61 (2.35- 2.87) | 2.44 (2.18- 2.69) | 2.46 (2.11- 2.80) | 2.42 (2.04- 2.80) | 2.30 (2.19- 2.42) | 2.67 (2.39- 2.94) | 2.32 (1.99- 2.67) | 1.89 (1.47- 2.31) | 2.83 (2.28- 3.38) | 2.39 (2.21- 2.56) |
| HR_Cr_ (95% CI) | Ref  1.0 | 0.76 (0.62- 0.94) | 0.77 (0.61- 0.98) | 0.75 (0.56- 1.00) | 0.73 (0.63- 0.85) | Ref  1.0 | 0.89 (0.80- 0.99) | 0.84 (0.74- 0.96) | 0.95 (0.83- 1.09) | 0.81 (0.74- 0.88) | Ref  1.0 | 0.93 (0.80- 1.07) | 0.93 (0.79- 1.11) | 0.92 (0.76- 1.11) | 0.89 (0.80- 0.99) | Ref  1.0 | 0.87 (0.73- 1.04) | 0.71 (0.55- 0.90) | 1.05 (0.85- 1.31) | 0.90 (0.80- 1.03) |
| HR_Adj1_ (95% CI) | Ref  1.0 | 0.83 (0.67- 1.04) | 0.85 (0.66- 1.10) | 0.80 (0.59- 1.09) | 0.81 (0.69- 0.95) | Ref  1.0 | 0.93 (0.83- 1.05) | 0.92 (0.80- 1.05) | 0.95 (0.82- 1.10) | 0.87 (0.79- 0.95) | Ref  1.0 | 0.95 (0.81- 1.11) | 0.97 (0.81- 1.17) | 0.92 (0.75- 1.12) | 0.90 (0.79- 1.01) | Ref  1.0 | 0.95 (0.79- 1.16) | 0.81 (0.63- 1.04) | 1.13 (0.89- 1.43) | 0.99 (0.86- 1.13) |
| HR_Adj2_ (95% CI) | Ref  1.0 | 0.85 (0.68- 1.06) | 0.87 (0.68- 1.13) | 0.82 (0.60- 1.11) | 0.83 (0.70- 0.98) | Ref  1.0 | 0.94 (0.83- 1.06) | 0.92 (0.80- 1.06) | 0.96 (0.82- 1.11) | 0.87 (0.80- 0.96) | Ref  1.0 | 0.95 (0.81- 1.11) | 0.97 (0.81- 1.17) | 0.92 (0.75- 1.12) | 0.90 (0.79- 1.01) | Ref  1.0 | 0.95 (0.78- 1.15) | 0.80 (0.62- 1.03) | 1.12 (0.88- 1.42) | 0.98 (0.85- 1.12) |
| HR_Adj3_ (95% CI) | Ref  1.0 | 0.86 (0.69- 1.08) | 0.89 (0.69- 1.15) | 0.82 (0.60- 1.12) | 0.85 (0.72-1.00) | Ref  1.0 | 0.95 (0.84- 1.07) | 0.93 (0.81- 1.07) | 0.97 (0.83- 1.13) | 0.89 (0.81- 0.98) | Ref  1.0 | 0.95 (0.82- 1.11) | 0.98 (0.82- 1.17) | 0.92 (0.75- 1.13) | 0.90 (0.80- 1.02) | Ref  1.0 | 0.96 (0.79- 1.17) | 0.82 (0.63- 1.06) | 1.13 (0.89- 1.43) | 0.99 (0.86- 1.14) |
| **Girls** | | | | | | | | | | | | | | | | | | | | |
| Event,  n | 79 | 59 | 33 | 26 | 661 | 227 | 229 | 149 | 80 | 2004 | 147 | 137 | 73 | 64 | 655 | 119 | 77 | 42 | 35 | 316 |
| IR (95% CI) | 1.41 (1.10- 1.72) | 1.14 (0.85- 1.44) | 1.04 (0.69- 1.40) | 1.30 (0.80- 1.80) | 1.16 (1.07- 1.24) | 1.27 (1.10- 1.43) | 1.02 (0.88- 1.15) | 1.11 (0.93- 1.29) | 0.88 (0.69- 1.07) | 1.04 (0.99- 1.08) | 1.26 (1.06- 1.47) | 1.25 (1.04- 1.45) | 1.16 (0.89- 1.43) | 1.36 (1.03- 1.69) | 0.98 (0.91- 1.06) | 1.35 (1.11- 1.60) | 1.48 (1.15- 1.82) | 1.47 (1.03- 1.92) | 1.50 (1.00- 2.00) | 1.28 (1.14- 1.42) |
| HR_Cr_ (95% CI) | Ref  1.0 | 0.81 (0.58- 1.13) | 0.74 (0.49- 1.11) | 0.92 (0.59- 1.43) | 0.82 (0.65- 1.03) | Ref  1.0 | 0.80 (0.67- 0.96) | 0.87 (0.71- 1.07) | 0.69 (0.54- 0.89) | 0.81 (0.71- 0.93) | Ref  1.0 | 0.98 (0.77- 1.23) | 0.91 (0.69- 1.20) | 1.07 (0.79- 1.43) | 0.77 (0.64- 0.92) | Ref  1.0 | 1.09 (0.82- 1.45) | 1.07 (0.75- 1.52) | 1.11 (0.76- 1.62) | 0.93 (0.76- 1.15) |
| HR_Adj1_ (95% CI) | Ref  1.0 | 0.93 (0.64- 1.35) | 0.80 (0.51- 1.26) | 1.13 (0.71- 1.82) | 0.96 (0.74- 1.26) | Ref  1.0 | 0.83 (0.68- 1.01) | 0.92 (0.74- 1.15) | 0.69 (0.52- 0.92) | 0.86 (0.74- 1.00) | Ref  1.0 | 1.00 (0.78- 1.30) | 0.93 (0.68- 1.27) | 1.10 (0.80- 1.53) | 0.87 (0.71- 1.06) | Ref  1.0 | 1.09 (0.79- 1.49) | 1.04 (0.70- 1.55) | 1.14 (0.76- 1.73) | 1.03 (0.82- 1.30) |
| HR_Adj2_ (95% CI) | Ref  1.0 | 0.93 (0.64- 1.35) | 0.80 (0.51- 1.27) | 1.13 (0.71- 1.82) | 0.97 (0.74- 1.27) | Ref  1.0 | 0.85 (0.70- 1.04) | 0.94 (0.76- 1.18) | 0.71 (0.54- 0.94) | 0.89 (0.76- 1.03) | Ref  1.0 | 1.02 (0.78- 1.32) | 0.94 (0.69- 1.29) | 1.12 (0.81- 1.54) | 0.88 (0.72- 1.07) | Ref  1.0 | 1.10 (0.80- 1.51) | 1.05 (0.70- 1.57) | 1.15 (0.76- 1.74) | 1.04 (0.83- 1.32) |
| HR_Adj3_ (95% CI) | Ref  1.0 | 0.94 (0.65- 1.36) | 0.81 (0.52- 1.28) | 1.13 (0.71- 1.82) | 0.97 (0.74- 1.28) | Ref  1.0 | 0.86 (0.70- 1.05) | 0.95 (0.76- 1.19) | 0.71 (0.54- 0.95) | 0.89 (0.77- 1.04) | Ref  1.0 | 1.03 (0.79- 1.33) | 0.95 (0.70- 1.30) | 1.13 (0.81- 1.56) | 0.89 (0.73- 1.08) | Ref  1.0 | 1.09 (0.79- 1.51) | 1.05 (0.70- 1.56) | 1.15 (0.76- 1.74) | 1.04 (0.82- 1.32) |
| HRs were estimated with Cox proportional hazards models. Crude (HR_cr_) and adjusted (HR_Adj_) models compare vacuum-assisted delivery (stratified by fetal head station: outlet, mid/low) and spontaneous vaginal delivery with emergency caesarean delivery as the reference category. HR_Adj1_: Adjusted for maternal age, smoking, maternal BMI, diabetes mellitus type 1 or 2, birthyear. HR_Adj2_: Adjusted for variables in model one and gestational diabetes, preeclampsia and chorioamnionitis. HR_Adj3_: Adjusted for variables of model 1 and 2 and additionally for maternal educational level and maternal pre-pregnancy comorbidities (ADHD, ASD, depression, anxiety disorder).  CI = confidence interval; ECD, Emergency cesarean delivery; IR = incidence rate (per 1 000 person-years); SVD, Spontaneous vaginal delivery | | | | | | | | | | | | | | | | | | | | |

## **Table H. Incidence and hazard ratios of cerebral palsy** in children by mode of delivery, comparing vacuum-assisted delivery and spontaneous vaginal delivery with emergency caesarean delivery as reference, **stratified by gestational age at delivery categories.**

|  | **Early term (37+0-38+6)** | | | | | **Full term (39+0-40+6)** | | | | | **Late term (41+0-41+6)** | | | | | **Post term (≥42+0)** | | | | |
| --- | --- | --- | --- | --- | --- | --- | --- | --- | --- | --- | --- | --- | --- | --- | --- | --- | --- | --- | --- | --- |
|  |  | **Vacuum-assisted delivery** | | |  |  | **Vacuum-assisted delivery** | | |  |  | **Vacuum-assisted delivery** | | |  |  | **Vacuum-assisted delivery** | | |  |
|  | **ECD** | **Total** | **Outlet** | **Mid/Low** | **SVD** | **ECD** | **Total** | **Outlet** | **Mid/Low** | **SVD** | **ECD** | **Total** | **Outlet** | **Mid/Low** | **SVD** | **ECD** | **Total** | **Outlet** | **Mid/low** | **SVD** |
| **All** | | | | | | | | | | | | | | | | | | | | |
| Event, n | 25 | 13 | 7 | 6 | 97 | 89 | 83 | 47 | 36 | 307 | 61 | 38 | 14 | 24 | 120 | 32 | 25 | 11 | 14 | 52 |
| IR (95% CI) | 0.21 (0.13- 0.29) | 0.11 (0.05- 0.17) | 0.10 (0.03- 0.17) | 0.13 (0.03- 0.24) | 0.08 (0.07- 0.10) | 0.22 (0.18- 0.27) | 0.17 (0.13- 0.20) | 0.16 (0.11- 0.21) | 0.18 (0.12- 0.24) | 0.08 (0.07- 0.09) | 0.23 (0.20-0.28) | 0.15 (0.10-0.19) | 0.10 (0.05-0.15) | 0.22 (0.13-0.30) | 0.09 (0.07-0.10) | 0.14 (0.09- 0.19) | 0.19 (0.12- 0.27) | 0.16 (0.06- 0.25) | 0.23 (0.11- 0.36) | 0.10 (0.07- 0.12) |
| HR_Cr_ (95% CI) | Ref  1.0 | 0.49 (0.24-1.00) | 0.43 (0.18- 1.06) | 0.57 (0.22- 1.50) | 0.39 (0.25- 0.62) | Ref  1.0 | 0.73 (0.51- 1.05) | 0.72 (0.47- 1.10) | 0.75 (0.47- 1.19) | 0.43 (0.33- 0.57) | Ref  1.0 | 0.71 (0.43- 1.18) | 0.55 (0.28- 1.09) | 0.91 (0.49- 1.68) | 0.55 (0.38- 0.81) | Ref  1.0 | 1.34 (0.71- 2.52) | 1.17 (0.52- 2.63) | 1.53 (0.70- 3.31) | 0.83 (0.49- 1.39) |
| HR_Adj1_ (95% CI) | Ref  1.0 | 0.49 (0.23- 1.03) | 0.47 (0.19- 1.16) | 0.52 (0.18- 1.50) | 0.38 (0.24- 0.62) | Ref  1.0 | 0.76 (0.53- 1.11) | 0.76 (0.49- 1.18) | 0.77 (0.47- 1.26) | 0.44 (0.33- 0.59) | Ref  1.0 | 0.76 (0.45- 1.30) | 0.65 (0.33- 1.29) | 0.90 (0.47- 1.74) | 0.58 (0.39- 0.87) | Ref  1.0 | 1.37 (0.71- 2.64) | 1.10 (0.47- 2.60) | 1.69 (0.77- 3.70) | 0.70 (0.40- 1.22) |
| HR_Adj2_ (95% CI) | Ref  1.0 | 0.53 (0.25- 1.13) | 0.51 (0.21- 1.27) | 0.56 (0.19- 1.65) | 0.42 (0.26- 0.70) | Ref  1.0 | 0.78 (0.53- 1.14) | 0.78 (0.50- 1.21) | 0.78 (0.48- 1.28) | 0.45 (0.33- 0.61) | Ref  1.0 | 0.76 (0.45- 1.30) | 0.65 (0.33- 1.29) | 0.90 (0.46- 1.74) | 0.58 (0.39- 0.87) | Ref  1.0 | 1.39 (0.72- 2.68) | 1.12 (0.47- 2.64) | 1.71 (0.78- 3.74) | 0.71 (0.41- 1.25) |
| HR_Adj3_ (95% CI) | Ref  1.0 | 0.54 (0.25- 1.15) | 0.52 (0.21- 1.29) | 0.57 (0.20- 1.66) | 0.43 (0.26- 0.71) | Ref  1.0 | 0.79 (0.54- 1.15) | 0.79 (0.51- 1.22) | 0.79 (0.48- 1.30) | 0.45 (0.34- 0.61) | Ref  1.0 | 0.77 (0.45- 1.32) | 0.66 (0.33- 1.31) | 0.91 (0.47- 1.76) | 0.58 (0.39- 0.88) | Ref  1.0 | 1.38 (0.72- 2.67) | 1.12 (0.47- 2.64) | 1.69 (0.77- 3.71) | 0.71 (0.41- 1.25) |
| **Boys** | | | | | | | | | | | | | | | | | | | | |
| Event, n | 15 | 7 | 3 | 4 | 49 | 50 | 53 | 26 | 27 | 183 | 36 | 23 | 11 | 12 | 60 | 12 | 15 | 6 | 9 | 30 |
| IR (95% CI) | 0.24 (0.12- 0.36) | 0.11 (0.03- 0.19) | 0.08 (-0.01- 0.16) | 0.16 (0.00- 0.32) | 0.09 (0.06- 0.11) | 0.23 (0.17- 0.29) | 0.20 (0.14- 0.25) | 0.16 (0.10-0.23) | 0.24 (0.15-0.33) | 0.10 (0.09-0.12) | 0.24 (0.16-0.31) | 0.16 (0.09-0.22) | 0.13 (0.05-0.21) | 0.19 (0.08-0.29) | 0.09 (0.07-0.11) | 0.09 (0.04-0.14) | 0.19 (0.09-0.29) | 0.14 (0.03-0.26) | 0.25 (0.09-0.41) | 0.10 (0.07-0.14) |
| HR_Cr_ (95% CI) | Ref  1.0 | 0.42 (0.16- 1.09) | 0.34 (0.10- 1.19) | 0.54 (0.16- 1.88) | 0.33 (0.18- 0.61) | Ref  1.0 | 0.89 (0.56- 1.41) | 0.78 (0.45- 1.36) | 1.04 (0.59- 1.82) | 0.49 (0.34- 0.71) | Ref  1.0 | 0.74 (0.40-1.38) | 0.62 (0.28-1.39) | 0.89 (0.41-1.91) | 0.46 (0.28-0.74) | Ref  1.0 | 2.21 (0.87- 5.59) | 1.66 (0.50- 5.50) | 2.84 (0.98- 8.17) | 1.41 (0.63- 3.15) |
| HR_Adj1_ (95% CI) | Ref  1.0 | 0.36 (0.13- 0.99) | 0.34 (0.10- 1.18) | 0.38 (0.09- 1.69) | 0.30 (0.16- 0.57) | Ref  1.0 | 0.96 (0.59- 1.55) | 0.86 (0.49- 1.53) | 1.09 (0.61- 1.97) | 0.52 (0.35- 0.78) | Ref  1.0 | 0.89 (0.47-1.70) | 0.76 (0.34-1.73) | 1.05 (0.48-2.29) | 0.50 (0.29-0.86) | Ref  1.0 | 2.22 (0.87- 5.67) | 1.63 (0.49-5.45) | 2.93 (1.01-8.47) | 1.08 (0.47- 2.51) |
| HR_Adj2_ (95% CI) | Ref  1.0 | 0.36 (0.13- 1.02) | 0.35 (0.10- 1.22) | 0.39 (0.09- 1.75) | 0.31 (0.16- 0.59) | Ref  1.0 | 0.96 (0.59- 1.55) | 0.86 (0.49- 1.53) | 1.09 (0.60- 1.97) | 0.52 (0.35 0.78) | Ref  1.0 | 0.88 (0.46-1.69) | 0.76 (0.33-1.72) | 1.04 (0.47-2.28) | 0.50 (0.29-0.86) | Ref  1.0 | 2.18 (0.85- 5.55) | 1.60 (0.48-5.33) | 2.87 (0.99-8.31) | 1.06 (0.46- 2.46) |
| HR_Adj3_ (95% CI) | Ref  1.0 | 0.37 (0.13- 1.04) | 0.35 (0.10- 1.23) | 0.41 (0.09- 1.80) | 0.32 (0.17- 0.61) | Ref  1.0 | 0.97 (0.60- 1.57) | 0.88 (0.49- 1.56) | 1.10 (0.61- 1.99) | 0.53 (0.35- 0.79) | Ref  1.0 | 0.89 (0.47-1.70) | 0.76 (0.34-1.73) | 1.05 (0.48-2.29) | 0.51 (0.29-0.87) | Ref  1.0 | 2.18 (0.85- 5.55) | 1.61 (0.48-5.38) | 2.84 (0.98-8.22) | 1.06 (0.46- 2.47) |
| **Girls** | | | | | | | | | | | | | | | | | | | | |
| Event, n | 10 | 6 | 4 | 2 | 48 | 39 | 30 | 21 | 9 | 124 | 25 | 15 | 3 | 12 | 60 | 20 | 10 | 5 | 5 | 22 |
| IR (95% CI) | 0.18 (0.07- 0.29) | 0.12 (0.02- 0.21) | 0.13 (0.00- 0.25) | 0.10 (-0.04- 0.24) | 0.08 (0.06- 0.11) | 0.22 (0.15-0.28) | 0.13 (0.09-0.18) | 0.16 (0.09-0.22) | 0.10 (0.03-0.16) | 0.06 (0.05-0.07) | 0.21 (0.13-0.30) | 0.14 (0.07-0.20) | 0.05 (-0.01-0.10) | 0.25 (0.11-0.40) | 0.09 (0.07-0.11) | 0.23 (0.13-0.33) | 0.19 (0.07-0.31) | 0.17 (0.02-0.33) | 0.21 (0.03-0.40) | 0.09 (0.05-0.13) |
| HR_Cr_ (95% CI) | Ref  1.0 | 0.59 (0.20- 1.76) | 0.58 (0.16- 2.15) | 0.61 (0.13- 2.81) | 0.48 (0.23- 0.98) | Ref  1.0 | 0.53 (0.29-0.96) | 0.64 (0.33-1.24) | 0.36 (0.14-0.93) | 0.37 (0.24-0.56) | Ref  1.0 | 0.65 (0.27-1.56) | 0.43 (0.12-1.50) | 0.94 (0.33-2.63) | 0.73 (0.40-1.33) | Ref  1.0 | 0.84 (0.34-2.07) | 0.88 (0.29-2.67) | 0.79 (0.23-2.73) | 0.50 (0.25-0.99) |
| HR_Adj1_ (95% CI) | Ref  1.0 | 0.72 (0.24- 2.16) | 0.70 (0.19- 2.61) | 0.74 (0.16- 3.47) | 0.51 (0.24- 1.10) | Ref  1.0 | 0.53 (0.29-0.99) | 0.64 (0.32-1.27) | 0.38 (0.15-1.00) | 0.36 (0.23-0.57) | Ref  1.0 | 0.54 (0.20-1.42) | 0.47 (0.13-1.67) | 0.62 (0.18-2.19) | 0.70 (0.37-1.32) | Ref  1.0 | 0.83 (0.31-2.19) | 0.76 (0.22-2.68) | 0.91 (0.26-3.21) | 0.46 (0.22-0.98) |
| HR_Adj2_ (95% CI) | Ref  1.0 | 0.83 (0.27- 2.55) | 0.80 (0.21- 3.04) | 0.87 (0.19- 4.12) | 0.62 (0.28- 1.37) | Ref  1.0 | 0.56 (0.30-1.05) | 0.67 (0.34-1.35) | 0.40 (0.15-1.05) | 0.39 (0.24-0.61) | Ref  1.0 | 0.54 (0.20-1.43) | 0.47 (0.13-1.67) | 0.63 (0.18-2.21) | 0.70 (0.37-1.32) | Ref  1.0 | 0.86 (0.32-2.29) | 0.80 (0.22-2.83) | 0.94 (0.27 (3.31) | 0.49 (0.23-1.04) |
| HR_Adj3_ (95% CI) | Ref  1.0 | 0.85 (0.28- 2.60) | 0.83 (0.22- 3.15) | 0.87 (0.18- 4.12) | 0.63 (0.28- 1.39) | Ref  1.0 | 0.57 (0.30-1.05) | 0.68 (0.34-1.36) | 0.40 (0.15-1.06) | 0.39 (0.24-0.61) | Ref  1.0 | 0.54 (0.21-1.44) | 0.48 (0.14-1.69) | 0.63 (0.18-2.23) | 0.71 (0.37-1.33) | Ref  1.0 | 0.86 (0.32-2.28) | 0.79 (0.22-2.81) | 0.93 (0.26-3.30) | 0.48 (0.23-1.04) |
| HRs were estimated with Cox proportional hazards models. Crude (HR_cr_) and adjusted (HR_Adj_) models compare vacuum-assisted delivery (stratified by fetal head station: outlet, mid/low) and spontaneous vaginal delivery with emergency caesarean delivery as the reference category. HR_Adj1_: Adjusted for maternal age, smoking, maternal BMI, diabetes mellitus type 1 or 2, birthyear. HR_Adj2_: Adjusted for variables in model one and gestational diabetes, preeclampsia and chorioamnionitis. HR_Adj3_: Adjusted for variables of model 1 and 2 and additionally for maternal educational level and maternal pre-pregnancy comorbidities (ADHD, ASD, depression, anxiety disorder).  CI = confidence interval; ECD, Emergency cesarean delivery; IR = incidence rate (per 1 000 person-years); SVD, Spontaneous vaginal delivery | | | | | | | | | | | | | | | | | | | | |

## **Table I. Incidence and hazard ratios of epilepsy** in children by mode of delivery, comparing vacuum-assisted delivery and spontaneous vaginal delivery with emergency caesarean delivery as reference, **stratified by gestational age at delivery categories.**

|  | **Early term (37+0-38+6)** | | | | | **Full term (39+0-40+6)** | | | | | **Late term (41+0-41+6)** | | | | | **Post term (≥42+0)** | | | | |
| --- | --- | --- | --- | --- | --- | --- | --- | --- | --- | --- | --- | --- | --- | --- | --- | --- | --- | --- | --- | --- |
|  |  | **Vacuum-assisted delivery** | | |  |  | **Vacuum-assisted delivery** | | |  |  | **Vacuum-assisted delivery** | | |  |  | **Vacuum-assisted delivery** | | |  |
|  | **ECD** | **Total** | **Outlet** | **Mid/Low** | **SVD** | **ECD** | **Total** | **Outlet** | **Mid/Low** | **SVD** | **ECD** | **Total** | **Outlet** | **Mid/Low** | **SVD** | **ECD** | **Total** | **Outlet** | **Mid/low** | **SVD** |
| **All** | | | | | | | | | | | | | | | | | | | | |
| Event, n | 104 | 81 | 45 | 36 | 660 | 307 | 286 | 162 | 124 | 1947 | 177 | 159 | 82 | 77 | 728 | 141 | 93 | 46 | 47 | 304 |
| IR (95% CI) | 0.88 (0.71-1.04) | 0.70 (0.55-0.85) | 0.63 (0.45-0.82) | 0.81 (0.54-1.07) | 0.58 (0.53-0.62) | 0.78 (0.69- 0.86) | 0.58 (0.51- 0.64) | 0.55 (0.47- 0.64) | 0.61 (0.51- 0.72) | 0.52 (0.50- 0.54) | 0.66 (0.56- 0.76) | 0.62 (0.52- 0.72) | 0.56 (0.44- 0.69) | 0.69 (0.54- 0.85) | 0.54 (0.50- 0.58) | 0.62 (0.52- 0.73) | 0.72 (0.57- 0.86) | 0.66 (0.47- 0.85) | 0.79 (0.56- 1.01) | 0.56 (0.50- 0.63) |
| HR_Cr_ (95% CI) | Ref  1.0 | 0.77 (0.57- 1.05) | 0.67 (0.46- 0.98) | 0.94 (0.63- 1.40) | 0.67 (0.54- 0.84) | Ref  1.0 | 0.76 (0.64- 0.90) | 0.72 (0.59- 0.88) | 0.80 (0.64- 1.00) | 0.71 (0.63- 0.81) | Ref  1.0 | 0.95 (0.76- 1.19) | 0.88 (0.67- 1.16) | 1.04 (0.78- 1.38) | 0.83 (0.69- 0.98) | Ref  1.0 | 1.12 (0.84- 1.49) | 1.00 (0.70- 1.44) | 1.26 (0.88- 1.79) | 0.97 (0.78- 1.20) |
| HR_Adj1_ (95% CI) | Ref  1.0 | 0.75 (0.53- 1.04) | 0.65 (0.43- 0.98) | 0.90 (0.58- 1.39) | 0.68 (0.53- 0.86) | Ref  1.0 | 0.79 (0.66- 0.95) | 0.78 (0.63- 0.97) | 0.81 (0.64- 1.03) | 0.76 (0.66- 0.88) | Ref  1.0 | 0.96 (0.75- 1.22) | 0.85 (0.63- 1.14) | 1.11 (0.82- 1.49) | 0.84 (0.69- 1.01) | Ref  1.0 | 1.13 (0.84- 1.53) | 0.98 (0.66- 1.45) | 1.31 (0.90- 1.90) | 0.91 (0.73- 1.15) |
| HR_Adj2_ (95% CI) | Ref  1.0 | 0.74 (0.53- 1.03) | 0.64 (0.43- 0.97) | 0.89 (0.58- 1.38) | 0.67 (0.53- 0.85) | Ref  1.0 | 0.80 (0.67- 0.97) | 0.79 (0.64- 0.98) | 0.82 (0.64- 1.04) | 0.77 (0.67- 0.89) | Ref  1.0 | 0.95 (0.75- 1.21) | 0.84 (0.63- 1.13) | 1.10 (0.82- 1.48) | 0.83 (0.69- 1.00) | Ref  1.0 | 1.12 (0.83- 1.52) | 0.98 (0.66- 1.44) | 1.30 (0.90- 1.89) | 0.91 (0.72- 1.15) |
| HR_Adj3_ (95% CI) | Ref  1.0 | 0.74 (0.53-1.03) | 0.64 (0.42- 0.97) | 0.89 (0.58- 1.38) | 0.67 (0.53- 0.85) | Ref  1.0 | 0.81 (0.67- 0.98) | 0.80 (0.64-1.00) | 0.83 (0.65- 1.05) | 0.78 (0.68- 0.90) | Ref  1.0 | 0.95 (0.75- 1.22) | 0.84 (0.63- 1.14) | 1.10 (0.82- 1.48) | 0.83 (0.69- 1.00) | Ref  1.0 | 1.13 (0.84- 1.53) | 0.98 (0.66- 1.45) | 1.31 (0.90- 1.90) | 0.91 (0.72- 1.15) |
| **Boys** | | | | | | | | | | | | | | | | | | | | |
| Event, n | 66 | 50 | 25 | 25 | 352 | 161 | 183 | 98 | 85 | 974 | 95 | 98 | 46 | 390 | 390 | 84 | 59 | 32 | 27 | 176 |
| IR (95% CI) | 1.05 (0.80-1.31) | 0.78 (0.57-1.00) | 0.64 (0.39-0.88) | 1.02 (0.62-1.42) | 0.62 (0.55-0.68) | 0.74 (0.63- 0.86) | 0.68 (0.58- 0.78) | 0.62 (0.49- 0.74) | 0.77 (0.60- 0.93) | 0.54 (0.50- 0.57) | 0.63 (0.50- 0.75) | 0.67 (0.54- 0.80) | 0.56 (0.40- 0.72) | 0.57 (0.51- 0.63) | 0.57 (0.51- 0.63) | 0.61 (0.48- 0.74) | 0.76 (0.56- 0.95) | 0.77 (0.51- 1.04) | 0.74 (0.46- 1.02) | 0.61 (0.52- 0.70) |
| HR_Cr_ (95% CI) | Ref  1.0 | 0.71 (0.48-1.05) | 0.56 (0.34-0.93) | 0.94 (0.58-1.53) | 0.58 (0.44-0.77) | Ref  1.0 | 0.91 (0.73- 1.14) | 0.82 (0.63- 1.07) | 1.04 (0.79- 1.37) | 0.76 (0.64- 0.91) | Ref  1.0 | 1.13 (0.84- 1.52) | 0.95 (0.65- 1.38) | 1.35 (0.95- 1.94) | 0.95 (0.75- 1.21) | Ref  1.0 | 1.23 (0.86- 1.76) | 1.25 (0.80- 1.94) | 1.20 (0.75- 1.93) | 1.06 (0.80- 1.40) |
| HR_Adj1_ (95% CI) | Ref  1.0 | 0.74 (0.49-1.13) | 0.59 (0.35-1.00) | 0.99 (0.58-1.67) | 0.61 (0.45-0.83) | Ref  1.0 | 0.94 (0.74- 1.20) | 0.89 (0.67- 1.18) | 1.01 (0.75- 1.37) | 0.81 (0.67- 0.99) | Ref  1.0 | 1.22 (0.89- 1.67) | 1.02 (0.69- 1.50) | 1.48 (1.02- 2.15) | 0.99 (0.76- 1.28) | Ref  1.0 | 1.22 (0.83- 1.79) | 1.23 (0.77- 1.97) | 1.20 (0.73- 1.98) | 1.03 (0.76- 1.39) |
| HR_Adj2_ (95% CI) | Ref  1.0 | 0.74 (0.49-1.13) | 0.59 (0.35-1.01) | 0.99 (0.58-1.68) | 0.61 (0.45-0.84) | Ref  1.0 | 0.94 (0.74- 1.20) | 0.89 (0.67- 1.18) | 1.01 (0.75- 1.37) | 0.82 (0.67- 0.99) | Ref  1.0 | 1.22 (0.89- 1.67) | 1.01 (0.69- 1.49) | 1.47 (1.02- 2.14) | 0.99 (0.76- 1.28) | Ref  1.0 | 1.21 (0.82- 1.77) | 1.22 (0.76- 1.95) | 1.19 (0.72- 1.97) | 1.02 (0.75- 1.37) |
| HR_Adj3_ (95% CI) | Ref  1.0 | 0.74 (0.48-1.13) | 0.58 (0.34-0.99) | 1.00 (0.59-1.69) | 0.61 (0.45-0.83) | Ref  1.0 | 0.96 (0.75- 1.22) | 0.90 (0.68- 1.20) | 1.03 (0.76- 1.40) | 0.83 (0.68- 1.01) | Ref  1.0 | 1.22 (0.89- 1.68) | 1.02 (0.69- 1.50) | 1.48 (1.02- 2.15) | 0.99 (0.77- 1.29) | Ref  1.0 | 1.20 (0.82- 1.77) | 1.21 (0.75- 1.94) | 1.20 (0.72- 1.98) | 1.02 (0.75- 1.38) |
| **Girls** | | | | | | | | | | | | | | | | | | | | |
| Event, n | 38 | 31 | 20 | 11 | 308 | 146 | 103 | 64 | 39 | 973 | 82 | 61 | 36 | 25 | 338 | 57 | 34 | 14 | 20 | 128 |
| IR (95% CI) | 0.68 (0.46-0.89) | 0.60 (0.39-0.81) | 0.63 (0.36-0.91) | 0.55 (0.22-0.87) | 0.54 (0.48-0.60) | 0.82 (0.68- 0.95) | 0.46 (0.37- 0.55) | 0.48 (0.36- 0.59) | 0.43 (0.29- 0.56) | 0.50 (0.47- 0.53) | 0.71 (0.55-0.86) | 0.55 (0.42-0.69) | 0.57 (0.38-0.76) | 0.53 (0.32-0.74) | 0.51 (0.45-0.56) | 0.65 (0.48- 0.82) | 0.65 (0.43- 0.87) | 0.49 (0.23- 0.75) | 0.86 (0.48- 1.23) | 0.52 (0.43- 0.61) |
| HR_Cr_ (95% CI) | Ref  1.0 | 0.87 (0.53-1.45) | 0.85 (0.47-1.54) | 0.91 (0.46-1.81) | 0.83 (0.58-1.19) | Ref  1.0 | 0.58 (0.45- 0.76) | 0.61 (0.45- 0.83) | 0.54 (0.37- 0.78) | 0.66 (0.55- 0.79) | Ref  1.0 | 0.75 (0.53- 1.07) | 0.81 (0.54- 1.22) | 0.68 (0.42- 1.11) | 0.70 (0.54- 0.90) | Ref  1.0 | 0.97 (0.61- 1.53) | 0.67 (0.35- 1.29) | 1.33 (0.77- 2.28) | 0.86 (0.61- 1.19) |
| HR_Adj1_ (95% CI) | Ref  1.0 | 0.74 (0.42-1.29) | 0.74 (0.39-1.43) | 0.74 (0.34-1.60) | 0.80 (0.55-1.17) | Ref  1.0 | 0.62 (0.47- 0.83) | 0.66 (0.47- 0.92) | 0.57 (0.38- 0.85) | 0.71 (0.58- 0.88) | Ref  1.0 | 0.67 (0.46-0.99) | 0.67 (0.42-1.06) | 0.68 (0.40-1.14) | 0.69 (0.53-0.91) | Ref  1.0 | 1.01 (0.63- 1.64) | 0.64 (0.31- 1.31) | 1.47 (0.84- 2.57) | 0.78 (0.55- 1.12) |
| HR_Adj2_ (95% CI) | Ref  1.0 | 0.72 (0.41-1.25) | 0.72 (0.38-1.38) | 0.71 (0.33-1.55) | 0.77 (0.52-1.13) | Ref  1.0 | 0.64 (0.48- 0.86) | 0.68 (0.48- 0.95) | 0.58 (0.39- 0.87) | 0.74 (0.60- 0.91) | Ref  1.0 | 0.66 (0.45-0.98) | 0.66 (0.41-1.05) | 0.67 (0.40-1.13) | 0.68 (0.52-0.90) | Ref  1.0 | 1.01 (0.62- 1.65) | 0.64 (0.31- 1.31) | 1.46 (0.84- 2.56) | 0.79 (0.55- 1.12) |
| HR_Adj3_ (95% CI) | Ref  1.0 | 0.72 (0.41-1.26) | 0.72 (0.38-1.39) | 0.72 (0.33-1.57) | 0.78 (0.53-1.14) | Ref  1.0 | 0.64 (0.48- 0.86) | 0.68 (0.49- 0.96) | 0.59 (0.39- 0.88) | 0.74 (0.60- 0.92) | Ref  1.0 | 0.66 (0.45-0.97) | 0.66 (0.41-1.05) | 0.67 (0.40-1.12) | 0.68 (0.51-0.89) | Ref  1.0 | 1.02 (0.63- 1.66) | 0.64 (0.31- 1.32) | 1.48 (0.85- 2.59) | 0.79 (0.55- 1.13) |
| HRs were estimated with Cox proportional hazards models. Crude (HR_cr_) and adjusted (HR_Adj_) models compare vacuum-assisted delivery (stratified by fetal head station: outlet, mid/low) and spontaneous vaginal delivery with emergency caesarean delivery as the reference category. HR_Adj1_: Adjusted for maternal age, smoking, maternal BMI, diabetes mellitus type 1 or 2, birthyear. HR_Adj2_: Adjusted for variables in model one and gestational diabetes, preeclampsia and chorioamnionitis. HR_Adj3_: Adjusted for variables of model 1 and 2 and additionally for maternal educational level and maternal pre-pregnancy comorbidities (ADHD, ASD, depression, anxiety disorder).  CI = confidence interval; ECD, Emergency cesarean delivery; IR = incidence rate (per 1 000 person-years); SVD, Spontaneous vaginal delivery | | | | | | | | | | | | | | | | | | | | |

## **Table J. Incidence and hazard ratios of intellectual disability** in children by mode of delivery, comparing vacuum-assisted delivery and spontaneous vaginal delivery with emergency caesarean delivery as reference, **stratified by gestational age at delivery categories.**

|  | **Early term (37+0-38+6)** | | | | | **Full term (39+0-40+6)** | | | | | **Late term (41+0-41+6)** | | | | | **Post term (≥42+0)** | | | | |
| --- | --- | --- | --- | --- | --- | --- | --- | --- | --- | --- | --- | --- | --- | --- | --- | --- | --- | --- | --- | --- |
|  |  | **Vacuum-assisted delivery** | | |  |  | **Vacuum-assisted delivery** | | |  |  | **Vacuum-assisted delivery** | | |  |  | **Vacuum-assisted delivery** | | |  |
|  | **ECD** | **Total** | **Outlet** | **Mid/Low** | **SVD** | **ECD** | **Total** | **Outlet** | **Mid/Low** | **SVD** | **ECD** | **Total** | **Outlet** | **Mid/Low** | **SVD** | **ECD** | **Total** | **Outlet** | **Mid/low** | **SVD** |
| **All** | | | | | | | | | | | | | | | | | | | | |
| Event (n) | 94 | 66 | 34 | 32 | 542 | 231 | 202 | 131 | 71 | 1400 | 141 | 112 | 56 | 56 | 526 | 133 | 66 | 45 | 21 | 231 |
| IR (95% CI) | 0.74 (0.59- 0.89) | 0.53 (0.40- 0.66) | 0.45 (0.30- 0.60) | 0.69 (0.44- 0.90) | 0.44 (0.41- 0.48) | 0.54 (0.47- 0.61) | 0.38 (0.33- 0.43) | 0.42 (0.34- 0.49) | 0.33 (0.25- 0.40) | 0.35 (0.33- 0.37) | 0.49 (0.41- 0.57) | 0.41 (0.33- 0.48) | 0.36 (0.26- 0.45) | 0.47 (0.35- 0.59) | 0.36 (0.33- 0.39) | 0.55 (0.45-0.64) | 0.47 (0.36-0.59) | 0.60 (0.42-0.77) | 0.33 (0.19-0.47) | 0.40 (0.35-0.45) |
| HR_Cr_ (95% CI) | Ref  1.0 | 0.71 (0.52- 0.98) | 0.60 (0.40- 0.89) | 0.89 (0.60- 1.33) | 0.61 (0.49- 0.76) | Ref  1.0 | 0.69 (0.57- 0.84) | 0.76 (0.62- 0.95) | 0.59 (0.45- 0.77) | 0.65 (0.56- 0.74) | Ref  1.0 | 0.83 (0.65- 1.06) | 0.73 (0.54-1.00) | 0.95 (0.70- 1.29) | 0.75 (0.62- 0.91) | Ref  1.0 | 0.86 (0.64- 1.15) | 1.09 (0.76- 1.52) | 0.59 (0.37- 0.93) | 0.74 (0.60- 0.92) |
| HR_Adj1_ (95% CI) | Ref  1.0 | 0.82 (0.58- 1.14) | 0.68 (0.45- 1.03) | 1.04 (0.68- 1.59) | 0.64 (0.50- 0.81) | Ref  1.0 | 0.72 (0.58- 0.88) | 0.80 (0.64- 1.01) | 0.58 (0.44- 0.78) | 0.65 (0.56- 0.75) | Ref  1.0 | 0.90 (0.69- 1.18) | 0.77 (0.55- 1.08) | 1.08 (0.77- 1.50) | 0.77 (0.63- 0.95) | Ref  1.0 | 0.90 (0.66-1.23) | 1.13 (0.79- 1.62) | 0.63 (0.39- 1.02) | 0.73 (0.58- 0.92) |
| HR_Adj2_ (95% CI) | Ref  1.0 | 0.82 (0.59- 1.15) | 0.69 (0.46- 1.04) | 1.04 (0.68- 1.59) | 0.64 (0.50- 0.82) | Ref  1.0 | 0.73 (0.60- 0.90) | 0.82 (0.65- 1.03) | 0.60 (0.45 - 0.80) | 0.66 (0.57- 0.77) | Ref  1.0 | 0.92 (0.71- 1.21) | 0.79 (0.56- 1.11) | 1.10 (0.79- 1.54) | 0.80 (0.65- 0.98) | Ref  1.0 | 0.90 (0.66-1.23) | 1.13 (0.79- 1.61) | 0.63 (0.39- 1.02) | 0.73 (0.58- 0.92) |
| HR_Adj3_ (95% CI) | Ref  1.0 | 0.84 (0.60- 1.18) | 0.70 (0.46- 1.06) | 1.07 (0.70- 1.64) | 0.67 (0.52- 0.85) | Ref  1.0 | 0.76 (0.62- 0.93) | 0.85 (0.68- 1.07) | 0.62 (0.46- 0.83) | 0.69 (0.59- 0.81) | Ref  1.0 | 0.96 (0.74- 1.26) | 0.83 (0.59- 1.16) | 1.15 (0.82- 1.60) | 0.82 (0.66- 1.01) | Ref  1.0 | 0.93 (0.68-1.27) | 1.17 (0.82-1.68) | 0.64 (0.40-1.05) | 0.75 (0.60-0.95) |
| **Boys** | | | | | | | | | | | | | | | | | | | | |
| Event (n) | 58 | 37 | 18 | 19 | 329 | 157 | 121 | 80 | 41 | 894 | 95 | 76 | 37 | 39 | 330 | 81 | 45 | 32 | 13 | 144 |
| IR (95% CI) | 0.86 (0.64- 1.09) | 0.54 (0.37- 0.71) | 0.43 (0.23- 0.62) | 0.72 (0.40- 1.05) | 0.54 (0.48- 0.60) | 0.67 (0.57-0.78) | 0.42 (0.34-0.49) | 0.47 (0.37-0.57) | 0.34 (0.24-0.45) | 0.46 (0.43-0.49) | 0.58 (0.46- 0.70) | 0.48 (0.37- 0.59) | 0.42 (0.28- 0.55) | 0.57 (0.39- 0.75) | 0.45 (0.40- 0.50) | 0.55 (0.43-0.66) | 0.54 (0.38-0.69) | 0.72 (0.47-0.97) | 0.33 (0.15-0.51) | 0.46 (0.39-0.54) |
| HR_Cr_ (95% CI) | Ref  1.0 | 0.62 (0.41- 0.94) | 0.49 (0.29- 0.83) | 0.83 (0.49- 1.39) | 0.64 (0.48- 0.84) | Ref  1.0 | 0.61 (0.48- 0.78) | 0.69 (0.53- 0.91) | 0.50 (0.35- 0.70) | 0.69 (0.58- 0.82) | Ref  1.0 | 0.83 (0.61- 1.12) | 0.72 (0.49- 1.05) | 0.97 (0.67- 1.41) | 0.79 (0.63- 0.99) | Ref  1.0 | 0.98 (0.68-1.41) | 1.31 (0.87-1.97) | 0.60 (0.34-1.08) | 0.86 (0.66-1.13) |
| HR_Adj1_ (95% CI) | Ref  1.0 | 0.71 (0.46- 1.10) | 0.53 (0.30- 0.93) | 1.02 (0.59- 1.75) | 0.69 (0.50- 0.94) | Ref  1.0 | 0.66 (0.51- 0.85) | 0.76 (0.57- 1.02) | 0.51 (0.35- 0.74) | 0.70 (0.58- 0.85) | Ref  1.0 | 0.92 (0.67- 1.28) | 0.78 (0.52- 1.17) | 1.11 (0.74- 1.65) | 0.85 (0.66- 1.09) | Ref  1.0 | 1.03 (0.70-1.51) | 1.35 (0.88-2.07) | 0.65 (0.35-1.20) | 0.86 (0.64-1.15) |
| HR_Adj2_ (95% CI) | Ref  1.0 | 0.72 (0.46- 1.12) | 0.54 (0.30- 0.95) | 1.03 (0.60- 1.77) | 0.70 (0.51- 0.96) | Ref  1.0 | 0.67 (0.52- 0.87) | 0.78 (0.59- 1.04) | 0.52 (0.36- 0.76) | 0.72 (0.60- 0.87) | Ref  1.0 | 0.95 (0.68- 1.32) | 0.80 (0.53- 1.21) | 1.14 (0.76- 1.70) | 0.88 (0.68- 1.14) | Ref  1.0 | 1.03 (0.70-1.51) | 1.35 (0.88-2.08) | 0.65 (0.35-1.20) | 0.86 (0.64-1.15) |
| HR_Adj3_ (95% CI) | Ref  1.0 | 0.74 (0.48- 1.16) | 0.55 (0.31- 0.97) | 1.07 (0.62- 1.84) | 0.73 (0.53- 1.00) | Ref  1.0 | 0.69 (0.54- 0.90) | 0.80 (0.60- 1.07) | 0.54 (0.37- 0.79) | 0.76 (0.63- 0.91) | Ref  1.0 | 1.01 (0.72- 1.40) | 0.85 (0.56- 1.29) | 1.21 (0.81- 1.81) | 0.92 (0.71- 1.19) | Ref  1.0 | 1.08 (0.74-1.59) | 1.43 (0.93-2.20) | 0.67 (0.36-1.24) | 0.88 (0.66-1.19) |
| **Girls** | | | | | | | | | | | | | | | | | | | | |
| Event (n) | 36 | 29 | 16 | 13 | 213 | 74 | 81 | 51 | 30 | 506 | 46 | 36 | 19 | 17 | 196 | 52 | 21 | 13 | 8 | 87 |
| IR (95% CI) | 0.60 (0.40- 0.79) | 0.52 (0.33- 0.71) | 0.47 (0.24- 0.70) | 0.60 (0.28- 0.93) | 0.35 (0.30- 0.39) | 0.38 (0.30-0.47) | 0.33 (0.26-0.41) | 0.35 (0.26-0.45) | 0.31 (0.20-0.42) | 0.24 (0.22-0.26) | 0.37 (0.26- 0.47) | 0.30 (0.20- 0.40) | 0.28 (0.15- 0.41) | 0.34 (0.18- 0.49) | 0.27 (0.23- 0.31) | 0.55 (0.40-0.70) | 0.38 (0.21-0.54) | 0.42 (0.19-0.65) | 0.32 (0.10-0.54) | 0.33 (0.26-0.40) |
| HR_Cr_ (95% CI) | Ref  1.0 | 0.86 (0.53- 1.40) | 0.78 (0.43- 1.40) | 0.99 (0.53- 1.87) | 0.58 (0.41- 0.83) | Ref  1.0 | 0.87 (0.63-1.19) | 0.92 (0.64-1.31) | 0.79 (0.52-1.21) | 0.64 (0.50-0.82) | Ref  1.0 | 0.82 (0.53- 1.27) | 0.76 (0.45- 1.30) | 0.90 (0.52 1.57) | 0.75 (0.55- 1.04) | Ref  1.0 | 0.67 (0.41-1.12) | 0.76 (0.42-1.40) | 0.57 (0.27-1.19) | 0.60 (0.43-0.85) |
| HR_Adj1_ (95% CI) | Ref  1.0 | 0.99 (0.59- 1.70) | 0.95 (0.52- 1.75) | 1.06 (0.53- 2.10) | 0.59 (0.40- 0.87) | Ref  1.0 | 0.85 (0.61-1.20) | 0.92 (0.63-1.35) | 0.75 (0.47-1.20) | 0.64 (0.49-0.83) | Ref  1.0 | 0.86 (0.54- 1.38) | 0.77 (0.43- 1.37) | 1.00 (0.55- 1.82) | 0.72 (0.51- 1.02) | Ref  1.0 | 0.71 (0.41-1.22) | 0.79 (0.41-1.53) | 0.61 (0.28-1.35) | 0.58 (0.40-0.85) |
| HR_Adj2_ (95% CI) | Ref  1.0 | 0.98 (0.58- 1.65) | 0.94 (0.51- 1.73) | 1.04 (0.52- 2.08) | 0.58 (0.39- 0.86) | Ref  1.0 | 0.86 (0.61-1.22) | 0.93 (0.63-1.37) | 0.76 (0.48-1.22) | 0.64 (0.49-0.84) | Ref  1.0 | 0.88 (0.55- 1.41) | 0.78 (0.43- 1.39) | 1.02 (0.56- 1.84) | 0.73 (0.51- 1.04) | Ref  1.0 | 0.70 (0.41-1.22) | 0.79 (0.41-1.53) | 0.60 (0.27-1.34) | 0.58 (0.40-0.85) |
| HR_Adj3_ (95% CI) | Ref  1.0 | 0.99 (0.59- 1.66) | 0.94 (0.51- 1.73) | 1.06 (0.53- 2.12) | 0.59 (0.40- 0.88) | Ref  1.0 | 0.89 (0.63-1.26) | 0.97 (0.66-1.42) | 0.79 (0.49-1.26) | 0.67 (0.51-0.88) | Ref  1.0 | 0.89 (0.55- 1.42) | 0.79 (0.44- 1.41) | 1.02 (0.56- 1.85) | 0.73 (0.51- 1.04) | Ref  1.0 | 0.71 (0.41-1.23) | 0.80 (0.41-1.54) | 0.61 (0.27-1.35) | 0.60 (0.41-0.87) |
| HRs were estimated with Cox proportional hazards models. Crude (HR_cr_) and adjusted (HR_Adj_) models compare vacuum-assisted delivery (stratified by fetal head station: outlet, mid/low) and spontaneous vaginal delivery with emergency caesarean delivery as the reference category. HR_Adj1_: Adjusted for maternal age, smoking, maternal BMI, diabetes mellitus type 1 or 2, birthyear. HR_Adj2_: Adjusted for variables in model one and gestational diabetes, preeclampsia and chorioamnionitis. HR_Adj3_: Adjusted for variables of model 1 and 2 and additionally for maternal educational level and maternal pre-pregnancy comorbidities (ADHD, ASD, depression, anxiety disorder).  CI = confidence interval; ECD, Emergency cesarean delivery; IR = incidence rate (per 1 000 person-years); SVD, Spontaneous vaginal delivery | | | | | | | | | | | | | | | | | | | | |

## **Table K. Incidence and hazard ratios of attention-deficit/hyperactivity disorder** in children by mode of delivery, comparing vacuum-assisted delivery and spontaneous vaginal delivery with emergency caesarean delivery as reference, **stratified by birth cohort (children born between 1997 and 2006 and 2007 or later).**

|  | **Birth year 1997-2006** | | | | | **Birth year ≥2007** | | | | |
| --- | --- | --- | --- | --- | --- | --- | --- | --- | --- | --- |
|  |  | **Vacuum-assisted delivery** | | |  |  | **Vacuum-assisted delivery** | | |  |
|  | **ECD** | **Total** | **Outlet** | **Mid/Low** | **SVD** | **ECD** | **Total** | **Outlet** | **Mid/Low** | **SVD** |
| **All** | | | | | | | | | | |
| Event (n) | 3796 | 3424 | 1969 | 1455 | 23 167 | 1555 | 1427 | 773 | 654 | 8929 |
| IR (95% CI) | 5.56  (5.39-5.74) | 5.09  (4.92-5.26) | 4.98  (4.76-5.20) | 5.25  (4.98-5.52) | 4.91  (4.85-4.98) | 5.11  (4.86-5.37) | 4.68  (4.44-4.92) | 4.47  (4.16-4.79) | 4.95  (4.57-5.33) | 4.66  (4.56-4.75) |
| HR_Cr_ (95% CI) | Ref. 1.0 | 0.91  (0.86- 0.95) | 0.89  (0.84- 0.94) | 0.93  (0.88- 0.99) | 0.89  (0.86- 0.92) | Ref. 1.0 | 0.90  (0.84-0.97) | 0.86  (0.79-0.94) | 0.95  (0.87-1.04) | 0.91  (0.87-0.96) |
| HR_Adj1_ (95% CI) | Ref. 1.0 | 0.92  (0.87-0.97) | 0.90  (0.85- 0.95) | 0.95  (0.89- 1.02) | 0.87  (0.84- 0.91) | Ref. 1.0 | 0.94  (0.87-1.01) | 0.88  (0.80-0.96) | 1.02  (0.93-1.13) | 0.86  (0.81-0.91) |
| HR_Adj2_ (95% CI) | Ref. 1.0 | 0.92  (0.87-0.97) | 0.90  (0.85- 0.95) | 0.95  (0.89- 1.02) | 0.87  (0.84- 0.91) | Ref. 1.0 | 0.95  (0.88-1.02) | 0.88  (0.81-0.97) | 1.03  (0.94-1.13) | 0.87  (0.82-0.92) |
| HR_Adj3_ (95% CI) | Ref. 1.0 | 0.93  (0.88-0.98) | 0.91  (0.85-0.96) | 0.96  (0.90-1.02) | 0.88  (0.85-0.92) | Ref. 1.0 | 0.97  (0.90-1.04) | 0.90  (0.83-0.99) | 1.06  (0.96-1.16) | 0.89 (0.84-0.  95) |
| **Boys** | | | | | | | | | | |
| Event (n) | 2539 | 2343 | 1331 | 1012 | 14 196 | 1214 | 1108 | 598 | 510 | 6513 |
| IR (95% CI) | 6.69  (6.43-6.95) | 6.26  (6.00-6.51) | 6.07  (5.74-6.40) | 6.52  (6.12-6.92) | 6.17  (6.07-6.27) | 7.05  (6.66-7.45) | 6.60  (6.21-6.99) | 6.36  (5.85-6.87) | 6.90  (6.31-7.51) | 6.90  (6.73-7.06) |
| HR_Cr_ (95% CI) | Ref. 1.0 | 0.93  (0.88-0.98) | 0.90  (0.84-0.96) | 0.96  (0.89-1.03) | 0.93  (0.89-0.97) | Ref. 1.0 | 0.92  (0.85-1.00) | 0.89  (0.81-0.98) | 0.96  (0.87-1.07) | 0.98  (0.92-1.04) |
| HR_Adj1_ (95% CI) | Ref. 1.0 | 0.93  (0.88-0.99) | 0.92  (0.85-0.98) | 0.96  (0.89-1.04) | 0.91  (0.87-0.96) | Ref. 1.0 | 0.95  (0.87-1.03) | 0.89  (0.81-1.00) | 1.02  (0.92-1.14) | 0.92  (0.87-0.98) |
| HR_Adj2_ (95% CI) | Ref. 1.0 | 0.93  (0.88-0.99) | 0.92  (0.85-0.98) | 0.96  (0.89-1.04) | 0.91  (0.87-0.96) | Ref. 1.0 | 0.96  (0.88-1.04) | 0.90  (0.81-1.00) | 1.03  (0.93-1.15) | 0.93  (0.87-1.00) |
| HR_Adj3_ (95% CI) | Ref. 1.0 | 0.94  (0.89-1.00) | 0.93  (0.86-1.00) | 0.97  (0.89-1.05) | 0.93  (0.88-0.97) | Ref. 1.0 | 0.98  (0.90-1.07) | 0.93  (0.84-1.03) | 1.06  (0.95-1.18) | 0.96  (0.90-1.02) |
| **Girls** | | | | | | | | | | |
| Event (n) | 1257 | 1081 | 638 | 443 | 8971 | 341 | 319 | 175 | 144 | 2416 |
| IR (95% CI) | 4.15  (3.92-4.38) | 3.63  (3.41-3.84) | 3.62  (3.34-3.90) | 3.64  (3.30-3.98) | 3.72  (3.64-3.79) | 2.58  (2.31-2.86) | 2.33  (2.07-2.58) | 2.22  (1.89-2.55) | 2.47  (2.07-2.88) | 2.48  (2.38-2.58) |
| HR_Cr_ (95% CI) | Ref. 1.0 | 0.86  (0.80-0.94) | 0.86  (0.78-0.95) | 0.86  (0.78-0.96) | 0.89  (0.84-0.95) | Ref. 1.0 | 0.89  (0.76-1.03) | 0.84  (0.70-1.01) | 0.94  (0.77-1.14) | 0.97  (0.86-1.08) |
| HR_Adj1_ (95% CI) | Ref. 1.0 | 0.89  (0.82-0.97) | 0.87  (0.78-0.96) | 0.93  (0.83-1.05) | 0.89  (0.83-0.95) | Ref. 1.0 | 0.96  (0.82-1.13) | 0.90  (0.75-1.09) | 1.05  (0.86-1.29) | 0.94  (0.83-1.06) |
| HR_Adj2_ (95% CI) | Ref. 1.0 | 0.89  (0.81-0.97) | 0.86  (0.78-0.96) | 0.93  (0.83-1.05) | 0.89  (0.83-0.95) | Ref. 1.0 | 0.97  (0.82-1.13) | 0.91  (0.75-1.10) | 1.05  (0.86-1.29) | 0.94  (0.84-1.07) |
| HR_Adj3_ (95% CI) | Ref. 1.0 | 0.90  (0.82-0.98) | 0.87  (0.78-0.97) | 0.93  (0.83-1.05) | 0.90  (0.84-0.96) | Ref. 1.0 | 0.98  (0.84-1.15) | 0.92  (0.76-1.11) | 1.08  (0.88-1.32) | 0.97  (0.86-1.10) |

HRs were estimated with Cox proportional hazards models. Crude (HR_cr_) and adjusted (HR_Adj_) models compare vacuum-assisted delivery (stratified by fetal head station: outlet, mid/low) and spontaneous vaginal delivery with emergency caesarean delivery as the reference category. HR_Adj1_: Adjusted for maternal age, smoking, maternal BMI, diabetes mellitus type 1 or 2, birthyear. HR_Adj2_: Adjusted for variables in model one and gestational diabetes, preeclampsia and chorioamnionitis. HR_Adj3_: Adjusted for variables of model 1 and 2 and additionally for maternal educational level and maternal pre-pregnancy comorbidities (ADHD, ASD, depression, anxiety disorder).CI = confidence interval; ECD, Emergency cesarean delivery; IR = incidence rate (per 1 000 person-years); SVD, Spontaneous vaginal delivery

## **Table L. Incidence and hazard ratios of autism spectrum disorder** in children by mode of delivery, comparing vacuum-assisted delivery and spontaneous vaginal delivery with emergency caesarean delivery as reference, **stratified by birth cohort (children born between 1997 and 2006 and 2007 or later).**

|  | **Birth year 1997-2006** | | | | | **Birth year** ≥**2007** | | | | |
| --- | --- | --- | --- | --- | --- | --- | --- | --- | --- | --- |
|  |  | | | | |  | | | | |
|  |  | **Vacuum-assisted delivery** | | |  |  | **Vacuum-assisted delivery** | | |  |
|  | **ECD** | **Total** | **Outlet** | **Mid/Low** | **SVD** | **ECD** | **Total** | **Outlet** | **Mid/Low** | **SVD** |
| **All** | | | | | | | | | | |
| Event (n) | 1432 | 1246 | 713 | 533 | 7959 | 707 | 629 | 342 | 287 | 3334 |
| IR (95% CI) | 2.05  (1.94-2.16) | 1.82  (1.72-1.92) | 1.77  (1.64-1.90) | 1.89  (1.73-2.05) | 1.65  (1.62-1.69) | 2.32  (2.15-2.49) | 2.06  (1.90-2.22) | 1.97  (1.76-2.18) | 2.16  (1.91-2.42) | 1.73  (1.67-1.79) |
| HR_Cr_ (95% CI) | Ref. 1.0 | 0.87  (0.81- 0.94) | 0.85  (0.78- 0.93) | 0.90  (0.82-1.00) | 0.81  (0.77- 0.86) | Ref. 1.0 | 0.89  (0.80-0.99) | 0.85  (0.75-0.97) | 0.93  (0.81-1.07) | 0.75  (0.69-0.81) |
| HR_Adj1_ (95% CI) | Ref. 1.0 | 0.92  (0.84-1.00) | 0.91  (0.83- 1.01) | 0.92  (0.83- 1.03) | 0.89  (0.84- 0.95) | Ref. 1.0 | 0.93  (0.83-1.04) | 0.91  (0.80-1.04) | 0.96  (0.84-1.11) | 0.77  (0.71-0.84) |
| HR_Adj2_ (95% CI) | Ref. 1.0 | 0.92  (0.85-1.00) | 0.92  (0.83-1.01) | 0.93  (0.83-1.04) | 0.90  (0.84-0.95) | Ref. 1.0 | 0.94  (0.84-1.05) | 0.92  (0.80-1.05) | 0.97  (0.84-1.12) | 0.78  (0.72-0.85) |
| HR_Adj3_ (95% CI) | Ref. 1.0 | 0.93  (0.85-1.01) | 0.92  (0.84-1.02) | 0.93  (0.83-1.04) | 0.90  (0.84-0.96) | Ref. 1.0 | 0.96  (0.86-1.07) | 0.93  (0.82-1.07) | 0.99  (0.86-1.15) | 0.80  (0.73-0.87) |
| **Boys** | | | | | | | | | | |
| Event (n) | 1005 | 862 | 481 | 381 | 5132 | 562 | 511 | 277 | 234 | 2525 |
| IR (95% CI) | 2.58  (2.42-2.74) | 2.24  (2.09-2.39) | 2.14  (1.95-2.33) | 2.39  (2.15-2.63) | 2.17 (2.11-2.23) | 3.25 (2.98-3.52) | 3.03  (2.77-3.29) | 2.93  (2.59-3.28) | 3.15  (2.75-3.56) | 2.66  (2.55-2.76) |
| HR_Cr_ (95% CI) | Ref. 1.0 | 0.86  (0.79-0.94) | 0.82  (0.74-0.92) | 0.91  (0.81-1.03) | 0.85  (0.80-0.91) | Ref. 1.0 | 0.93  (0.83-1.05) | 0.90  (0.78-1.04) | 0.97  (0.83-1.13) | 0.82  (0.75-0.90) |
| HR_Adj1_ (95% CI) | Ref. 1.0 | 0.91  (0.82-1.00) | 0.90  (0.80-1.01) | 0.92  (0.81-1.05) | 0.93  (0.86-1.00) | Ref. 1.0 | 0.97  (0.86-1.10) | 0.96  (0.83-1.11) | 1.00  (0.85-1.17) | 0.84  (0.76-0.92) |
| HR_Adj2_ (95% CI) | Ref. 1.0 | 0.91  (0.82-1.01) | 0.90  (0.80-1.01) | 0.92  (0.81-1.05) | 0.93  (0.86-1.01) | Ref. 1.0 | 0.98  (0.87-1.11) | 0.96  (0.83-1.12) | 1.00 (  0.86-1.18) | 0.84  (0.77-0.93) |
| HR_Adj3_ (95% CI) | Ref. 1.0 | 0.92  (0.83-1.01) | 0.91  (0.81-1.02) | 0.93  (0.81-1.06) | 0.94  (0.87-1.01) | Ref. 1.0 | 1.00  (0.88-1.13) | 0.98  (0.85-1.14) | 1.03  (0.88-1.20) | 0.87  (0.79-0.95) |
| **Girls** | | | | | | | | | | |
| Event (n) | 427 | 384 | 232 | 152 | 2827 | 145 | 118 | 65 | 53 | 809 |
| IR (95% CI) | 1.39  (1.26-1.52) | 1.28  (1.15-1.40) | 1.30  (1.13-1.47) | 1.24  (1.04-1.43) | 1.16  (1.11-1.20) | 1.10 (0.92-1.28) | 0.86  (0.70-1.01) | 0.82  (0.62-1.02) | 0.91  (0.66-1.15) | 0.83  (0.77-0.89) |
| HR_Cr_ (95% CI) | Ref. 1.0 | 0.91  (0.79-1.04) | 0.93  (0.79-1.09) | 0.88  (0.73-1.05) | 0.83  (0.75-0.92) | Ref. 1.0 | 0.78  (0.61-0.99) | 0.75  (0.56-1.00) | 0.82  (0.60-1.13) | 0.76  (0.63-0.90) |
| HR_Adj1_ (95% CI) | Ref. 1.0 | 0.94  (0.80-1.09) | 0.95  (0.80-1.14) | 0.91  (0.74-1.12) | 0.92  (0.82-1.03) | Ref. 1.0 | 0.84  (0.65-1.09) | 0.83  (0.61-1.13) | 0.86  (0.62-1.21) | 0.83  (0.68-1.00) |
| HR_Adj2_ (95% CI) | Ref. 1.0 | 0.95  (0.81-1.11) | 0.96  (0.81-1.15) | 0.92  (0.75-1.14) | 0.94  (0.84-1.05) | Ref. 1.0 | 0.86  (0.67-1.11) | 0.85  (0.62-1.15) | 0.88  (0.63-1.23) | 0.84  (0.70-1.02) |
| HR_Adj3_ (95% CI) | Ref. 1.0 | 0.95  (0.81-1.11) | 0.97  (0.81-1.16) | 0.93  (0.75-1.14) | 0.94  (0.84-1.05) | Ref. 1.0 | 0.88  (0.68-1.14) | 0.87  (0.64-1.18) | 0.90  (0.64-1.26) | 0.87  (0.72-1.05) |

HRs were estimated with Cox proportional hazards models. Crude (HR_cr_) and adjusted (HR_Adj_) models compare vacuum-assisted delivery (stratified by fetal head station: outlet, mid/low) and spontaneous vaginal delivery with emergency caesarean delivery as the reference category. HR_Adj1_: Adjusted for maternal age, smoking, maternal BMI, diabetes mellitus type 1 or 2, birthyear. HR_Adj2_: Adjusted for variables in model one and gestational diabetes, preeclampsia and chorioamnionitis. HR_Adj3_: Adjusted for variables of model 1 and 2 and additionally for maternal educational level and maternal pre-pregnancy comorbidities (ADHD, ASD, depression, anxiety disorder). CI = confidence interval; ECD, Emergency cesarean delivery; IR = incidence rate (per 1 000 person-years); SVD, Spontaneous vaginal delivery

## **Table M.** **Incidence and hazard ratios of cerebral palsy** in children by mode of delivery, comparing vacuum-assisted delivery and spontaneous vaginal delivery with emergency caesarean delivery as reference, **stratified by birth cohort (children born between 1997 and 2006 and 2007 or later).**

|  | **Birth year 1997-2006** | | | | | **Birth year** ≥**2007** | | | | |
| --- | --- | --- | --- | --- | --- | --- | --- | --- | --- | --- |
|  |  | **Vacuum-assisted delivery** | | |  |  | **Vacuum-assisted delivery** | | |  |
|  | **ECD** | **Total** | **Outlet** | **Mid/Low** | **SVD** | **ECD** | **Total** | **Outlet** | **Mid/Low** | **SVD** |
| **All** | | | | | | | | | | |
| Event (n) | 128 | 105 | 47 | 58 | 360 | 79 | 54 | 32 | 22 | 216 |
| IR (95% CI) | 0.18  (0.15-0.21) | 0.15  (0.12-0.18) | 0.11  (0.08-0.15) | 0.20  (0.15-0.26) | 0.07  (0.07-0.08) | 0.26  (0.20-0.31) | 0.18  (0.13-0.22) | 0.18  (0.12-0.25) | 0.16  (0.10-0.23) | 0.11  (0.10-0.13) |
| HR_Cr_ (95% CI) | Ref. 1.0 | 0.77  (0.56- 1.04) | 0.60  (0.40- 0.89) | 1.00  (0.69- 1.46) | 0.49  (0.39- 0.62) | Ref. 1.0 | 0.77  (0.51-1.17) | 0.89  (0.55-1.42) | 0.63  (0.35-1.13) | 0.51  (0.38-0.70) |
| HR_Adj1_ (95% CI) | Ref. 1.0 | 0.84  (0.61-1.17) | 0.66  (0.43-1.01) | 1.10  (0.74-1.63) | 0.51  (0.39-0.65) | Ref. 1.0 | 0.74  (0.48-1.13) | 0.88  (0.55-1.43) | 0.55  (0.29-1.03) | 0.55  (0.29-1.03) |
| HR_Adj2_ (95% CI) | Ref. 1.0 | 0.84  (0.61-1.17) | 0.66  (0.44-1.01) | 1.10  (0.74-1.64) | 0.51  (0.39-0.66) | Ref. 1.0 | 0.78  (0.51-1.20) | 0.94  (0.58-1.52) | 0.58  (0.31-1.09) | 0.54  (0.39-0.75) |
| HR_Adj3_ (95% CI) | Ref. 1.0 | 0.85  (0.61-1.18) | 0.67  (0.44-1.02) | 1.11  (0.74-1.64) | 0.51  (0.40-0.66) | Ref. 1.0 | 0.79  (0.52-1.21) | 0.95  (0.59-1.55) | 0.59  (0.31-1.11) | 0.55  (0.39-0.77) |
| **Boys** | | | | | | | | | | |
| Event (n) | 73 | 63 | 25 | 38 | 194 | 40 | 35 | 21 | 14 | 128 |
| IR (95% CI) | 0.18  (0.14-0.23) | 0.16  (0.12-0.20) | 0.11  (0.07-0.15) | 0.23  (0.16-0.31) | 0.08  (0.07-0.09) | 0.23  (0.16-0.30) | 0.21  (0.14-0.27) | 0.22  (0.13-0.31) | 0.19  (0.09-0.28) | 0.13  (0.11-0.16) |
| HR_Cr_ (95% CI) | Ref. 1.0 | 0.79  (0.53-1.17) | 0.52  (0.31-0.90) | 1.16  (0.73-1.83) | 0.46  (0.34-0.63) | Ref. 1.0 | 1.16  (0.68-1.99) | 1.33  (0.73-2.44) | 0.94  (0.45-1.96) | 0.72  (0.46-1.12) |
| HR_Adj1_ (95% CI) | Ref. 1.0 | 0.88  (0.58-1.33) | 0.62  (0.36-1.08) | 1.25  (0.77-2.03) | 0.49  (0.35-0.68) | Ref. 1.0 | 1.14  (0.66-1.97) | 1.29  (0.69-2.40) | 0.95  (0.45-1.98) | 0.70  (0.44-1.10) |
| HR_Adj2_ (95% CI) | Ref. 1.0 | 0.87  (0.58-1.33) | 0.62  (0.35-1.07) | 1.24  (0.76-2.02) | 0.48  (0.35-0.68) | Ref. 1.0 | 1.16  (0.67-2.01) | 1.32  (0.70-2.46) | 0.96  (0.46-2.01) | 0.71  (0.45-1.12) |
| HR_Adj3_ (95% CI) | Ref. 1.0 | 0.88  (0.58-1.34) | 0.62  (0.36-1.08) | 1.25  (0.77-2.04) | 0.49  (0.35-0.68) | Ref. 1.0 | 1.18  (0.68-2.05) | 1.34  (0.72-2.51) | 0.98  (0.47-2.05) | 0.72  (0.45-1.14) |
| **Girls** | | | | | | | | | | |
| Event (n) | 55 | 42 | 22 | 20 | 166 | 39 | 19 | 11 | 8 | 88 |
| IR (95% CI) | 0.18  (0.13-0.23) | 0.14  (0.10-0.18) | 0.12  (0.07-0.17) | 0.16  (0.09-0.23) | 0.07  (0.06-0.08) | 0.29  (0.20-0.39) | 0.14  (0.08-0.20) | 0.14  (0.06-0.22) | 0.14  (0.04-0.23) | 0.09  (0.07-0.11) |
| HR_Cr_ (95% CI) | Ref. 1.0 | 0.73  (0.44-1.20) | 0.71  (0.39-1.30) | 0.75  (0.38-1.47) | 0.53  (0.37-0.77) | Ref. 1.0 | 0.43  (0.22-0.85) | 0.50  (0.23-1.10) | 0.34  (0.12-0.97) | 0.35  (0.23-0.55) |
| HR_Adj1_ (95% CI) | Ref. 1.0 | 0.78  (0.46-1.32) | 0.72  (0.38-1.37) | 0.87  (0.44-1.71) | 0.55  (0.37-0.81) | Ref. 1.0 | 0.38  (0.18-0.78) | 0.52  (0.24-1.16) | 0.18  (0.04-0.75) | 0.35  (0.22-0.56) |
| HR_Adj2_ (95% CI) | Ref. 1.0 | 0.79  (0.47-1.34) | 0.73  (0.38-1.39) | 0.88  (0.44-1.74) | 0.56  (0.37-0.82) | Ref. 1.0 | 0.42  (0.20-0.88) | 0.59  (0.26-1.31) | 0.20  (0.05-0.84) | 0.40  (0.25-0.65) |
| HR_Adj3_ (95% CI) | Ref. 1.0 | 0.80  (0.47-1.35) | 0.74  (0.39-1.40) | 0.88  (0.45-1.75) | 0.56  (0.38-0.83) | Ref. 1.0 | 0.42  (0.20-0.89) | 0.59  (0.27-1.32) | 0.20  (0.05-0.84) | 0.41 (0.25-0.66) |

HRs were estimated with Cox proportional hazards models. Crude (HR_cr_) and adjusted (HR_Adj_) models compare vacuum-assisted delivery (stratified by fetal head station: outlet, mid/low) and spontaneous vaginal delivery with emergency caesarean delivery as the reference category. HR_Adj1_: Adjusted for maternal age, smoking, maternal BMI, diabetes mellitus type 1 or 2, birthyear. HR_Adj2_: Adjusted for variables in model one and gestational diabetes, preeclampsia and chorioamnionitis. HR_Adj3_: Adjusted for variables of model 1 and 2 and additionally for maternal educational level and maternal pre-pregnancy comorbidities (ADHD, ASD, depression, anxiety disorder). CI = confidence interval; ECD, Emergency cesarean delivery; IR = incidence rate (per 1 000 person-years); SVD, Spontaneous vaginal delivery

## **Table N. Incidence and hazard ratios of epilepsy** in children by mode of delivery, comparing vacuum-assisted delivery and spontaneous vaginal delivery with emergency caesarean delivery as reference, **stratified by birth cohort (children born between 1997 and 2006 and 2007 or later).**

|  | **Birth year 1997-2006** | | | | | **Birth year** ≥**2007** | | | | |
| --- | --- | --- | --- | --- | --- | --- | --- | --- | --- | --- |
|  |  | **Vacuum-assisted delivery** | | |  |  | **Vacuum-assisted delivery** | | |  |
|  | **ECD** | **Total** | **Outlet** | **Mid/Low** | **SVD** | **ECD** | **Total** | **Outlet** | **Mid/Low** | **SVD** |
| **All** | | | | | | | | | | |
| Event (n) | 478 | 404 | 230 | 174 | 2383 | 251 | 215 | 105 | 110 | 1256 |
| IR (95% CI) | 0.68  (0.62-0.74) | 0.59  (0.53-0.64) | 0.57  (0.49-0.64) | 0.61  (0.52-0.70) | 0.49  (0.47-0.51) | 0.82  (0.72-0.92) | 0.70  (0.61-0.79) | 0.60  (0.49-0.72) | 0.83  (0.67-0.98) | 0.65  (0.61-0.69) |
| HR_Cr_ (95% CI) | Ref. 1.0 | 0.85  (0.74- 0.97) | 0.82  (0.69- 0.97) | 0.89  (0.74- 1.06) | 0.75  (0.67- 0.83) | Ref. 1.0 | 0.89  (0.73-1.08) | 0.75  (0.58-0.96) | 1.07  (0.84-1.37) | 0.84  (0.72-0.97) |
| HR_Adj1_ (95% CI) | Ref. 1.0 | 0.85  (0.73- 0.99) | 0.82  (0.69- 0.98) | 0.89  (0.73- 1.09) | 0.75  (0.67- 0.84) | Ref. 1.0 | 0.93  (0.75-1.15) | 0.79  (0.61-1.03) | 1.11  (0.86-1.43) | 0.91  (0.78-1.07) |
| HR_Adj2_ (95% CI) | Ref. 1.0 | 0.85  (0.73- 0.99) | 0.82  (0.68- 0.98) | 0.89  (0.73- 1.09) | 0.75  (0.67- 0.84) | Ref. 1.0 | 0.94  (0.76-1.16) | 0.80  (0.61-1.04) | 1.12  (0.86-1.44) | 0.92  (0.78-1.08) |
| HR_Adj3_ (95% CI) | Ref. 1.0 | 0.85  (0.73- 0.99) | 0.82  (0.69- 0.98) | 0.90  (0.73- 1.09) | 0.75  (0.67- 0.84) | Ref. 1.0 | 0.94 (0.76-1.17) | 0.80  (0.62-1.05) | 1.12  (0.87-1.45) | 0.92  (0.79-1.09) |
| **Boys** | | | | | | | | | | |
| Event (n) | 252 | 251 | 132 | 119 | 1240 | 154 | 139 | 69 | 70 | 652 |
| IR (95% CI) | 0.64  (0.56-0.72) | 0.65  (0.57-0.73) | 0.58  (0.48-0.68) | 0.74  (0.60-0.87) | 0.52  (0.49-0.55) | 0.88  (0.74-1.02) | 0.82  (0.68-0.95) | 0.73  (0.55-0.90) | 0.94  (0.72-1.16) | 0.68  (0.63-0.73) |
| HR_Cr_ (95% CI) | Ref. 1.0 | 0.99  (0.82-1.19) | 0.88  (0.70-1.09) | 1.15  (0.92-1.44) | 0.82  (0.71-0.95) | Ref. 1.0 | 0.98  (0.76-1.26) | 0.87  (0.64-1.19) | 1.11  (0.81-1.52) | 0.84  (0.69-1.02) |
| HR_Adj1_ (95% CI) | Ref. 1.0 | 1.02  (0.83-1.24) | 0.91  (0.72-1.16) | 1.16  (0.91-1.48) | 0.84  (0.72-0.98) | Ref. 1.0 | 1.04  (0.80-1.36) | 0.95  (0.69-1.32) | 1.15  (0.83-1.60) | 0.91  (0.74-1.13) |
| HR_Adj2_ (95% CI) | Ref. 1.0 | 1.01  (0.83-1.23) | 0.91  (0.72-1.15) | 1.16  (0.91-1.48) | 0.84  (0.72-0.98) | Ref. 1.0 | 1.04  (0.80-1.36) | 0.95  (0.69-1.32) | 1.16  (0.83-1.61) | 0.92  (0.74-1.13) |
| HR_Adj3_ (95% CI) | Ref. 1.0 | 1.02  (0.84-1.24) | 0.91  (0.72-1.16) | 1.16  (0.91-1.48) | 0.84  (0.72-0.98) | Ref. 1.0 | 1.05  (0.81-1.38) | 0.96  (0.69-1.33) | 1.17  (0.84-1.62) | 0.92  (0.75-1.14) |
| **Girls** | | | | | | | | | | |
| Event (n) | 226 | 153 | 98 | 55 | 1143 | 97 | 76 | 36 | 40 | 604 |
| IR (95% CI) | 0.74  (0.64-0.83) | 0.51  (0.43-0.59) | 0.55  (0.44-0.66) | 0.45  (0.33-0.56) | 0.47  (0.44-0.49) | 0.73  (0.59-0.88) | 0.55  (0.43-0.68) | 0.46  (0.31-0.60) | 0.69  (0.47-0.90) | 0.62  (0.57-0.67) |
| HR_Cr_ (95% CI) | Ref. 1.0 | 0.69  (0.55-0.85) | 0.76  (0.59-0.97) | 0.58  (0.43-0.80) | 0.67  (0.57-0.78) | Ref. 1.0 | 0.77  (0.56-1.06) | 0.59  (0.38-0.89) | 1.01  (0.69-1.49) | 0.85  (0.68-1.08) |
| HR_Adj1_ (95% CI) | Ref. 1.0 | 0.66  (0.52-0.84) | 0.71  (0.54-0.94) | 0.58  (0.41-0.82) | 0.66  (0.56-0.78) | Ref. 1.0 | 0.78  (0.55-1.09) | 0.58  (0.37-0.92) | 1.03  (0.69-1.56) | 0.92  (0.72-1.18) |
| HR_Adj2_ (95% CI) | Ref. 1.0 | 0.66  (0.52-0.84) | 0.71  (0.54-0.94) | 0.58  (0.41-0.82) | 0.66  (0.56-0.78) | Ref. 1.0 | 0.78  (0.56-1.11) | 0.59  (0.37-0.93) | 1.05  (0.69-1.58) | 0.94  (0.73-1.20) |
| HR_Adj3_ (95% CI) | Ref. 1.0 | 0.66  (0.52-0.84) | 0.72  (0.54-0.94) | 0.58  (0.41-0.82) | 0.66  (0.56-0.78) | Ref. 1.0 | 0.79  (0.56-1.11) | 0.59  (0.38-0.93) | 1.05  (0.70-1.58) | 0.94  (0.73-1.20) |

HRs were estimated with Cox proportional hazards models. Crude (HR_cr_) and adjusted (HR_Adj_) models compare vacuum-assisted delivery (stratified by fetal head station: outlet, mid/low) and spontaneous vaginal delivery with emergency caesarean delivery as the reference category. HR_Adj1_: Adjusted for maternal age, smoking, maternal BMI, diabetes mellitus type 1 or 2, birthyear. HR_Adj2_: Adjusted for variables in model one and gestational diabetes, preeclampsia and chorioamnionitis. HR_Adj3_: Adjusted for variables of model 1 and 2 and additionally for maternal educational level and maternal pre-pregnancy comorbidities (ADHD, ASD, depression, anxiety disorder). CI = confidence interval; ECD, Emergency cesarean delivery; IR = incidence rate (per 1 000 person-years); SVD, Spontaneous vaginal delivery

## **Table O. Incidence and hazard ratios of intellectual disability** in children by mode of delivery, comparing vacuum-assisted delivery and spontaneous vaginal delivery with emergency caesarean delivery as reference, **stratified by birth cohort (children born between 1997 and 2006 and 2007 or later).**

|  | **Birth year 1997-2006** | | | | | **Birth year** ≥**2007** | | | | |
| --- | --- | --- | --- | --- | --- | --- | --- | --- | --- | --- |
|  |  | **Vacuum-assisted delivery** | | |  |  | **Vacuum-assisted delivery** | | |  |
|  | **ECD** | **Total** | **Outlet** | **Mid/Low** | **SVD** | **ECD** | **Total** | **Outlet** | **Mid/Low** | **SVD** |
| **All** | | | | | | | | | | |
| Event (n) | 380 | 274 | 165 | 109 | 1779 | 219 | 172 | 101 | 71 | 920 |
| IR (95% CI) | 0.51  (0.46-0.56) | 0.37  (0.33-0.42) | 0.38  (0.33-0.44) | 0.36  (0.29-0.43) | 0.35  (0.33-0.36) | 0.64  (0.56- 0.73) | 0.50  (0.43- 0.58) | 0.52  (0.42- 0.63) | 0.48  (0.37- 0.59) | 0.43  (0.40- 0.46) |
| HR_Cr_ (95% CI) | Ref. 1.0 | 0.73  (0.62-0.85) | 0.75  (0.62-0.90) | 0.70  (0.56-0.86) | 0.69  (0.62-0.77) | Ref. 1.0 | 0.79  (0.64- 0.96) | 0.81  (0.64- 1.03) | 0.75  (0.57- 0.98) | 0.67  (0.58- 0.77) |
| HR_Adj1_ (95% CI) | Ref. 1.0 | 0.78  (0.66-0.93) | 0.80  (0.66-0.98) | 0.76  (0.60-0.95) | 0.68  (0.61-0.78) | Ref. 1.0 | 0.83  (0.68- 1.02) | 0.87  (0.68- 1.11) | 0.79  (0.59- 1.04) | 0.69  (0.59- 0.81) |
| HR_Adj2_ (95% CI) | Ref. 1.0 | 0.79  (0.67-0.94) | 0.81  (0.67-0.99) | 0.77  (0.61-0.97) | 0.70  (0.61-0.79) | Ref. 1.0 | 0.84  (0.68- 1.04) | 0.88  (0.69- 1.12) | 0.79  (0.60- 1.05) | 0.70  (0.60- 0.82) |
| HR_Adj3_ (95% CI) | Ref. 1.0 | 0.81  (0.68-0.96) | 0.83  (0.68-1.01) | 0.78  (0.62-0.98) | 0.71  (0.63-0.81) | Ref. 1.0 | 0.89  (0.72- 1.09) | 0.92  (0.72- 1.18) | 0.84  (0.63- 1.11) | 0.74 (0.63- 0.87) |
| **Boys** | | | | | | | | | | |
| Event (n) | 246 | 163 | 92 | 71 | 1072 | 145 | 116 | 75 | 41 | 625 |
| IR (95% CI) | 0.59  (0.52-0.66) | 0.40  (0.33-0.46) | 0.38  (0.30-0.46) | 0.41  (0.32-0.51) | 0.42  (0.40-0.45) | 0.75  (0.63- 0.87) | 0.62  (0.50- 0.73) | 0.71  (0.55- 0.87) | 0.49  (0.34- 0.65) | 0.59  (0.54- 0.63) |
| HR_Cr_ (95% CI) | Ref. 1.0 | 0.67  (0.55-0.81) | 0.64  (0.51-0.82) | 0.69  (0.53-0.90) | 0.7  3 (0.64-0.84) | Ref. 1.0 | 0.82  (0.65- 1.05) | 0.95  (0.72- 1.26) | 0.66  (0.47- 0.94) | 0.79  (0.66- 0.94) |
| HR_Adj1_ (95% CI) | Ref. 1.0 | 0.73  (0.59-0.91) | 0.70  (0.54-0.91) | 0.77  (0.58-1.03) | 0.74  (0.64-0.87) | Ref. 1.0 | 0.88  (0.69- 1.14) | 1.01  (0.76- 1.35) | 0.72  (0.50- 1.02) | 0.81  (0.67- 0.98) |
| HR_Adj2_ (95% CI) | Ref. 1.0 | 0.74  (0.60-0.92) | 0.71  (0.55-0.92) | 0.79  (0.59-1.05) | 0.76  (0.65-0.89) | Ref. 1.0 | 0.89  (0.69- 1.15) | 1.02  (0.76- 1.36) | 0.72  (0.51- 1.03) | 0.82  (0.68- 0.99) |
| HR_Adj3_ (95% CI) | Ref. 1.0 | 0.76  (0.61-0.94) | 0.73  (0.56-0.95) | 0.80  (0.60-1.07) | 0.78  (0.67-0.91) | Ref. 1.0 | 0.95  (0.74- 1.23) | 1.08  (0.81- 1.45) | 0.78  (0.55- 1.12) | 0.88  (0.72- 1.06) |
| **Girls** | | | | | | | | | | |
| Event (n) | 134 | 111 | 73 | 38 | 707 | 74 | 56 | 26 | 30 | 295 |
| IR (95% CI) | 0.41  (0.34- 0.48) | 0.35  (0.28- 0.41) | 0.39  (0.30- 0.47) | 0.29  (0.20- 0.38) | 0.27  (0.25- 0.29) | 0.50  (0.39- 0.62) | 0.37  (0.27- 0.46) | 0.30  (0.18- 0.41) | 0.46  (0.30- 0.63) | 0.27  (0.24- 0.30) |
| HR_Cr_ (95% CI) | Ref. 1.0 | 0.83  (0.65- 1.07) | 0.93  (0.70- 1.24) | 0.69  (0.48- 1.00) | 0.67  (0.56- 0.81) | Ref. 1.0 | 0.73  (0.52- 1.03) | 0.59  (0.38- 0.92) | 0.92  (0.60- 1.41) | 0.54  (0.42- 0.70) |
| HR_Adj1_ (95% CI) | Ref. 1.0 | 0.87  (0.66- 1.15) | 0.98  (0.72- 1.33) | 0.72  (0.48- 1.07) | 0.64  (0.52- 0.78) | Ref. 1.0 | 0.76  (0.53- 1.09) | 0.63  (0.40- 1.01) | 0.93  (0.59- 1.46) | 0.58  (0.44- 0.76) |
| HR_Adj2_ (95% CI) | Ref. 1.0 | 0.88  (0.67- 1.16) | 0.98  (0.72- 1.34) | 0.72  (0.49- 1.08) | 0.64  (0.52- 0.79) | Ref. 1.0 | 0.76  (0.53- 1.10) | 0.64  (0.40- 1.02) | 0.93  (0.59- 1.47) | 0.59  (0.45- 0.77) |
| HR_Adj3_ (95% CI) | Ref. 1.0 | 0.89  (0.68- 1.17) | 1.00  (0.73- 1.36) | 0.73  (0.49- 1.09) | 0.65  (0.53- 0.80) | Ref. 1.0 | 0.79  (0.54- 1.14) | 0.66  (0.41- 1.06) | 0.96  (0.61- 1.51) | 0.61  (0.46- 0.80) |

HRs were estimated with Cox proportional hazards models. Crude (HR_cr_) and adjusted (HR_Adj_) models compare vacuum-assisted delivery (stratified by fetal head station: outlet, mid/low) and spontaneous vaginal delivery with emergency caesarean delivery as the reference category. HR_Adj1_: Adjusted for maternal age, smoking, maternal BMI, diabetes mellitus type 1 or 2, birthyear. HR_Adj2_: Adjusted for variables in model one and gestational diabetes, preeclampsia and chorioamnionitis. HR_Adj3_: Adjusted for variables of model 1 and 2 and additionally for maternal educational level and maternal pre-pregnancy comorbidities (ADHD, ASD, depression, anxiety disorder). CI = confidence interval; ECD, Emergency cesarean delivery; IR = incidence rate (per 1 000 person-years); SVD, Spontaneous vaginal delivery

## **Table P. Incidence and hazard ratios of attention-deficit/hyperactivity disorder** in children by mode of delivery, comparing vacuum-assisted delivery and spontaneous vaginal delivery with emergency caesarean delivery as reference, **stratified by estimated relative fetal size.**

|  | **Appropriate for gestational age** | | | | | **Small for gestational age** | | | | | **Large for gestational age** | | | | |
| --- | --- | --- | --- | --- | --- | --- | --- | --- | --- | --- | --- | --- | --- | --- | --- |
|  |  | **Vacuum-assisted delivery** | | |  |  | **Vacuum-assisted delivery** | | |  |  | **Vacuum-assisted delivery** | | |  |
|  | **ECD** | **Total** | **Outlet** | **Mid/Low** | **SVD** | **ECD** | **Total** | **Outlet** | **Mid/Low** | **SVD** | **ECD** | **Total** | **Outlet** | **Mid/Low** | **SVD** |
| **All** | | | | | | | | | | | | | | | |
| Event (n) | 4732 | 4583 | 2601 | 1982 | 30 374 | 297 | 169 | 104 | 65 | 1172 | 312 | 87 | 33 | 54 | 500 |
| IR  (95% CI) | 5.33 (5.17- 5.48) | 4.95 (4.80- 5.09) | 4.83 (4.64- 5.01) | 5.12 (4.89- 5.34) | 4.79 (4.74- 4.85) | 7.06 (6.26- 7.87) | 5.96 (5.06- 6.86) | 5.79 (4.68- 6.90) | 6.27 (4.74- 7.79) | 6.23 (5.87- 6.59) | 5.89 (5.24-6.55) | 4.18 (3.30-5.06) | 3.16 (2.08-4.24) | 5.21 (3.82-6.59) | 5.26 (4.80-5.72) |
| HR_Cr_  (95% CI) | Ref. 1.0 | 0.92 (0.88- 0.96) | 0.90 (0.85- 0.94) | 0.95 (0.90-1.00) | 0.90 (0.87- 0.93) | Ref. 1.0 | 0.85 (0.70- 1.02) | 0.82 (0.66- 1.03) | 0.89 (0.68- 1.16) | 0.88 (0.78- 1.00) | Ref. 1.0 | 0.69 (0.55- 0.88) | 0.52 (0.37- 0.75) | 0.87 (0.65- 1.16) | 0.89 (0.77- 1.03) |
| HR_Adj1_ (95% CI) | Ref. 1.0 | 0.94 (0.90- 0.98) | 0.91 (0.86- 0.96) | 0.98 (0.93- 1.04) | 0.87 (0.85- 0.90) | Ref. 1.0 | 0.87 (0.71- 1.06) | 0.82 (0.64- 1.04) | 0.97 (0.73- 1.28) | 0.83 (0.72- 0.95) | Ref. 1.0 | 0.71 (0.55-0.92) | 0.46 (0.30- 0.69) | 0.97 (0.71- 1.31) | 0.84 (0.72- 0.99) |
| HR_Adj2_ (95% CI) | Ref. 1.0 | 0.94 (0.90- 0.99) | 0.91 (0.87- 0.96) | 0.99 (0.93- 1.05) | 0.88 (0.85- 0.91) | Ref. 1.0 | 0.86 (0.70- 1.06) | 0.81 (0.64- 1.03) | 0.96 (0.72- 1.27) | 0.82 (0.72- 0.95) | Ref. 1.0 | 0.70 (0.54-0.91) | 0.46 (0.30- 0.69) | 0.97 (0.71- 1.31) | 0.84 (0.72- 0.99) |
| HR_Adj3_ (95% CI) | Ref. 1.0 | 0.96 (0.91-1.00) | 0.93 (0.88- 0.98) | 1.00 (0.94- 1.06) | 0.89 (0.86- 0.92) | Ref. 1.0 | 0.88 (0.72- 1.08) | 0.82 (0.65- 1.05) | 0.98 (0.74- 1.31) | 0.83 (0.72- 0.95) | Ref. 1.0 | 0.71 (0.55-0.92) | 0.45 (0.30-0.68) | 0.99 (0.73-1.35) | 0.85 (0.72-1.00) |
| **Boys** | | | | | | | | | | | | | | | |
| Event (n) | 3345 | 3273 | 1836 | 1437 | 19 643 | 205 | 111 | 65 | 46 | 754 | 198 | 58 | 24 | 34 | 289 |
| IR  (95% CI) | 6.68 (6.45- 6.91) | 6.35 (6.13- 6.56) | 6.17 (5.89- 6.45) | 6.59 (6.25- 6.93) | 6.32 (6.24- 6.41) | 9.09 (7.84-10.33) | 7.17 (5.84-8.51) | 6.61 (5.00-8.22) | 8.16 (5.80-10.52) | 8.15 (7.56-8.72) | 7.37 (6.34-8.39) | 5.86 (4.35-7.36) | 4.69 (2.81-6.56) | 7.11 (4.72-9.50) | 6.99 (6.19-7.80) |
| HR_Cr_  (95% CI) | Ref. 1.0 | 0.94 (0.89- 0.99) | 0.91 (0.86- 0.97) | 0.97 (0.92- 1.04) | 0.95 (0.92- 0.99) | Ref. 1.0 | 0.78 (0.62- 0.98) | 0.72 (0.55- 0.95) | 0.88 (0.64- 1.22) | 0.90 (0.77- 1.04) | Ref. 1.0 | 0.78 (0.58-1.04) | 0.62 (0.40-0.94) | 0.95 (0.66-1.36) | 0.95 (0.80-1.14) |
| HR_Adj1_ (95% CI) | Ref. 1.0 | 0.96 (0.91- 1.01) | 0.93 (0.87- 0.99) | 0.99 (0.93- 1.06) | 0.92 (0.89- 0.96) | Ref. 1.0 | 0.74 (0.58- 0.95) | 0.67 (0.50-0.90) | 0.88 (0.63-1.23) | 0.80 (0.68- 0.94) | Ref. 1.0 | 0.78 (0.57-1.08) | 0.59 (0.37-0.95) | 1.00 (0.67-1.47) | 0.90 (0.73-1.10) |
| HR_Adj2_ (95% CI) | Ref. 1.0 | 0.96 (0.91- 1.01) | 0.93 (0.88- 0.99) | 1.00 (0.93- 1.07) | 0.93 (0.89- 0.97) | Ref. 1.0 | 0.73 (0.57- 0.94) | 0.66 (0.49-0.88) | 0.87 (0.62-1.22) | 0.79 (0.67- 0.93) | Ref. 1.0 | 0.78 (0.56-1.07) | 0.58 (0.36-0.94) | 0.99 (0.67-1.46) | 0.90 (0.73-1.10) |
| HR_Adj3_ (95% CI) | Ref. 1.0 | 0.98 (0.93- 1.03) | 0.95 (0.89- 1.01) | 1.01 (0.95- 1.08) | 0.95 (0.91- 0.98) | Ref. 1.0 | 0.74 (0.58- 0.95) | 0.67 (0.50-0.90) | 0.88 (0.63-1.24) | 0.79 (0.67-0.93) | Ref. 1.0 | 0.79 (0.58-1.09) | 0.59 (0.37-0.95) | 1.01 (0.68-1.49) | 0.91 (0.74-1.12) |
| **Girls** | | | | | | | | | | | | | | | |
| Event (n) | 1387 | 1310 | 765 | 545 | 10 731 | 92 | 58 | 39 | 19 | 418 | 114 | 29 | 9 | 20 | 211 |
| IR  (95% CI) | 3.58 (3.39- 3.76) | 3.19 (3.02- 3.36) | 3.17 (2.95- 3.40) | 3.22 (2.95- 3.49) | 3.32 (3.26- 3.38) | 4.72 (3.76-5.69) | 4.51 (3.35-5.67) | 4.80 (3.29-6.30) | 4.01 (2.21-5.82) | 4.38 (3.96-4.79) | 4.37 (3.57-5.18) | 2.66 (1.69-3.63) | 1.69 (0.59-2.80) | 3.58 (2.01-5.15) | 3.93 (3.40-4.46) |
| HR_Cr_  (95% CI) | Ref. 1.0 | 0.89 (0.82- 0.96) | 0.88 (0.80- 0.96) | 0.90 (0.81- 0.99) | 0.92 (0.87- 0.97) | Ref. 1.0 | 0.97 (0.70-1.34) | 1.03 (0.71-1.50) | 0.86 (0.53-1.41) | 0.94 (0.75-1.17) | Ref. 1.0 | 0.60 (0.40-0.90) | 0.39 (0.20-0.76) | 0.80 (0.50-1.29) | 0.89 (0.71-1.12) |
| HR_Adj1_ (95% CI) | Ref. 1.0 | 0.92 (0.85-1.00) | 0.89 (0.81- 0.98) | 0.96 (0.87- 1.07) | 0.91 (0.85- 0.97) | Ref. 1.0 | 1.15 (0.81-1.64) | 1.17 (0.78-1.74) | 1.12 (0.67-1.88) | 0.99 (0.76-1.27) | Ref. 1.0 | 0.63 (0.41-0.97) | 0.28 (0.12-0.65) | 0.98 (0.61-1.59) | 0.85 (0.66-1.10) |
| HR_Adj2_ (95% CI) | Ref. 1.0 | 0.92 (0.85-1.00) | 0.89 (0.81- 0.98) | 0.96 (0.86- 1.07) | 0.91 (0.85- 0.97) | Ref. 1.0 | 1.15 (0.81-1.64) | 1.16 (0.78-1.74) | 1.13 (0.67-1.90) | 0.99 (0.76-1.27) | Ref. 1.0 | 0.63 (0.41-0.98) | 0.28 (0.12-0.65) | 1.00 (0.61-1.62) | 0.86 (0.66-1.11) |
| HR_Adj3_ (95% CI) | Ref. 1.0 | 0.93 (0.85- 1.01) | 0.90 (0.82- 0.99) | 0.97 (0.87- 1.08) | 0.92 (0.86- 0.98) | Ref. 1.0 | 1.19 (0.83-1.70) | 1.19 (0.80-1.78) | 1.18 (0.71-1.99) | 1.00 (0.77-1.29) | Ref. 1.0 | 0.63 (0.41-0.98) | 0.28 (0.12-0.63) | 1.03 (0.63-1.67) | 0.87 (0.67-1.12) |
| Estimated relative fetal size classified according to Swedish national reference standards: small for gestational age (SGA) defined as <–2 SD from the mean, appropriate for gestational age (AGA) as within ±2 SD, and large for gestational age (LGA) as >+2 SD. HRs were estimated with Cox proportional hazards models. Crude (HR_cr_) and adjusted (HR_Adj_) models compare vacuum-assisted delivery (stratified by fetal head station: outlet, mid/low) and spontaneous vaginal delivery with emergency caesarean delivery as the reference category. HR_Adj1_: Adjusted for maternal age, smoking, maternal BMI, diabetes mellitus type 1 or 2, birthyear. HR_Adj2_: Adjusted for variables in model one and gestational diabetes, preeclampsia and chorioamnionitis. HR_Adj3_: Adjusted for variables of model 1 and 2 and additionally for maternal educational level and maternal pre-pregnancy comorbidities (ADHD, ASD, depression, anxiety disorder). CI = confidence interval; ECD, Emergency cesarean delivery; IR = incidence rate (per 1 000 person-years); SVD, Spontaneous vaginal delivery | | | | | | | | | | | | | | | |

## **Table Q. Incidence and hazard ratios of autism spectrum disorder** in children by mode of delivery, comparing vacuum-assisted delivery and spontaneous vaginal delivery with emergency caesarean delivery as reference, **stratified by estimated relative fetal size.**

|  | **Appropriate for gestational age** | | | | | **Small for gestational age** | | | | | **Large for gestational age** | | | | |
| --- | --- | --- | --- | --- | --- | --- | --- | --- | --- | --- | --- | --- | --- | --- | --- |
|  |  | **Vacuum-assisted delivery** | | |  |  | **Vacuum-assisted delivery** | | |  |  | **Vacuum-assisted delivery** | | |  |
|  | **ECD** | **Total** | **Outlet** | **Mid/Low** | **SVD** | **ECD** | **Total** | **Outlet** | **Mid/Low** | **SVD** | **ECD** | **Total** | **Outlet** | **Mid/Low** | **SVD** |
| **All** | | | | | | | | | | | | | | | |
| Event (n) | 1878 | 1739 | 982 | 757 | 10 657 | 140 | 83 | 51 | 32 | 419 | 114 | 50 | 21 | 29 | 200 |
| IR  (95% CI) | 2.08 (1.99- 2.18) | 1.85 (1.76- 1.94) | 1.80 (1.68- 1.91) | 1.93 (1.79- 2.06) | 1.66 (1.62- 1.69) | 3.26 (2.72- 3.80) | 2.89 (2.27- 3.51) | 2.81 (2.03- 3.58) | 3.03 (1.98- 4.08) | 2.18 (1.97- 2.39) | 2.11 (1.72-2.50) | 2.38 (1.72-3.04) | 2.00 (1.15-2.86) | 2.76 (1.76-3.76) | 2.07 (1.78-2.35) |
| HR_Cr_  (95% CI) | Ref. 1.0 | 0.88 (0.83- 0.94) | 0.86 (0.79- 0.92) | 0.92 (0.84-1.00) | 0.80 (0.76- 0.84) | Ref. 1.0 | 0.88 (0.67- 1.15) | 0.86 (0.62- 1.18) | 0.92 (0.62- 1.34) | 0.67 (0.55- 0.81) | Ref. 1.0 | 1.11 (0.80- 1.55) | 0.93 (0.59- 1.49) | 1.29 (0.86- 1.94) | 0.98 (0.78- 1.24) |
| HR_Adj1_ (95% CI) | Ref. 1.0 | 0.92 (0.86- 0.99) | 0.92 (0.85-1.00) | 0.93 (0.85- 1.02) | 0.85 (0.81- 0.90) | Ref. 1.0 | 0.88 (0.66- 1.18) | 0.81 (0.57- 1.15) | 1.01 (0.67- 1.50) | 0.69 (0.56- 0.85) | Ref. 1.0 | 1.26 (0.88-1.80) | 0.89 (0.52- 1.51) | 1.63 (1.08- 2.48) | 1.09 (0.84- 1.40) |
| HR_Adj2_ (95% CI) | Ref. 1.0 | 0.93 (0.87-1.00) | 0.93 (0.85- 1.01) | 0.93 (0.85- 1.02) | 0.86 (0.81- 0.91) | Ref. 1.0 | 0.89 (0.66- 1.19) | 0.81 (0.57- 1.16) | 1.01 (0.68- 1.51) | 0.70 (0.56- 0.86) | Ref. 1.0 | 1.27 (0.89-1.82) | 0.90 (0.53-1.54) | 1.64 (1.08-2.49) | 1.11 (0.86-1.44) |
| HR_Adj3_ (95% CI) | Ref. 1.0 | 0.94 (0.87-1.01) | 0.94 (0.86- 1.02) | 0.94 (0.86- 1.03) | 0.87 (0.82- 0.92) | Ref. 1.0 | 0.89 (0.67- 1.20) | 0.82 (0.58- 1.17) | 1.02 (0.68- 1.52) | 0.69 (0.56- 0.86) | Ref. 1.0 | 1.28 (0.90-1.84) | 0.88 (0.52-1.50) | 1.71 (1.13-2.60) | 1.12 (0.87-1.45) |
| **Boys** | | | | | | | | | | | | | | | |
| Event (n) | 1365 | 1290 | 717 | 573 | 7238 | 113 | 55 | 31 | 24 | 297 | 84 | 26 | 9 | 17 | 111 |
| IR (95% CI) | 2.67 (2.53- 2.81) | 2.45 (2.32- 2.59) | 2.36 (2.19- 2.54) | 2.58 (2.37- 2.79) | 2.28 (2.23- 2.33) | 4.88 (3.98-5.78) | 3.49 (2.57-4.41) | 3.10 (2.01-4.19) | 4.16 (2.50-5.83) | 3.12 (2.76-3.47) | 3.05 (2.40-3.70) | 2.58 (1.59-3.57) | 1.73 (0.60-2.87) | 3.48 (1.83-5.14) | 2.62 (2.13-3.11) |
| HR_Cr_ (95% CI) | Ref. 1.0 | 0.91 (0.84- 0.98) | 0.88 (0.80- 0.96) | 0.95 (0.87- 1.05) | 0.86 (0.81-0.91) | Ref. 1.0 | 0.70 (0.51- 0.97) | 0.63 (0.42- 0.93) | 0.83 (0.53- 1.29) | 0.64 (0.51- 0.79) | Ref. 1.0 | 0.83 (0.54-1.30) | 0.56 (0.28-1.11) | 1.13 (0.67-1.90) | 0.87 (0.66-1.16) |
| HR_Adj1_ (95% CI) | Ref. 1.0 | 0.96 (0.88- 1.04) | 0.95 (0.87- 1.05) | 0.96 (0.87- 1.07) | 0.91 (0.86- 0.97) | Ref. 1.0 | 0.68 (0.48- 0.96) | 0.59 (0.38-0.90) | 0.85 (0.53-1.35) | 0.65 (0.51- 0.82) | Ref. 1.0 | 1.06 (0.67-1.69) | 0.67 (0.32-1.41) | 1.46 (0.85-2.48) | 1.03 (0.75-1.42) |
| HR_Adj2_ (95% CI) | Ref. 1.0 | 0.96 (0.89-1.04) | 0.96 (0.87-1.05) | 0.97 (0.87- 1.08) | 0.92 (0.86- 0.98) | Ref. 1.0 | 0.68 (0.48- 0.96) | 0.58 (0.38-0.90) | 0.84 (0.53-1.34) | 0.65 (0.51- 0.82) | Ref. 1.0 | 1.07 (0.68-1.70) | 0.68 (0.33-1.42) | 1.47 (0.86-2.51) | 1.06 (0.77-1.46) |
| HR_Adj3_ (95% CI) | Ref. 1.0 | 0.97 (0.89- 1.05) | 0.97 (0.98- 0.88) | 0.93 (0.87- 0.99) | 0.93 (0.87- 0.99) | Ref. 1.0 | 0.69 (0.49-0.97) | 0.60 (0.39-0.92) | 0.84 (0.53-1.33) | 0.65 (0.51-0.83) | Ref. 1.0 | 1.11 (0.70-1.77) | 0.69 (0.33-1.45) | 1.55 (0.90-2.64) | 1.08 (0.78- |
| **Girls** | | | | | | | | | | | | | | | |
| Event (n) | 513 | 449 | 265 | 184 | 3419 | 27 | 28 | 20 | 8 | 122 | 30 | 24 | 12 | 12 | 89 |
| IR (95% CI) | 1.31 (1.20- 1.42) | 1.08 (0.98- 1.19) | 1.09 (0.96- 1.22) | 1.08 (0.92- 1.23) | 1.05 (1.01- 1.08) | 1.36 (0.85-1.88) | 2.16 (1.36-2.96) | 2.45 (1.38-3.52) | 1.67 (0.51-2.83) | 1.26 (1.03-1.48) | 1.13 (0.73-1.54) | 2.20 (1.32-3.08) | 2.27 (0.99-3.55) | 2.13 (0.93-3.34) | 1.64 (1.30-1.98) |
| HR_Cr_ (95% CI) | Ref. 1.0 | 0.82 (0.73- 0.93) | 0.83 (0.71- 0.96) | 0.82 (0.69- 0.97) | 0.79 (0.72- 0.87) | Ref. 1.0 | 1.59 (0.94-2.71) | 1.81 (1.01-3.22) | 1.23 (0.56-2.71) | 0.93 (0.61-1.41) | Ref. 1.0 | 1.95 (1.14-3.33) | 2.03 (1.04-3.97) | 1.87 (0.96-3.65) | 1.43 (0.95-2.17) |
| HR_Adj1_ (95% CI) | Ref. 1.0 | 0.86 (0.75- 0.98) | 0.87 (0.74- 1.02) | 0.84 (0.69-1.01) | 0.87 (0.79- 0.97) | Ref. 1.0 | 1.75 (0.98-3.13) | 1.81 (0.95-3.45) | 1.65 (0.73-3.71) | 1.05 (0.66-1.67) | Ref. 1.0 | 1.94 (1.08-3.45) | 1.58 (0.72-3.49) | 2.27 (1.15-4.49) | 1.43 (0.92-2.23) |
| HR_Adj2_ (95% CI) | Ref. 1.0 | 0.87 (0.76-1.00) | 0.88 (0.75- 1.04) | 0.85 (0.70- 1.02) | 0.89 (0.80- 0.98) | Ref. 1.0 | 1.81 (1.01-3.24) | 1.84 (0.96-3.51) | 1.75 (0.78-3.96) | 1.09 (0.68-1.73) | Ref. 1.0 | 1.95 (1.09-3.48) | 1.61 (0.73-3.55) | 2.27 (1.15-4.47) | 1.45 (0.93-2.26) |
| HR_Adj3_ (95% CI) | Ref. 1.0 | 0.88 (0.76- 1.01) | 0.89 (0.76- 1.05) | 0.85 (0.71- 1.03) | 0.89 (0.81-0.99) | Ref. 1.0 | 1.79 (1.00-3.20) | 1.83 (0.96-3.49) | 1.71 (0.76-3.85) | 1.05 (0.66-1.67) | Ref. 1.0 | 1.99 (1.11-3.55) | 1.60 (0.72-3.55) | 2.36 (1.19-4.66) | 1.46 (0.94-2.29) |
| Estimated relative fetal size classified according to Swedish national reference standards: small for gestational age (SGA) defined as <–2 SD from the mean, appropriate for gestational age (AGA) as within ±2 SD, and large for gestational age (LGA) as >+2 SD. HRs were estimated with Cox proportional hazards models. Crude (HR_cr_) and adjusted (HR_Adj_) models compare vacuum-assisted delivery (stratified by fetal head station: outlet, mid/low) and spontaneous vaginal delivery with emergency caesarean delivery as the reference category. HR_Adj1_: Adjusted for maternal age, smoking, maternal BMI, diabetes mellitus type 1 or 2, birthyear. HR_Adj2_: Adjusted for variables in model one and gestational diabetes, preeclampsia and chorioamnionitis. HR_Adj3_: Adjusted for variables of model 1 and 2 and additionally for maternal educational level and maternal pre-pregnancy comorbidities (ADHD, ASD, depression, anxiety disorder). CI = confidence interval; ECD, Emergency cesarean delivery; IR = incidence rate (per 1 000 person-years); SVD, Spontaneous vaginal delivery | | | | | | | | | | | | | | | |

## **Table R. Incidence and hazard ratios of cerebral palsy** in children by mode of delivery, comparing vacuum-assisted delivery and spontaneous vaginal delivery with emergency caesarean delivery as reference, **stratified by estimated relative fetal size.**

|  | **Appropriate for gestational age** | | | | | **Small for gestational age** | | | | | **Large for gestational age** | | | | |
| --- | --- | --- | --- | --- | --- | --- | --- | --- | --- | --- | --- | --- | --- | --- | --- |
|  |  | **Vacuum-assisted delivery** | | |  |  | **Vacuum-assisted delivery** | | |  |  | **Vacuum-assisted delivery** | | |  |
|  | **ECD** | **Total** | **Outlet** | **Mid/Low** | **SVD** | **ECD** | **Total** | **Outlet** | **Mid/Low** | **SVD** | **ECD** | **Total** | **Outlet** | **Mid/Low** | **SVD** |
| **All** | | | | | | | | | | | | | | | |
| Event (n) | 162 | 145 | 73 | 72 | 524 | 35 | 5 | 3 | 2 | 37 | 6 | 7 | 2 | 5 | 10 |
| IR  (95% CI) | 0.18 (0.15- 0.20) | 0.15 (0.13- 0.18) | 0.13 (0.10- 0.16) | 0.18 (0.14- 0.22) | 0.08 (0.07- 0.09) | 0.81 (0.54- 1.07) | 0.17 (0.02- 0.32) | 0.16 (-0.02- 0.34) | 0.19 (-0.07- 0.45) | 0.19 (0.13- 0.25) | 0.11 (0.02- 0.20) | 0.33 (0.09- 0.58) | 0.19 (-0.07- 0.45) | 0.47 (0.06- 0.89) | 0.10 (0.04- 0.17) |
| HR_Cr_  (95% CI) | Ref. 1.0 | 0.87 (0.67- 1.14) | 0.80 (0.58- 1.11) | 0.97 (0.69- 1.36) | 0.55 (0.45- 0.68) | Ref. 1.0 | 0.22 (0.08- 0.62) | 0.17 (0.04- 0.72) | 0.30 (0.07- 1.25) | 0.27 (0.16- 0.45) | Ref. 1.0 | 2.06 (0.55- 7.66) | 2.08 (0.40- 10.70) | 2.04 (0.40- 10.50) | 1.04 (0.35- 3.11) |
| HR_Adj1_ (95% CI) | Ref. 1.0 | 0.92 (0.69- 1.21) | 0.86 (0.61- 1.20) | 1.00 (0.70- 1.43) | 0.56 (0.45-0.70) | Ref. 1.0 | 0.23 (0.08- 0.65) | 0.18 (0.04- 0.77) | 0.30 (0.07- 1.26) | 0.25 (0.14- 0.44) | Ref. 1.0 | 2.24 (0.59- 8.47) | 2.25 (0.43- 11.73) | 2.23 (0.43- 11.65) | 1.15 (0.37- 3.54) |
| HR_Adj2_ (95% CI) | Ref. 1.0 | 0.94 (0.71- 1.24) | 0.88 (0.62- 1.23) | 1.02 (0.71- 1.46) | 0.57 (0.46- 0.72) | Ref. 1.0 | 0.24 (0.08- 0.68) | 0.19 (0.05- 0.80) | 0.31 (0.07- 1.30) | 0.26 (0.15- 0.46) | Ref. 1.0 | 2.14 (0.57- 8.01) | 2.16 (0.42- 11.20) | 2.11 (0.41- 10.93) | 1.08 (0.36- 3.31) |
| HR_Adj3_ (95% CI) | Ref. 1.0 | 0.94 (0.71- 1.25) | 0.88 (0.63- 1.24) | 1.03 (0.72- 1.47) | 0.58 (0.46- 0.72) | Ref. 1.0 | 0.23 (0.08- 0.67) | 0.19 (0.04- 0.80) | 0.30 (0.07- 1.28) | 0.26 (0.15- 0.45) | Ref. 1.0 | 2.16 (0.57- 8.11) | 2.19 (0.42- 11.43) | 2.13 (0.41- 11.03) | 1.12 (0.37- 3.41) |
| **Boys** | | | | | | | | | | | | | | | |
| Event (n) | 85 | 92 | 44 | 48 | 295 | 20 | 2 | 0 | 2 | 20 | 6 | 2 | 1 | 1 | 6 |
| IR  (95% CI) | 0.16 (0.13- 0.20) | 0.17 (0.14- 0.21) | 0.14 (0.10- 0.19) | 0.21 (0.15- 0.27) | 0.09 (0.08- 0.10) | 0.85 (0.47-1.22) | 0.12 (-0.05-0.30) | 0 | 0.34 (-0.13-0.81) | 0.21 (0.12-0.30) | 0.21 (0.04- 0.39) | 0.20 (-0.08- 0.47) | 0.19 (-0.18- 0.57) | 0.20 (-0.19- 0.60) | 0.14 (0.03- 0.25) |
| HR_Cr_  (95% CI) | Ref. 1.0 | 1.12 (0.79- 1.59) | 0.98 (0.64- 1.49) | 1.31 (0.86-2.00) | 0.64 (0.48- 0.86) | Ref. 1.0 | 0.17 (0.04- 0.75) | 0.00 (0.00) | 0.47 (0.11- 2.06) | 0.26 (0.13- 0.51) | Ref. 1.0 | 1.11 (0.22- 5.73) | 1.12 (0.13- 9.54) | 1.11 (0.13- 9.49) | 0.68 (0.20- 2.34) |
| HR_Adj1_ (95% CI) | Ref. 1.0 | 1.22 (0.85- 1.76) | 1.09 (0.70- 1.68) | 1.40 (0.90- 2.19) | 0.65 (0.48- 0.89) | Ref. 1.0 | 0.18 (0.04- 0.80) | 0.00 (0.00) | 0.48 (0.11- 2.09) | 0.30 (0.15- 0.61) | Ref. 1.0 | 1.18 (0.23- 6.20) | 1.19 (0.14- 10.37) | 1.17 (0.14- 10.17) | 0.76 (0.21- 2.74) |
| HR_Adj2_ (95% CI) | Ref. 1.0 | 1.22 (0.84- 1.76) | 1.08 (0.70- 1.68) | 1.40 (0.90- 2.18) | 0.65 (0.48- 0.89) | Ref. 1.0 | 0.19 (0.04- 0.84) | 0.00 (0.00) | 0.50 (0.11- 2.18) | 0.31 (0.15- 0.64) | Ref. 1.0 | 1.14 (0.22- 5.92) | 1.16 (0.14- 9.98) | 1.12 (0.13- 9.70) | 0.73 (0.21- 2.61) |
| HR_Adj3_ (95% CI) | Ref. 1.0 | 1.23 (0.85- 1.77) | 1.09 (0.71- 1.70) | 1.41 (0.90- 2.20) | 0.66 (0.48- 0.90) | Ref. 1.0 | 0.18 (0.04- 0.79) | 0.00 (0.00) | 0.46 (0.11- 2.04) | 0.30 (0.15- 0.62) | Ref. 1.0 | 1.14 (0.22- 5.95) | 1.20 (0.14- 10.40) | 1.09 (0.13- 9.48) | 0.76 (0.21- 2.69) |
| **Girls** |  |  |  |  |  |  |  |  |  |  |  |  |  |  |  |
| Event (n) | 77 | 53 | 29 | 24 | 229 | 15 | 3 | 3 | 0 | 17 | 0 | 5 | 1 | 4 | 4 |
| IR (95% CI) | 0.20 (0.15-0.24) | 0.13 (0.09-0.16) | 0.12 (0.08-0.16) | 0.14 (0.08-0.20) | 0.07 (0.06-0.08) | 0.76 (0.37-1.14) | 0.23 (-0.03-0.49) | 0.36 (-0.05-0.77) | 0 | 0.17 (0.09-0.26) | 0 | 0.46 (0.06- 0.86) | 0.19 (-0.18- 0.55) | 0.71 (0.01- 1.41) | 0.07 (0.00- 0.14) |
| HR_Cr_ (95% CI) | Ref. 1.0 | 0.60 (0.39- 0.93) | 0.61 (0.36- 1.02) | 0.60 (0.33- 1.07) | 0.46 (0.34- 0.63) | Ref. 1.0 | 0.29 (0.06-1.33) | 0.46 (0.10-2.10) | 0.00 (0.00) | 0.28 (0.13-0.64) | Ref. 1.0 | N/A | N/A | N/A | N/A |
| HR_Adj1_ (95% CI) | Ref. 1.0 | 0.60 (0.38-0.94) | 0.62 (0.36-1.06) | 0.58 (0.31-1.08) | 0.47 (0.34-0.65) | Ref. 1.0 | 0.30 (0.07-1.36) | 0.47 (0.10-2.16) | 0.00 (0.00) | 0.21 (0.08-0.50) | Ref. 1.0 | N/A | N/A | N/A | N/A |
| HR_Adj2_ (95% CI) | Ref. 1.0 | 0.63 (0.40-1.00) | 0.65 (0.38-1.12) | 0.61 (0.32-1.14) | 0.50 (0.36- 0.70) | Ref. 1.0 | 0.31 (0.07-1.42) | 0.49 (0.11-2.24) | 0.00 (0.00) | 0.21 (0.09-0.53) | Ref. 1.0 | N/A | N/A | N/A | N/A |
| HR_Adj3_ (95% CI) | Ref. 1.0 | 0.64 (0.40- 1.00) | 0.66 (0.38-1.13) | 0.61 (0.32-1.15) | 0.51 (0.36- 0.70) | Ref. 1.0 | 0.31 (0.07-1.43) | 0.49 (0.11-2.24) | 0.00 (0.00) | 0.22 (0.09-0.53) | Ref. 1.0 | N/A | N/A | N/A | N/A |
| Estimated relative fetal size classified according to Swedish national reference standards: small for gestational age (SGA) defined as <–2 SD from the mean, appropriate for gestational age (AGA) as within ±2 SD, and large for gestational age (LGA) as >+2 SD. HRs were estimated with Cox proportional hazards models. Crude (HR_cr_) and adjusted (HR_Adj_) models compare vacuum-assisted delivery (stratified by fetal head station: outlet, mid/low) and spontaneous vaginal delivery with emergency caesarean delivery as the reference category. HR_Adj1_: Adjusted for maternal age, smoking, maternal BMI, diabetes mellitus type 1 or 2, birthyear. HR_Adj2_: Adjusted for variables in model one and gestational diabetes, preeclampsia and chorioamnionitis. HR_Adj3_: Adjusted for variables of model 1 and 2 and additionally for maternal educational level and maternal pre-pregnancy comorbidities (ADHD, ASD, depression, anxiety disorder). CI = confidence interval; ECD, Emergency cesarean delivery; IR = incidence rate (per 1 000 person-years); SVD, Spontaneous vaginal delivery | | | | | | | | | | | | | | | |

## **Table S. Incidence and hazard ratios of epilepsy** in children by mode of delivery, comparing vacuum-assisted delivery and spontaneous vaginal delivery with emergency caesarean delivery as reference, **stratified by estimated relative fetal size.**

|  | **Appropriate for gestational age** | | | | | **Small for gestational age** | | | | | **Large for gestational age** | | | | |
| --- | --- | --- | --- | --- | --- | --- | --- | --- | --- | --- | --- | --- | --- | --- | --- |
|  |  | **Vacuum-assisted delivery** | | |  |  | **Vacuum-assisted delivery** | | |  |  | **Vacuum-assisted delivery** | | |  |
|  | **ECD** | **Total** | **Outlet** | **Mid/Low** | **SVD** | **ECD** | **Total** | **Outlet** | **Mid/Low** | **SVD** | **ECD** | **Total** | **Outlet** | **Mid/Low** | **SVD** |
| **All** | | | | | | | | | | | | | | | |
| Event (n) | 629 | 575 | 316 | 259 | 3428 | 60 | 24 | 12 | 12 | 137 | 33 | 17 | 6 | 11 | 65 |
| IR  (95% CI) | 0.69 (0.64- 0.75) | 0.61 (0.56- 0.66) | 0.58 (0.51- 0.64) | 0.66 (0.58- 0.74) | 0.53 (0.51- 0.55) | 1.39 (1.04- 1.74) | 0.83 (0.50- 1.16) | 0.65 (0.28- 1.02) | 1.12 (0.49- 1.76) | 0.71 (0.59- 0.83) | 0.61 (0.40-0.81) | 0.81 (0.42-1.19) | 0.57 (0.11-1.02) | 1.04 (0.43-1.66) | 0.67 (0.51-0.83) |
| HR_Cr_  (95% CI) | Ref. 1.0 | 0.89 (0.78-1.00) | 0.83 (0.72- 0.96) | 0.96 (0.83- 1.12) | 0.80 (0.73- 0.88) | Ref. 1.0 | 0.63 (0.38- 1.02) | 0.47 (0.25- 0.91) | 0.89 (0.48- 1.66) | 0.51 (0.37- 0.71) | Ref. 1.0 | 1.07 (0.56- 2.04) | 0.99 (0.41- 2.37) | 1.14 (0.50- 2.60) | 1.09 (0.71- 1.68) |
| HR_Adj1_ (95% CI) | Ref. 1.0 | 0.91 (0.80- 1.03) | 0.86 (0.73-1.00) | 0.98 (0.83- 1.16) | 0.83 (0.75- 0.92) | Ref. 1.0 | 0.55 (0.32- 0.94) | 0.33 (0.15- 0.74) | 0.93 (0.48- 1.78) | 0.50 (0.35- 0.71) | Ref. 1.0 | 1.06 (0.52-2.15) | 0.97 (0.37- 2.52) | 1.16 (0.48- 2.82) | 1.22 (0.76- 1.96) |
| HR_Adj2_ (95% CI) | Ref. 1.0 | 0.91 (0.80- 1.03) | 0.85 (0.73-1.00) | 0.98 (0.83- 1.15) | 0.83 (0.75- 0.91) | Ref. 1.0 | 0.57 (0.33- 0.99) | 0.35 (0.16- 0.77) | 0.96 (0.50- 1.86) | 0.52 (0.37- 0.74) | Ref. 1.0 | 1.10 (0.54-2.23) | 1.00 (0.38- 2.60) | 1.20 (0.49- 2.92) | 1.26 (0.78- 2.04) |
| HR_Adj3_ (95% CI) | Ref. 1.0 | 0.91 (0.80- 1.03) | 0.86 (0.73-1.00) | 0.98 (0.83- 1.16) | 0.83 (0.75- 0.92) | Ref. 1.0 | 0.59 (0.34- 1.02) | 0.36 (0.16- 0.80) | 1.00 (0.51- 1.92) | 0.54 (0.38- 0.76) | Ref. 1.0 | 1.09 (0.54-2.23) | 1.00 (0.38- 2.61) | 1.19 (0.49- 2.91) | 1.26 (0.78- 2.04) |
| **Boys** | | | | | | | | | | | | | | | |
| Event (n) | 345 | 366 | 191 | 175 | 1784 | 35 | 12 | 5 | 7 | 70 | 23 | 10 | 4 | 6 | 33 |
| IR  (95% CI) | 0.67 (0.60-0.74) | 0.69 (0.62-0.76) | 0.62 (0.54-0.71) | 0.78 (0.66-0.90) | 0.56 (0.53-0.58) | 1.49 (0.99- 1.98) | 0.75 (0.32- 1.17) | 0.49 (0.06- 0.92) | 1.19 (0.31- 2.07) | 0.72 (0.55- 0.89) | 0.83 (0.49-1.16) | 0.99 (0.38-1.60) | 0.77 (0.02-1.52) | 1.22 (0.24-2.19) | 0.77 (0.51-1.03) |
| HR_Cr_  (95% CI) | Ref. 1.0 | 1.05 (0.89- 1.22) | 0.94 (0.78- 1.13) | 1.19 (0.98- 1.44) | 0.88 (0.78- 0.99) | Ref. 1.0 | 0.50 (0.25-1.00) | 0.29 (0.10- 0.82) | 0.87 (0.38- 1.97) | 0.50 (0.32- 0.76) | Ref. 1.0 | 1.04 (0.46-2.35) | 1.02 (0.35-2.98) | 1.06 (0.36-3.08) | 0.92 (0.53-1.62) |
| HR_Adj1_ (95% CI) | Ref. 1.0 | 1.10 (0.93- 1.30) | 1.02 (0.83-1.24) | 1.22 (0.99-1.50) | 0.92 (0.81- 1.05) | Ref. 1.0 | 0.50 (0.24- 1.03) | 0.24 (0.07- 0.78) | 0.94 (0.41- 2.14) | 0.51 (0.33- 0.80) | Ref. 1.0 | 0.99 (0.42-2.36) | 0.83 (0.25-2.82) | 1.15 (0.39-3.40) | 1.04 (0.57-1.89) |
| HR_Adj2_ (95% CI) | Ref. 1.0 | 1.10 (0.93-1.30) | 1.01 (0.83-1.23) | 1.21 (0.99-1.49) | 0.92 (0.80- 1.05) | Ref. 1.0 | 0.53 (0.26- 1.10) | 0.26 (0.08- 0.84) | 0.99 (0.43- 2.28) | 0.54 (0.34- 0.86) | Ref. 1.0 | 0.99 (0.41-2.35) | 0.83 (0.25-2.82) | 1.15 (0.39-3.39) | 1.04 (0.57-1.89) |
| HR_Adj3_ (95% CI) | Ref. 1.0 | 1.10 (0.93- 1.30) | 1.01 (0.83-1.24) | 1.22 (0.99-1.50) | 0.92 (0.80- 1.05) | Ref. 1.0 | 0.56 (0.27- 1.16) | 0.27 (0.08- 0.88) | 1.05 (0.46- 2.42) | 0.57 (0.36- 0.92) | Ref. 1.0 | 0.97 (0.41-2.33) | 0.82 (0.24-2.78) | 1.14 (0.38-3.35) | 1.03 (0.57-1.87) |
| **Girls** | | | | | | | | | | | | | | | |
| Event (n) | 284 | 209 | 125 | 84 | 1644 | 25 | 12 | 7 | 5 | 67 | 10 | 7 | 2 | 5 | 32 |
| IR  (95% CI) | 0.73 (0.64-0.81) | 0.50 (0.44-0.57) | 0.51 (0.42-0.60) | 0.49 (0.39-0.60) | 0.50 (0.48-0.53) | 1.27 (0.77- 1.76) | 0.92 (0.40- 1.44) | 0.85 (0.22- 1.48) | 1.05 (0.13- 1.97) | 0.69 (0.52- 0.85) | 0.38 (0.14-0.61) | 0.64 (0.17-1.11) | 0.37 (-0.14-0.89) | 0.89 (0.11-1.67) | 0.59 (0.38-0.79) |
| HR_Cr_  (95% CI) | Ref. 1.0 | 0.70 (0.56-0.84) | 0.70 (0.56-0.88) | 0.69 (0.53-0.89) | 0.72 (0.63-0.83) | Ref. 1.0 | 0.81 (0.40- 1.63) | 0.74 (0.32- 1.74) | 0.92 (0.35- 2.43) | 0.55 (0.34- 0.89) | Ref. 1.0 | 1.19 (0.41-3.48) | 0.98 (0.21-4.45) | 1.39 (0.38-5.06) | 1.49 (0.73-3.05) |
| HR_Adj1_ (95% CI) | Ref. 1.0 | 0.69 (0.56-0.84) | 0.69 (0.53-0.87) | 0.70 (0.53-0.87) | 0.74 (0.64-0.86) | Ref. 1.0 | 0.64 (0.28- 1.46) | 0.49 (0.17- 1.45) | 0.91 (0.31- 2.67) | 0.50 (0.29- 0.86) | Ref. 1.0 | 1.36 (0.40-4.67) | 1.34 (0.28-6.49) | 1.38 (0.29-6.67) | 1.84 (0.79-4.30) |
| HR_Adj2_ (95% CI) | Ref. 1.0 | 0.69 (0.56-0.84) | 0.68 (0.53-0.86) | 0.67 (0.53-0.92) | 0.74 (0.64-0.85) | Ref. 1.0 | 0.66 (0.29- 1.51) | 0.50 (0.17- 1.48) | 0.95 (0.32- 2.80) | 0.52 (0.30- 0.89) | Ref. 1.0 | 1.50 (0.43-5.21) | 1.44 (0.30-7.07) | 1.55 (0.32-7.58) | 2.09 (0.87-5.02) |
| HR_Adj3_ (95% CI) | Ref. 1.0 | 0.69 (0.56-0.84) | 0.68 (0.53-0.87) | 0.70 (0.53-0.92) | 0.74 (0.64-0.86) | Ref. 1.0 | 0.67 (0.29- 1.53) | 0.51 (0.17- 1.51) | 0.96 (0.32- 2.83) | 0.52 (0.30- 0.90) | Ref. 1.0 | 1.51 (0.43-5.28) | 1.45 (0.30-7.11) | 1.58 (0.32-7.76) | 2.09 (0.87-5.02) |
| Estimated relative fetal size classified according to Swedish national reference standards: small for gestational age (SGA) defined as <–2 SD from the mean, appropriate for gestational age (AGA) as within ±2 SD, and large for gestational age (LGA) as >+2 SD. HRs were estimated with Cox proportional hazards models. Crude (HR_cr_) and adjusted (HR_Adj_) models compare vacuum-assisted delivery (stratified by fetal head station: outlet, mid/low) and spontaneous vaginal delivery with emergency caesarean delivery as the reference category. HR_Adj1_: Adjusted for maternal age, smoking, maternal BMI, diabetes mellitus type 1 or 2, birthyear. HR_Adj2_: Adjusted for variables in model one and gestational diabetes, preeclampsia and chorioamnionitis. HR_Adj3_: Adjusted for variables of model 1 and 2 and additionally for maternal educational level and maternal pre-pregnancy comorbidities (ADHD, ASD, depression, anxiety disorder). CI = confidence interval; ECD, Emergency cesarean delivery; IR = incidence rate (per 1 000 person-years); SVD, Spontaneous vaginal delivery | | | | | | | | | | | | | | | |

## **Table T. Incidence and hazard ratios of intellectual disability** in children by mode of delivery, comparing vacuum-assisted delivery and spontaneous vaginal delivery with emergency caesarean delivery as reference, **stratified by estimated relative fetal size.**

|  | **Appropriate for gestational age** | | | | | **Small for gestational age** | | | | | **Large for gestational age** | | | | |
| --- | --- | --- | --- | --- | --- | --- | --- | --- | --- | --- | --- | --- | --- | --- | --- |
|  |  | **Vacuum-assisted delivery** | | |  |  | **Vacuum-assisted delivery** | | |  |  | **Vacuum-assisted delivery** | | |  |
|  | **ECD** | **Total** | **Outlet** | **Mid/Low** | **SVD** | **ECD** | **Total** | **Outlet** | **Mid/Low** | **SVD** | **ECD** | **Total** | **Outlet** | **Mid/Low** | **SVD** |
| **All** | | | | | | | | | | | | | | | |
| Event (n) | 499 | 396 | 236 | 160 | 2447 | 78 | 35 | 23 | 12 | 207 | 18 | 12 | 5 | 7 | 35 |
| IR (95% CI) | 0.51 (0.47-0.56) | 0.39 (0.35-0.43) | 0.40 (0.35-0.45) | 0.38 (0.32-0.43) | 0.35 (0.34-0.37) | 1.68 (1.31- 2.05) | 1.12 (0.75- 1.49) | 1.17 (0.69- 1.64) | 1.05 (0.45- 1.64) | 1.00 (0.86- 1.13) | 0.31 (0.17- 0.45) | 0.53 (0.23- 0.83) | 0.44 (0.05- 0.83) | 0.62 (0.16- 1.07) | 0.34 (0.22- 0.45) |
| HR_Cr_  (95% CI) | Ref. 1.0 | 0.76 (0.66-0.86) | 0.78 (0.67-0.91) | 0.73 (0.61-0.87) | 0.70 (0.63-0.77) | Ref. 1.0 | 0.65 (0.44- 0.97) | 0.68 (0.43- 1.08) | 0.61 (0.33- 1.11) | 0.60 (0.46- 0.77) | Ref. 1.0 | 1.69 (0.81- 3.51) | 1.42 (0.53- 3.81) | 1.96 (0.82- 4.69) | 1.11 (0.63- 1.96) |
| HR_Adj1_ (95% CI) | Ref. 1.0 | 0.81 (0.71-0.94) | 0.84 (0.72-1.00) | 0.77 (0.63-0.93) | 0.71 (0.64-0.79) | Ref. 1.0 | 0.66 (0.43- 1.02) | 0.62 (0.37- 1.04) | 0.74 (0.40- 1.36) | 0.58 (0.44- 0.77) | Ref. 1.0 | 1.75 (0.81- 3.77) | 1.58 (0.58- 4.32) | 1.92 (0.75- 4.91) | 1.00 (0.54- 1.86) |
| HR_Adj2_ (95% CI) | Ref. 1.0 | 0.82 (0.71-0.95) | 0.85 (0.72-1.01) | 0.78 (0.64-0.94) | 0.72 (0.65-0.80) | Ref. 1.0 | 0.65 (0.42- 1.01) | 0.61 (0.36- 1.03) | 0.72 (0.39- 1.34) | 0.57 (0.43- 0.76) | Ref. 1.0 | 1.91 (0.88- 4.15) | 1.74 (0.63- 4.79) | 2.08 (0.81- 5.34) | 1.12 (0.59- 2.10) |
| HR_Adj3_ (95% CI) | Ref. 1.0 | 0.85 (0.74-0.98) | 0.88 (0.75-1.04) | 0.80 (0.66-0.97) | 0.74 (0.67-0.83) | Ref. 1.0 | 0.68 (0.44- 1.06) | 0.64 (0.38- 1.08) | 0.76 (0.41- 1.41) | 0.58 (0.44- 0.78) | Ref. 1.0 | 1.90 (0.87- 4.15) | 1.73 (0.63- 4.77) | 2.07 (0.80- 5.36) | 1.15 (0.61- 2.16) |
| **Boys** | | | | | | | | | | | | | | | |
| Event (n) | 322 | 252 | 151 | 101 | 1556 | 53 | 18 | 11 | 7 | 118 | 14 | 6 | 3 | 3 | 17 |
| IR  (95% CI) | 0.58 (0.52-0.64) | 0.44 (0.39-0.50) | 0.46 (0.39-0.53) | 0.42 (0.34-0.50) | 0.45 (0.43-0.47) | 2.10 (1.53- 2.66) | 1.04 (0.56- 1.53) | 1.01 (0.41- 1.61) | 1.10 (0.29- 1.92) | 1.14 (0.94- 1.35) | 0.47 (0.22- 0.71) | 0.55 (0.11- 0.99) | 0.54 (-0.07- 1.14) | 0.56 (-0.07- 1.20) | 0.37 (0.19- 0.55) |
| HR_Cr_  (95% CI) | Ref. 1.0 | 0.76 (0.64-0.89) | 0.79 (0.65-0.96) | 0.71 (0.57-0.89) | 0.79 (0.70-0.89) | Ref. 1.0 | 0.48 (0.28- 0.82) | 0.47 (0.24- 0.90) | 0.51 (0.23- 1.12) | 0.54 (0.39- 0.75) | Ref. 1.0 | 1.17 (0.45- 3.04) | 1.15 (0.33-4.00) | 1.19 (0.34- 4.15) | 0.81 (0.40- 1.64) |
| HR_Adj1_ (95% CI) | Ref. 1.0 | 0.83 (0.70-0.99) | 0.88 (0.71-1.07) | 0.77 (0.61-0.98) | 0.82 (0.72-0.93) | Ref. 1.0 | 0.47 (0.26- 0.84) | 0.39 (0.19- 0.84) | 0.59 (0.27- 1.31) | 0.55 (0.39- 0.78) | Ref. 1.0 | 1.28 (0.49- 3.39) | 1.26 (0.36- 4.42) | 1.31 (0.37- 4.61) | 0.73 (0.34- 1.57) |
| HR_Adj2_ (95% CI) | Ref. 1.0 | 0.84 (0.71-1.01) | 0.89 (0.72-1.09) | 0.78 (0.62-1.00) | 0.83 (0.73-0.95) | Ref. 1.0 | 0.47 (0.26- 0.84) | 0.39 (0.18- 0.84) | 0.59 (0.27- 1.32) | 0.55 (0.38- 0.79) | Ref. 1.0 | 1.40 (0.53- 3.71) | 1.40 (0.39- 4.98) | 1.39 (0.39- 4.92) | 0.80 (0.37- 1.75) |
| HR_Adj3_ (95% CI) | Ref. 1.0 | 0.88 (0.73-1.05) | 0.92 (0.75-1.13) | 0.82 (0.64-1.04) | 0.87 (0.76-0.99) | Ref. 1.0 | 0.50 (0.28- 0.90) | 0.43 (0.20- 0.92) | 0.61 (0.27- 1.35) | 0.57 (0.40- 0.82) | Ref. 1.0 | 1.49 (0.55- 4.01) | 1.47 (0.41- 5.32) | 1.50 (0.42- 5.35) | 0.85 (0.38- 1.89) |
| **Girls** | | | | | | | | | | | | | | | |
| Event (n) | 177 | 144 | 85 | 59 | 891 | 25 | 17 | 12 | 5 | 89 | 4 | 6 | 2 | 4 | 18 |
| IR  (95% CI) | 0.42 (0.36-0.48) | 0.32 (0.27-0.38) | 0.32 (0.26-0.39) | 0.32 (0.24-0.40) | 0.25 (0.24-0.27) | 1.18 (0.72- 1.64) | 1.22 (0.64- 1.79) | 1.36 (0.59- 2.12) | 0.97 (0.12- 1.82) | 0.86 (0.68- 1.03) | 0.14 (0.00- 0.28) | 0.51 (0.10- 0.92) | 0.35 (-0.13- 0.83) | 0.66 (0.01- 1.31) | 0.31 (0.17- 0.45) |
| HR_Cr_  (95% CI) | Ref. 1.0 | 0.76 (0.61-0.95) | 0.77 (0.59-1.00) | 0.76 (0.56-1.01) | 0.61 (0.52-0.72) | Ref. 1.0 | 1.01 (0.55- 1.88) | 1.13 (0.57- 2.25) | 0.81 (0.31- 2.12) | 0.73 (0.47- 1.13) | Ref. 1.0 | 3.54 (1.00- 12.53) | 2.44 (0.45- 13.30) | 4.57 (1.14- 18.29) | 2.22 (0.75- 6.57) |
| HR_Adj1_ (95% CI) | Ref. 1.0 | 0.79 (0.62-1.00) | 0.81 (0.61-1.06) | 0.76 (0.55-1.05) | 0.61 (0.51-0.72) | Ref. 1.0 | 1.11 (0.57- 2.16) | 1.14 (0.54- 2.43) | 1.06 (0.40- 2.81) | 0.68 (0.42- 1.11) | Ref. 1.0 | 4.00 (0.95- 16.93) | 3.19 (0.53- 19.31) | 4.81 (0.96- 24.01) | 2.34 (0.67- 8.23) |
| HR_Adj2_ (95% CI) | Ref. 1.0 | 0.79 (0.62-1.00) | 0.81 (0.61-1.06) | 0.76 (0.55-1.05) | 0.61 (0.51) | Ref. 1.0 | 1.08 (0.56- 2.11) | 1.12 (0.52- 2.38) | 1.03 (0.39- 2.73) | 0.67 (0.41- 1.09) | Ref. 1.0 | 4.72 (1.07- 20.88) | 3.63 (0.58- 22.69) | 5.96 (1.13- 31.53) | 2.85 (0.76- 10.71) |
| HR_Adj3_ (95% CI) | Ref. 1.0 | 0.81 (0.64-1.03) | 0.83 (0.63-1.10) | 0.78 (0.56-1.07) | 0.62 (0.52-0.74) | Ref. 1.0 | 1.10 (0.57- 2.15) | 1.13 (0.53- 2.40) | 1.06 (0.40- 2.83) | 0.66 (0.40- 1.09) | Ref. 1.0 | 4.78 (1.09- 20.97) | 3.60 (0.58- 22.41) | 6.16 (1.17- 32.49) | 2.82 (0.76- 10.52) |
| Estimated relative fetal size classified according to Swedish national reference standards: small for gestational age (SGA) defined as <–2 SD from the mean, appropriate for gestational age (AGA) as within ±2 SD, and large for gestational age (LGA) as >+2 SD. HRs were estimated with Cox proportional hazards models. Crude (HR_cr_) and adjusted (HR_Adj_) models compare vacuum-assisted delivery (stratified by fetal head station: outlet, mid/low) and spontaneous vaginal delivery with emergency caesarean delivery as the reference category. HR_Adj1_: Adjusted for maternal age, smoking, maternal BMI, diabetes mellitus type 1 or 2, birthyear. HR_Adj2_: Adjusted for variables in model one and gestational diabetes, preeclampsia and chorioamnionitis. HR_Adj3_: Adjusted for variables of model 1 and 2 and additionally for maternal educational level and maternal pre-pregnancy comorbidities (ADHD, ASD, depression, anxiety disorder). CI = confidence interval; ECD, Emergency cesarean delivery; IR = incidence rate (per 1 000 person-years); SVD, Spontaneous vaginal delivery | | | | | | | | | | | | | | | |

**Figure A. Cumulative incidence functions (CIFs)** for long-term neurodevelopmental outcomes by mode of delivery.

*Cumulative incidence functions for autism spectrum disorder (ASD), attention-deficit/hyperactivity disorder (ADHD), intellectual disability (ID), epilepsy (EP), and cerebral palsy (CP), stratified by mode of delivery. Blue lines represent emergency caesarean delivery (ECD) and red lines represent vacuum-assisted delivery (VAD). Follow-up time is shown in years.*


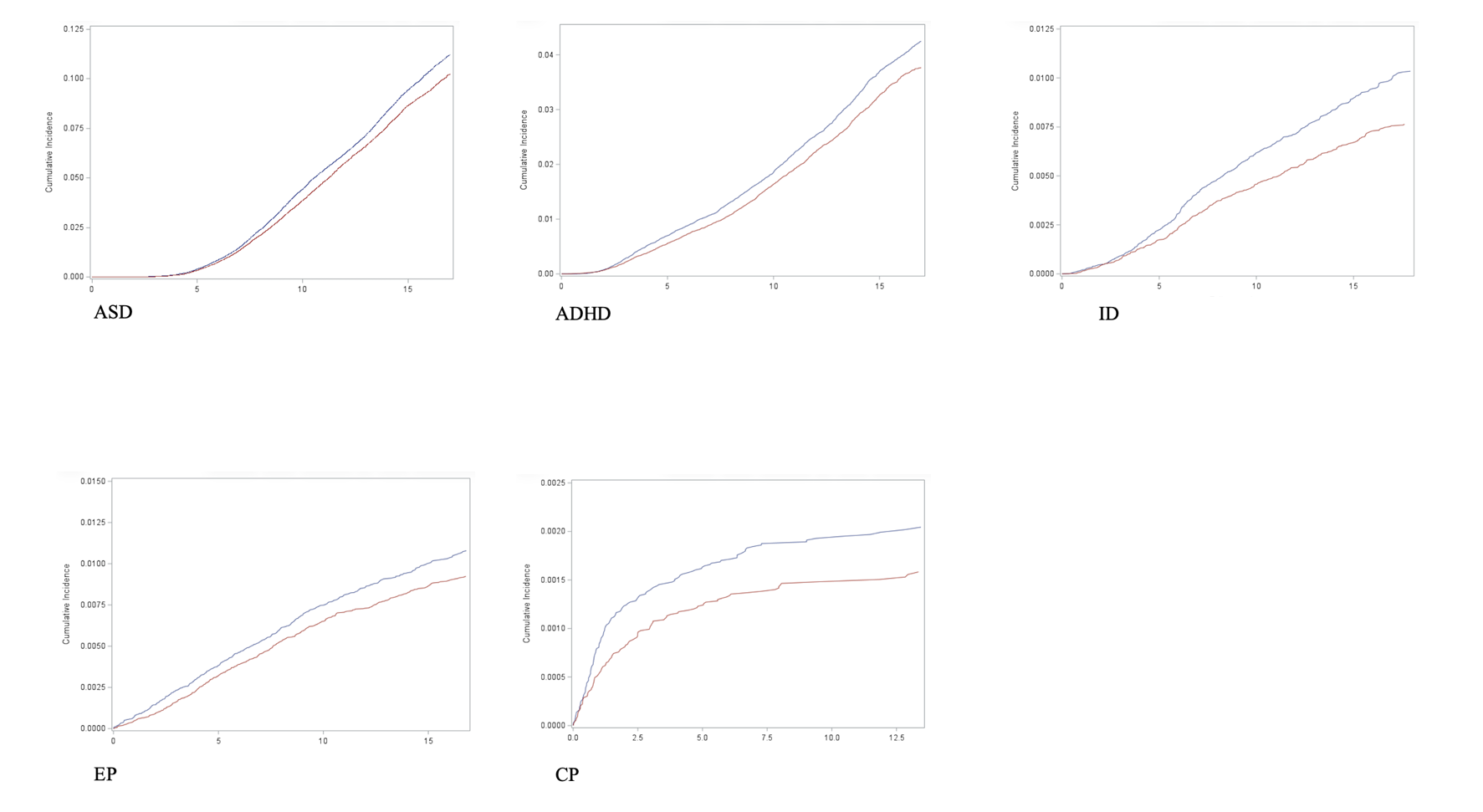

Supplement: S1 Appendix — (DOCX) [file pmed.1004825.s003.docx]
